# High concentrations of floating neustonic life in the plastic-rich North Pacific Garbage Patch

**DOI:** 10.1371/journal.pbio.3001646

**Published:** 2023-05-04

**Authors:** Fiona Chong, Matthew Spencer, Nikolai Maximenko, Jan Hafner, Andrew C. McWhirter, Rebecca R. Helm

**Affiliations:** 1 Energy and Environment Institute, University of Hull, Hull, United Kingdom; 2 School of Environmental Sciences, University of Hull, Hull, United Kingdom; 3 School of Environmental Sciences, University of Liverpool, Liverpool, United Kingdom; 4 International Pacific Research Center, School of Ocean and Earth Science and Technology, University of Hawaii at Manoa, Honolulu, Hawaii, United States of America; 5 Center for Marine Debris Research, Hawai’i Pacific University, Waimanalo, Hawaii, United States of America; 6 The Earth Commons, Georgetown University, Washington, DC, United States of America; University of Cambridge, UNITED KINGDOM

## Abstract

Floating life (obligate neuston) is a core component of the ocean surface food web. However, only 1 region of high neustonic abundance is known so far, the Sargasso Sea in the Subtropical North Atlantic gyre, where floating life provides critical habitat structure and ecosystem services. Here, we hypothesize that floating life is also concentrated in other gyres with converging surface currents. To test this hypothesis, we collected samples through the eastern North Pacific Subtropical Gyre in the area of the North Pacific “Garbage Patch” (NPGP) known to accumulate floating anthropogenic debris. We found that densities of floating life were higher inside the central NPGP than on its periphery and that there was a positive relationship between neuston abundance and plastic abundance for 3 out of 5 neuston taxa, *Velella*, *Porpita*, and *Janthina*. This work has implications for the ecology of subtropical oceanic gyre ecosystems.

## Abstract 2

Floating life (neuston) is a core component of the ocean surface food web, but the Sargasso Sea in the North Atlantic is the only known region of high neustonic abundance. This study reveals high densities of floating life in the plastic-rich Great Pacific Garbage Patch, suggesting that this area could be an important marine habitat.

## Introduction

**Fig 1:**
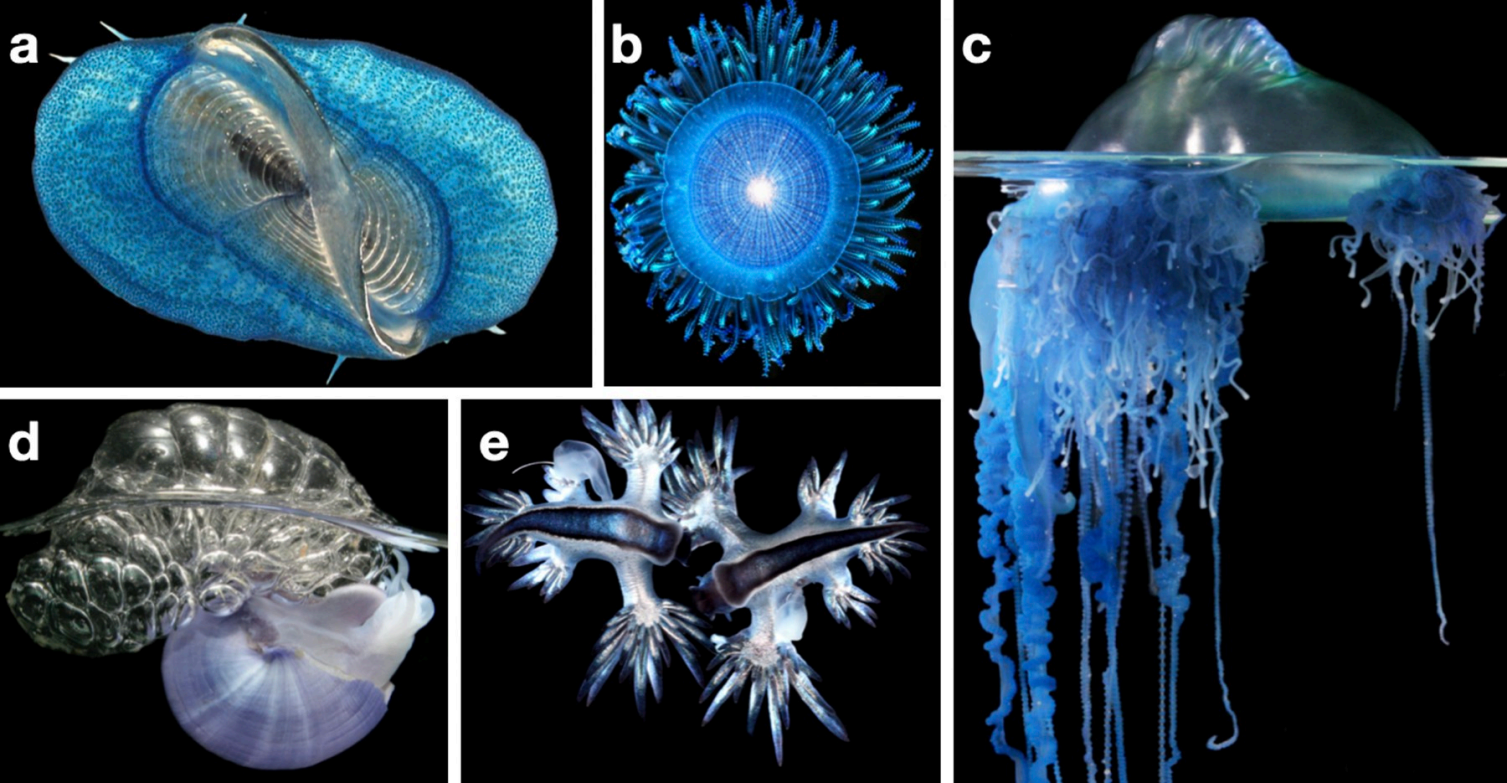
The neustonic organisms represented in this study, based on [1]. (a) Top-down view of by-the-wind sailor *Velella* sp. (b) Top-down view of blue button *Porpita* sp. (c) Side view of Portuguese man-o-war *Physalia* sp. (d) Side view of violet snail *Janthina* sp. (e) Top-down view of the blue sea dragons *Glaucus* sp. Images by Denis Riek.

Marine surface-dwelling organisms (obligate neuston) are a critical ecological link between diverse ecosystems [1], but we know very little about where these organisms are found. Obligate neuston includes multiple cnidarians and mollusks, as well as barnacles, copepods, and algae (Fig 1). All of these taxa are at the nexus of a surface food web that includes diverse sea birds, fish, and turtles. Hundreds of species that live in the water column, seafloor, or even in freshwater spend part of their lifecycle at the ocean’s surface (see review in [1]). As floating organisms, obligate neuston are transported and concentrated by ocean surface currents.

Many genera of neuston are globally distributed, but currently only 1 ocean region is known to concentrate neuston into high densities. The Sargasso Sea is named for the neustonic *Sargassum* algae and is a marine biodiversity hotspot supported by neuston. The Sargasso Sea is critical to the ecology of the North Atlantic and provides millions to billions of US dollars in ecosystem services annually [2,3]. But is the Sargasso Sea the only region of the world’s oceans where floating life concentrates?

Plastic pollution, transported by the same surface currents that transport neuston, provides a clue: Large amounts of floating debris are transported to and concentrated in “garbage patches” identified in all 5 main subtropical gyres, including the North Atlantic (the Sargasso Sea), South Atlantic, Indian Ocean, North Pacific, and South Pacific [4,5]. Obligate neuston, subjected to the same oceanographic forces that move buoyant man-made waste and pollutants, may also be concentrated in “garbage patches.” We hypothesize that these regions could be neuston seas, like the Sargasso Sea, and could provide similarly critical ecological and economic roles.

Convergence of obligate neustonic life into high densities may be critical for many neustonic species and the organisms that depend on them. Many obligate neuston, including foundational members of the neuston food web, *Physalia*, *Velella*, and *Porpita*, are incapable of swimming or directional movement. Predatory obligate neuston such as the blue sea dragon *Glaucus* and the violet snails *Janthina* also lack the ability to direct their movement and must physically bump into prey in order to feed [6,7]. Even more strikingly, *Glaucus* and possibly some species of *Janthina* must also be in physical contact to mate [8–10]. These adaptations point to the need for extremely high-density regions in order for these species to survive and reproduce. Some members of the neustonic community may also have adaptations to survive in relatively low nutrient waters (characteristic of many subtropical gyres [11]), including the presence of endosymbiotic zooxanthellae [12], similar to those found in corals (e.g., *Velella* and *Porpita;* Fig 1). Neuston are in turn consumed by diverse species [1] that may seek out dense concentrations as feeding grounds [13–15]. Identifying neuston hotspots can provide insights into the ecological dynamics of the wider region.

The North Pacific Garbage Patch (NPGP) is the largest and most infamous of the garbage patches [16]. It exists within the North Pacific Subtropical Gyre (NPSG), a massive region characterized in part by comparatively low nutrient densities [17,18]. Diverse neustonic species are documented from the NPSG [19–21], including several species of blue sea dragons (*Glaucus* spp.) for which this is the type locality [20]. While the NPGP has a dynamic spatial structure and exhibits significant variations temporarily, because it is thousands of miles from shore few surveys of neuston have been performed in this region.

To test our hypothesis that subtropical gyres and associated garbage patches may be neuston seas, including the NPGP, we conducted a community science survey through the NPGP with the sailing crew accompanying long-distance swimmer Benoît Lecomte (https://benlecomte.com/) as he swam through the NPGP (The Vortex Swim). The sampling scheme was coordinated through the use of a model that predicted the densities of floating objects. We found increased concentrations of floating life in the NPGP and a positive relationship between the abundance of floating life and floating plastic for 3 out of 5 neuston taxa. Ocean “garbage patches” and other convergence zones may be overlooked areas of high neuston abundance and could serve similar ecological roles to the North Atlantic Sargasso Sea, providing food and habitat for diverse species and valuable economic services. There is an urgent need to better understand these ecosystems and the role of plastic debris.

## Methods

**Fig 2:**
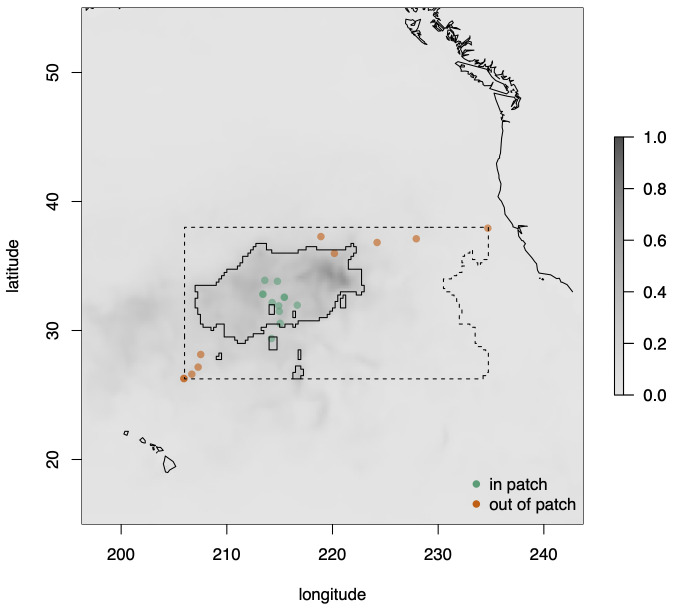
Dimensionless tracer concentration (shading) and locations of observations in the North Pacific in June–August 2019. Orange points lie outside the patch and green points inside (as defined a priori). There were 22 observations in total, but some symbols overlap because observations were close together in space. The dashed line encloses the region containing all points with tracer concentration at least as large as the minimum over all sites at which observations were made. This region is bounded by the smallest rectangle of latitude and longitude, parallel to the longitude axis that just encloses the sites at which observations were made. Solid lines enclose the region in the NPGP containing all points with tracer concentrations at least as large as the minimum over all sites at which observations were made within the patch. The data underlying this figure can be found in S1 Data. Map created in R using the maps package (https://cran.r-project.org/package=maps) and Natural Earth data (https://www.naturalearthdata.com/).

Neuston samples were collected by The Vortex Swim, an 80-day sailing expedition through the NPGP. A numerical drift model was used to plan the route of the expedition in accordance with regions of predicted high concentrations of floating plastic marine debris (Fig 2).

### Model tracer simulations

Accumulation of marine debris and neuston in the garbage patch was simulated in numerical experiments using velocities from the Surface Currents from Diagnostic (SCUD) model [22].

These velocities are derived from the historical dataset of drifter trajectories collected by the Global Drifter Program (https://www.aoml.noaa.gov/phod/gdp/) and include geostrophic currents, calculated from satellite altimetry, and wind-driven currents regressed to the local wind measured by satellite scatterometers (QuikSCAT and ASCAT). The use of Lagrangian data warrants adequate representation of the complex wind effects, combining turbulent mixing, Ekman currents, and Stokes drift due to wind waves. This model has been successfully used previously to simulate trans-Pacific drift of debris generated by the 2011 tsunami in Japan [23].

Anthropogenic debris originates from both land- and sea-based sources whose intensities and locations are not well documented. The influence of these uncertainties of the source on debris patterns is small in the garbage patches where debris items reside for a long time (e.g., [24]), during which they “forget” their origin. To simulate the garbage patch, a constant (in time and intensity) tracer input to the ocean was set up from all coastal grid points, and the model was looped between years 1992 and 2020 under a weak dissipation, representing degradation of debris due to physical factors (UV and storms) and biological interactions [25,26] until the model solution saturated to 95% (the root-mean-square difference between subsequent cycles). We used the 2 month mean concentration for July to August 2019 (the time period that most closely matched that of our observations) in subsequent analyses, to smooth out short-term fluctuations.

Conversion of model units into actual debris concentration is difficult due to the shortage of observational data and it is not necessary for our analysis. For practical applications, the model solution was scaled (non-dimensionalized) relative to the maximum concentration for July to August 2015 (the largest concentration over the sampling periods for our data and the data in [21]). The resulting map of dimensionless tracer concentrations is shown in Fig 2.

### Sampling

The Vortex Swim expedition aboard the sailing boat I Am Ocean started in June 2019 from Honolulu, Hawaii and reached San Francisco, California in August 2019. During this 80-day expedition, as part of a community science initiative, samples were taken for various scientific projects. Surface water neuston net samples were collected daily to assess microplastic concentration, of which 22 samples were photographed. Not all observations were photographed because of the haphazard nature of logistical constraints, such as crew availability. The plastic contents of an overlapping subset of samples were preserved and counted [27]. We show that there do not appear to be systematic differences in either plastic counts or tracer concentrations between sites from this subset that were and were not photographed (Fig B in S1 Appendix). Twelve photographs were taken in the central region of the NPGP and 10 were peripheral to or outside of the NPGP (Fig 2). Two different surface nets were used to collect microplastic and neuston samples throughout this expedition, a manta trawl and a neuston net. The manta trawl is designed so that the frame is above the water, while the net is fully submerged, with its wings keeping it from diving below the surface. The submerged dimensions of the mouth are 0.9 m × 0.15 m (width × height). This net had a mesh size of 500 μm and a codend with 100 μm mesh. The neuston net is designed to be towed so that only half of the mouth is submerged, with a full opening of 1 m × 0.5 m (width × height), only approximately 0.25 m depth of surface water was sampled. The neuston net had a mesh size of 333 μm for the net and the codend. Both nets function in a similar way, so we do not expect there to be a selection bias between nets. The net was towed along the sea surface for 30 min at each site at a speed of 1 to 2 knots (S1 Data). The contents of each tow were poured into a sieve that was of 333 μm mesh size. All plastic and neuston concentrations were standardized by surface area to accurately compare the results from both sampling methods.

Due to the fragility of neuston and the difficulty of sampling, biological preservation was not possible and we used a photographic survey for our analysis (unprocessed and processed images are available at https://doi.org/10.5281/zenodo.7510473). One image was taken per sample, with the exception of SJR_019, where 2 images were taken as organisms and plastic in the sample were much more abundant. All neustonic organisms, plastic, and other inorganic particles in each image were identified and counted by 2 independent observers using JMicroVision v1.3.2 [28]. Nothing below approximately 0.5 mm in the longest dimension was counted. Organisms were identified to the lowest taxonomic level possible: for all obligate neuston, this was to the genus level. Obligate neuston counted here consist of *Velella*, *Porpita*, *Janthina*, *Glaucus*, and *Physalia* (Fig 1 and Table 1).

**Table 1:** Organisms and objects observed from the image analysis of Vortex Swim manta trawl or neuston net samples.

Obligate neuston taxa	Other taxa	Inorganic objects
*Velella*	*Halobates*	Wood
*Porpita*	*Planes*	Rock
*Janthina*	Copepods	Rope
*Glaucus*	Unidentified blue dots	Plastic
*Physalia*	Fish	
	Nudibranch	
	Gastropod	
	Larvae	
	Ostracod	
	Isopod	
	Unknown crustacean	
	Unknown gelatinous organisms	

### Statistical analysis

We modeled the relationships between neuston and plastic counts and tracer concentration using a multivariate hierarchical Bayesian regression model (S1 Appendix). This approach accounts for the following key properties of the data: (1) observations are counts rather than densities and small counts are common; (2) sites were selected on the basis of tracer concentrations rather than at random; (3) the relationship between neuston and plastic densities may differ among neuston taxa and between locations inside and outside the NPGP; (4) study-specific sampling biases will affect the counts; and (5) the measurement process involved 2 independent counts from photographs. Full details are given in the Supporting information (Section C in S1 Appendix). For each category of object on each photograph, we modeled the pair of independent counts using a bivariate compound Poisson distribution [29] parametrized by a detection probability for each category on photographs (assumed the same for each observer) and an expected number of potentially visible objects (the product of expected density and area sampled). We modeled the log density of potentially visible objects as a multivariate linear function of the explanatory variables log tracer concentration (treated as known, because locations were selected on the basis of tracer concentration), patch membership (in or out of the NPGP, assigned a priori) and their interaction, with observation-level random effects drawn from a multivariate normal distribution, whose covariance matrix specifies the relationships between log density of each category of object conditional on the values of explanatory variables. We calculated the difference in expected log density of each category of object between the regions inside and outside the NPGP, averaged over the distributions of tracer concentrations in these regions, as described in the Supporting information (Section H in S1 Appendix). We calculated the marginal correlations between log density of each neuston taxon and log plastic concentration (and between total log neuston concentration and log plastic concentration) over the entire study region as described in the Supporting information (Section H in S1 Appendix). We also calculated separate marginal correlations for the regions inside and outside the NPGP. These statistics do not depend on the intercept for expected log density and are therefore unlikely to be strongly affected by sampling biases such as differences in catchability in nets or in detectability on photographs that determine whether absolute densities can be estimated. We estimated parameters using the NUTS algorithm [30] implemented in rstan version 2.21.5 [31]. Priors for each parameter are described in the Supporting information (Section F in S1 Appendix). Checks on the estimation method, model plausibility, and performance, including leave-one-out cross-validation, are described in the Supporting information (Section I in S1 Appendix).

We also fitted a similar model to data from Egger and colleagues [21]. We used a Poisson model for the count of each taxon in each of their observations, parametrized by the expected number, because in their data, there was only a single count (carried out on frozen samples in the laboratory) for each observation. We also divided the sampling area into 3 rather than 2 regions, as in their study. We used tracer concentrations from the 2-month periods that most closely matched the times of observations (July to August 2015 and November to December 2019). Full details are given in the Supporting information (Section J in S1 Appendix).

## Results

Observed neuston densities from locations in the central NPGP (median 3.44E4 km^-2^, first quartile 2.20E4 km^-2^, third quartile 6.96E4 km^-2^, based on the means of the 2 independent counts, summed over all taxa) appeared systematically higher than densities from locations peripheral to the NPGP (median 3.54E3 km^-2^, first quartile 6.53E2 km^-2^, third quartile 5.88E3 km^-2^).

**Fig 3:**
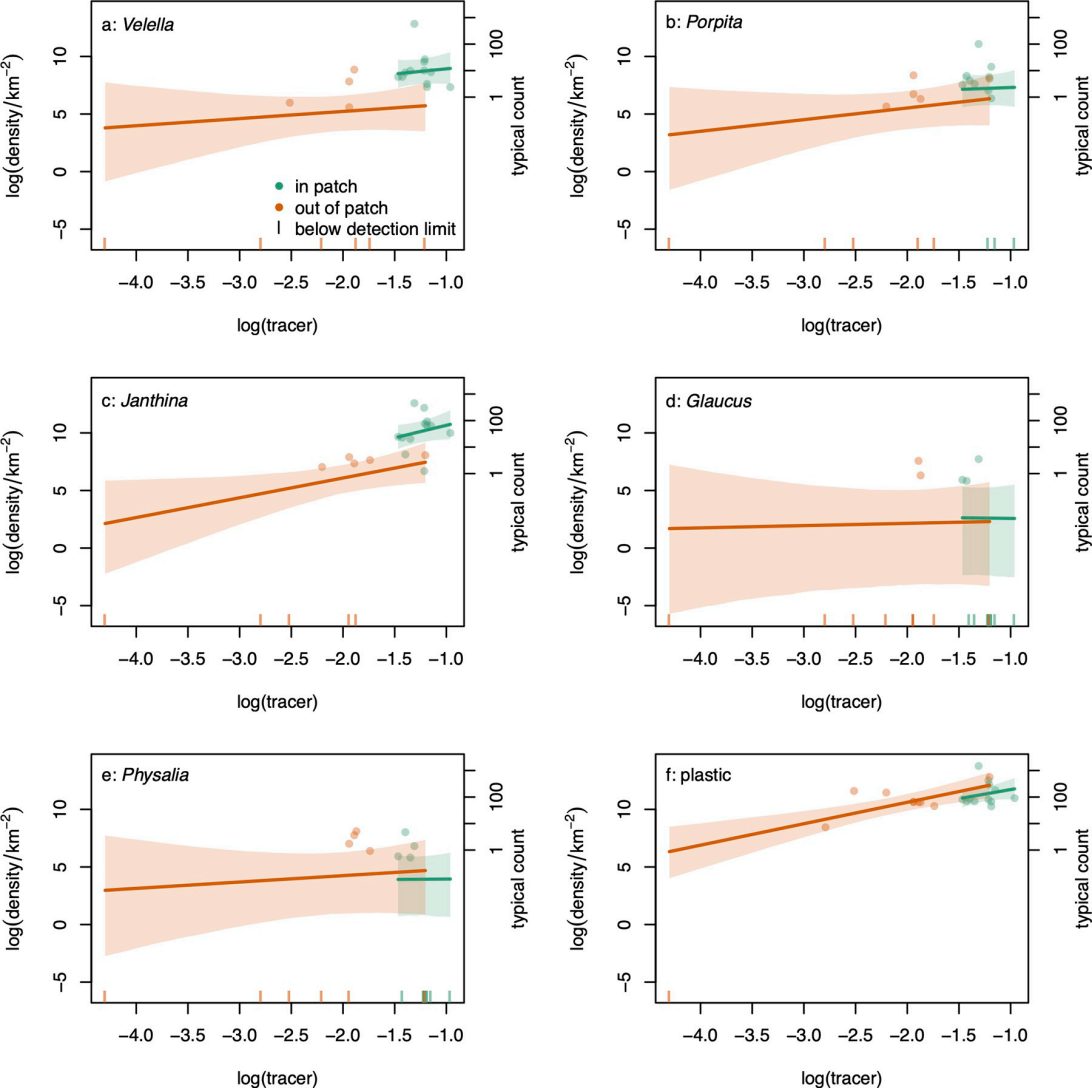
Relationship between natural log of density (in numbers km^−2^) and natural log of dimensionless tracer concentration for (a) *Velella*, (b) *Porpita*, (c) *Janthina*, (d) *Glaucus*, (e) *Physalia*, and (f) plastic outside (orange) and in (green) the patch. Points are sample means from 2 independent counts, with zeros plotted as vertical lines on the x-axis (note that models were fitted to the 2 counts, not the mean densities). Lines are posterior means, with 95% equal-tailed credible bands, and include the detectability parameters. The right-hand y-axis has tick marks at the log densities corresponding to counts of 1, 10, 100, and 1,000 objects in the mean trawled area. The data underlying this figure can be found in S1 Data.

The relationship between log density of each genus of neuston and plastic, and log tracer concentration was generally positive (Fig 3, slopes: we summarize the main model results here, but give full details Section K in S1 Appendix), and for *Velella*, *Porpita*, and *Janthina*, there was also a clear positive effect of being in the patch (Fig 3A–3C, orange versus green). For *Glaucus* and *Physalia*, there were many zero counts and the posterior mean relationship fell below the points with nonzero counts (Fig 5D and 5E). This does not indicate that the model fitted the observations poorly, rather that estimates of true density were reduced by observations with zero counts.

**Fig 4:**
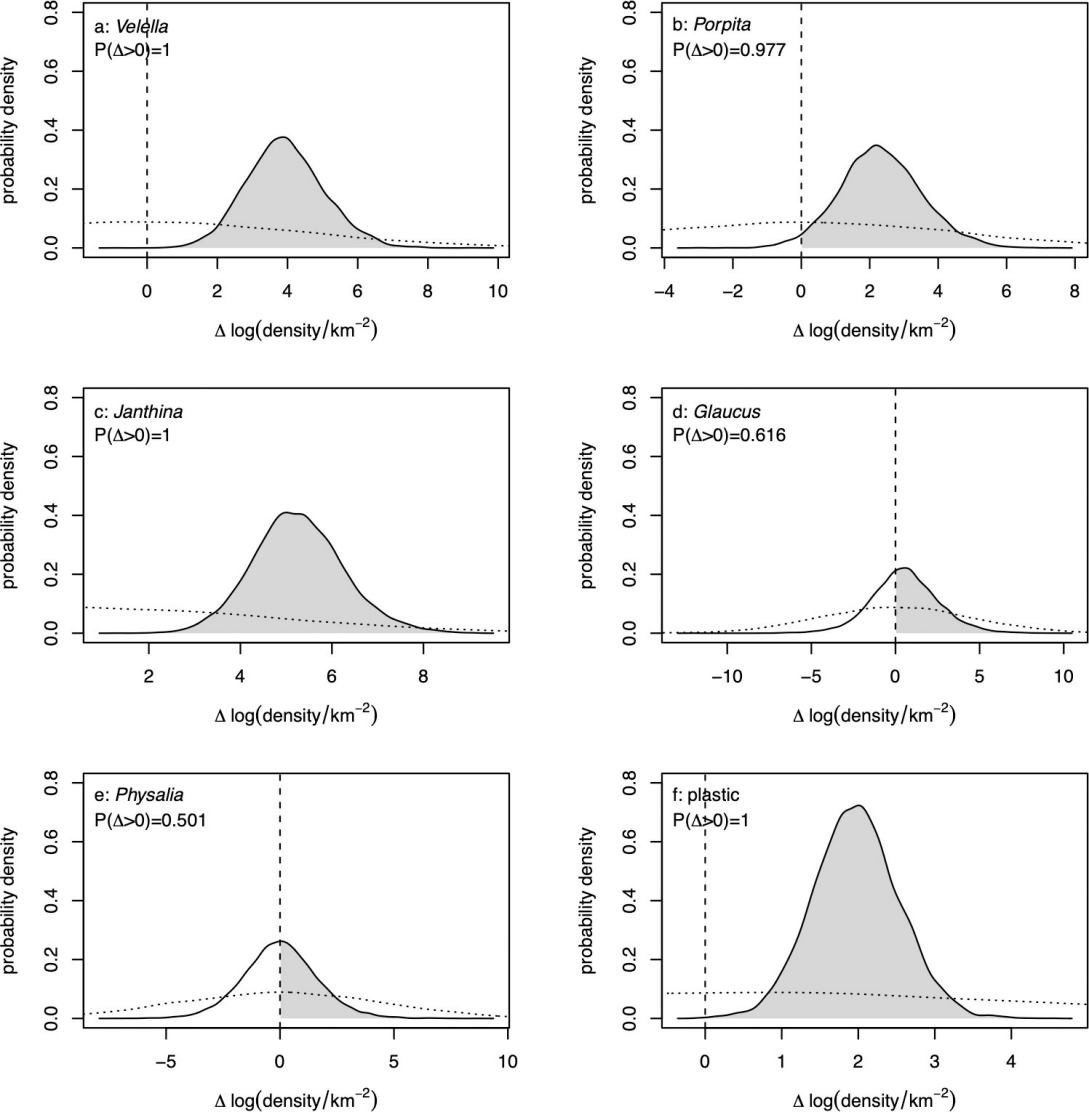
Difference Δ in expected natural log density (in numbers km^-2^) between the inside-patch and outside-patch regions for (a) *Velella*, (b) *Porpita*, (c) *Janthina*, (d) *Glaucus*, (e) *Physalia*, and (f) plastic. Posterior densities are represented as kernel density estimates, with vertical dashed lines at zero. The posterior probability that the difference is positive given on each panel. Dotted lines are kernel density estimates of the prior distribution for each difference induced by the priors on underlying parameters described in Section F in S1 Appendix. The data underlying this figure can be found in S1 Data.

The higher density of neuston inside the patch appears to be driven largely by 3 genera. Averaged over tracer concentrations, the expected natural log density was higher in the inside-patch region than the outside-patch region for *Velella*, *Porpita*, *Janthina*, and plastic (Fig 4A–4C and 4F). For the rarely captured taxa *Glaucus* and *Physalia*, the difference between inside- and outside-patch densities was centered on zero (Fig 4D and 4E). However, for all taxa, the posterior distribution of the difference was substantially more concentrated than the prior (Fig 4, solid versus dotted lines), so there was information in the data about these differences.

**Fig 5:**
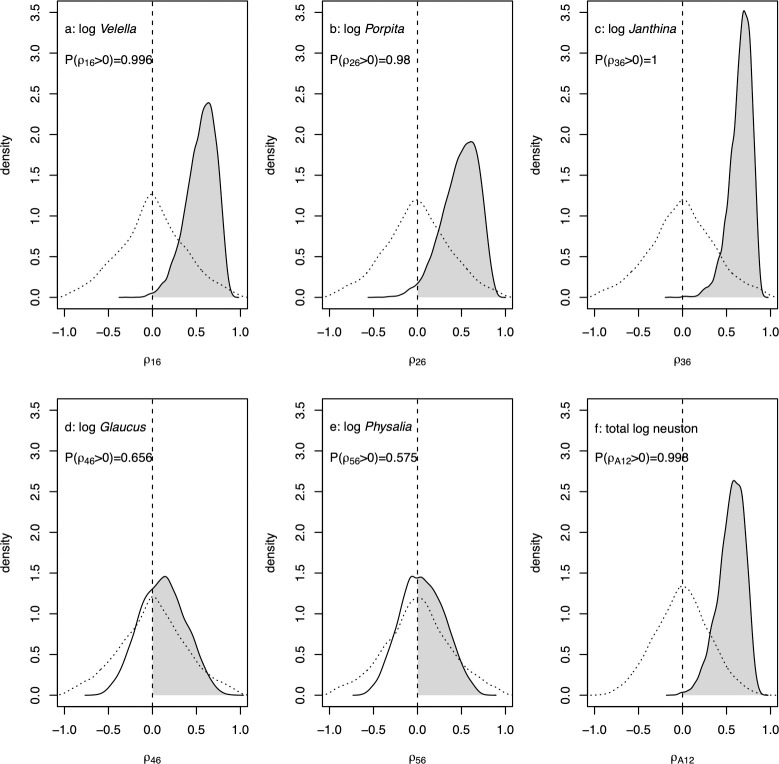
Posterior densities of marginal correlations ρ over the entire region between log plastic density and log densities of (a) *Velella*, (b) *Porpita*, (c) *Janthina*, (d) *Glaucus*, (e) *Physalia*, and (f) total log neuston. Posterior densities are represented as kernel density estimates, with vertical dashed lines at zero. The posterior probability that each marginal correlation is positive is indicated. Dotted lines are kernel density estimates of the prior distribution for each marginal correlation induced by the priors on underlying parameters described in section (Section F in S1 Appendix). The data underlying this figure can be found in S1 Data.

Posterior distributions of marginal correlations between log neuston density and log plastic density were almost entirely positive for *Velella*, *Porpita*, and *Janthina* (Fig 5A–5C). For the rare taxa *Glaucus* and *Physalia*, negative and positive marginal correlations with log plastic were about equally likely (Fig 5D and 5E). The posterior distribution of the marginal correlation between log plastic density and total log neuston was almost entirely positive (Fig 5F). Marginal correlations estimated separately for the regions inside and outside the NPGP were qualitatively similar to those for the whole region (Figs J and K in S1 Appendix).

Leave-one-out cross-validation showed that 1 in-patch observation with high densities of neuston and plastic (observation SJR_019) was very unlikely given the other observations, so that this observation was poorly fitted by the model. However, refitting the model without this observation did not substantially change the main results (Figs Q to T in S1 Appendix). In addition, 1 observation coded a priori as outside the patch on geographical grounds had a higher tracer concentration than some of those inside the patch (Fig 2, orange point inside top right of region bounded by solid line). Recoding this observation as inside did not substantially change the main results (Figs U to W in S1 Appendix). Other checks on model performance did not reveal any obvious problems. We were able to recover known parameters from simulated data (Figs M and N in S1 Appendix), except that there was little information in these data on absolute densities (Fig L in S1 Appendix), but as noted above, the statistics of interest do not require this information. Graphical posterior predictive checks suggested that data simulated under the model with posterior distributions of parameters appeared similar to the real data, so that overall model fit appeared adequate (Figs O and P in S1 Appendix).

Re-analysis of the Egger and colleagues [21] data showed that the area north-west of the NPGP (their area A) appeared to have more *Velella* but less *Porpita*, *Janthina*, and plastic than inside the NPGP (their area C, Fig X in S1 Appendix). However, the median count was zero for every taxon, which may have contributed to the lack of information on many quantities of interest. Averaged over tracer concentrations, more *Velella*, *Porpita*, and plastic but less *Janthina*, *Glaucus*, and *Physalia* were found inside the NPGP (their area C) than peripheral to the NPGP (their area B) (Fig Z in S1 Appendix). More *Janthina*, *Glaucus*, *Physalia*, and plastic but less *Velella* were found in the periphery (their area B) than outside the NPGP (their area A) (Fig AA in S1 Appendix). Marginal correlations between log neuston densities and log plastic density across the whole study region were negative for *Velella* and positive for *Janthina* (Fig AB in S1 Appendix). Within the 3 areas, these marginal correlations were weakly positive for *Velella* and weakly negative for *Janthina*. For other taxa, there was little information in these data on correlations with plastic (Figs AC to AE in S1 Appendix).

## Discussion

Our data suggest higher concentrations of floating life and plastic inside than outside the NPGP, and positive correlations between the logs of neuston concentrations and the log of plastic concentration for 3 out of 5 neuston taxa, *Velella*, *Porpita*, and *Janthina*. The obligate neustonic taxa *Velella*, *Porpita*, and *Janthina* may be concentrated by the same physical forces that concentrate plastic within the region and these concentrations may be important for the ecology of these species. A limited number of studies have examined obligate neuston in this region, so it is difficult to infer processes and patterns by comparing them, especially because neuston concentrations in this region may vary seasonally or annually. Nevertheless, the possible overlap between garbage patches and neuston seas has important implications for established and emerging high seas impacts and activities.

Physical forces may be partly responsible for our observed distribution and abundance of obligate neuston, and these concentrations may be important for neuston life history. Physical forces are responsible for the high concentration of plastics in the NPGP [32], and in the North Atlantic subtropical gyre are responsible for concentrating neustonic *Sargassum* algae in the Sargasso Sea [33]. Within our study, a patchy distribution of neuston and plastic at the surface may be due to small-scale (sub-mesoscale) physical surface dynamics such as slicks. We found the highest concentration of both neuston and plastic in a slick (observation SJR_019), and this is true for other studies as well. For example, off the coast of the island of Hawai'i, nearly 40% of surface-associated larval fish, 26% of surface invertebrates, and 95.7% of plastic were found in surface slicks, which represented only 8% of the sea surface area of the West Hawai'i study region [13–14]. In the North Atlantic, neustonic *Sargassum* is often concentrated in slicks under appropriate conditions [34,35]. Sea surface slicks create a relatively small area where diverse species come into physical contact through drifting. Because neustonic predators such as *Janthina* and *Glaucus*, both found in our study, rely on physically contacting prey [1,6,36,37], and similarly *Glaucus* spp. and likely some members of the genus *Janthina* depend on direct physical contact to mate [8–10], regional concentrations and small-scale surface slicks may be an important habitat feature for neustonic organisms. In our study, we found evidence that obligate neuston may also be reproducing in the NPGP: in at least 1 sample, we found many small *Velella* roughly 0.5 cm in length and *Janthina* sp. and *Porpita* sp. less than 1 mm in length. Based on a growth estimate for *Velella*, the small *Velella* in our sample may be approximately 5 to 16 days old [38].

More and better data will be needed before strong conclusions can be drawn about neuston distributions in the NPGP, and methodological differences may account for some of the apparent differences in results between this study and Egger and colleagues [21]. The ad hoc study design for our data, common to many community science projects, is a weakness. Randomized sampling is logistically difficult in this environment, but lattice designs may be feasible and are often considered suitable for the study of spatial patterns [39]. It will also be important to ensure that enough objects of interest are collected. In both studies, there was little information on relationships between neuston and plastic for taxa with low counts (and in the Egger and colleagues [21] data, the median count was zero for every taxon). Future work should also account for spatial structure in the sampling design. Our analyses assumed independent and identically distributed observation-level random effects. Where observations are clustered in space (as in some of the data used by Egger and colleagues [21], where most of the observations consisted of sets of 3 trawls very close together), a hierarchical error structure could account for this clustering. More generally, a spatially structured covariance model such as a Matérn function [39], possibly based on distances from a transport model rather than Euclidean distance, might be appropriate. We did not pursue these ideas here because the low sample sizes and (in the case of the Egger and colleagues [21]) low counts would make estimation difficult. Additionally, different counting approaches should be evaluated. We modeled the photographic sampling process used in our study, but because there was little information in the data on detectability, we cannot say much about absolute densities. On the other hand, the process of freezing, shipping, and then counting samples used by Egger and colleagues [21] might reduce the counts of soft-bodied species relative to hard-bodied organisms (R. Helm, personal observation). Immediate counting of fresh samples may be the most reliable method, where possible. Direct comparison of these approaches before designing future studies would be useful.

We expect neuston abundance to vary over time, due to differences in morphology, anatomy, sizes, and life history of individuals and species. For example, we observed higher densities of *Velella* within the patch, while for the Egger and colleagues [21] data, higher densities of *Velella* were found outside the patch. However, *Velella* come in 2 different forms, with sails that either tilt to the left (NW-type) or right (SW-type). Savilov [19] observed a higher abundance of NW type *Velella* outside the patch and SW type *Velella* inside the patch, meaning that the observations of Egger and colleagues [21] may have sampled 2 morphologically different *Velella* populations. Neither our study nor Egger and colleagues [21] examined orientation type, though this may be an important biological difference for *Velella*. For *Janthina*, both studies found higher densities in and around the patch than outside it. *Janthina*, like small plastics, is likely not moved by the wind to the same degree as the wind-harnessing *Velella*, and this may be why both studies observed *Janthina* inside the NPGP. Future studies also need to account for seasonal variation. For example, we already know that there are seasonal aggregations of *Velella* off the coast of California [40], but know much less about within-patch seasonality. Differences in observed neuston abundance between studies could also be due to interannual variability. In our study, a regional chlorophyll bloom occurred in the NPSG near our sampling, and although our sampling did not overlap with this observed bloom, this increased primary productivity in the subtropical gyre may be related to our comparatively high observed neuston densities [41]. Neuston may also interact with plastic in the patch or with communities growing on plastic. For example, the sea skater insect *Halobates* may increase in abundance due to the presence of plastic, on which it lays its eggs [42]. Rafting organisms [43], which grow on large plastic debris, may also interact with obligate neuston, though it is not clear yet what the nature of these interactions may be. Neuston may also consume microplastics, similar to rafting barnacles [44], though this has not been documented for neuston, and the effects, if any, may be challenging to detect. Regardless, the impact of plastic on the surface environment in this region is worth future study.

Our findings suggest that subtropical gyres and other areas of high plastic concentration may be more than just garbage patches, and that these regions may serve important ecosystem functions as “neuston seas.” Obligate neuston are present in the diet of a variety of species, including those that are known to ingest plastic, such as sea turtles [45,46] and the Laysan albatross [47]. In the North Atlantic Sargasso Sea, the neustonic ecosystem is a feeding ground, a nursery ground, and a habitat [15]. Similar to the Sargasso Sea, our results suggest the central NPGP has high surface life densities relative to surrounding waters, yet much is still unknown about the ecology of these organisms. Studies on the food webs and life history of neustonic species will allow us to better understand their temporal cycles and connectivity. It is also important for high seas industries and emerging high sea activities to consider their impacts on the ocean’s surface ecosystem [48]. Lastly, our study highlights the value of community science and its importance in studying life at the air–sea interface.

## Supporting information

S1 Appendix Supplemental information on statistical approaches, including additional figures and equations. (PDF)

S1 Data All data and code necessary to replicate our results. (ZIP)
