# Supplementary material for: High concentrations of floating neustonic life in the plastic-rich North Pacific Garbage Patch
Source: PLoS Biol. 2023 May 4;21(5):e3001646. doi: 10.1371/journal.pbio.3001646 (PMC10159152; doi:10.1371/journal.pbio.3001646)
Supplement: S1 Appendix — (PDF) [file pbio.3001646.s001.pdf]

Supporting information: high concentrations of floating neustonic life in the plastic-rich North Pacific Garbage Patch

Fiona Chong

Matthew Spencer

Nikolai Maximenko

Jan Hafner

Andrew C. McWhirter

Rebecca R. Helm

A Motivation

1. Observations are counts, not densities. Empirical estimates of density can be obtained by dividing the count by the area trawled. However, when the expected number of objects in a given category caught in a trawl is low, chance variation in the number actually caught can have a large effect on the estimated density, and it is inappropriate to treat densities as continuous.

2. Sites were selected on the basis of modelled tracer concentrations. Thus, tracer concentration is not a random variable. In addition, the data needed for this study were not available from every site because of operational constraints.

3. It is likely that the relationship between neuston and plastic densities differs among neuston taxa.

4. It is plausible that the relationship between neuston and plastic densities differs between areas with different oceanographic conditions, and these areas are also likely to differ in tracer concentrations.

5. All studies will be subject to study-specific sampling biases that lead to consistent over- or under-counting of particular components. For example, it is likely that visual counts will underestimate the number of plastic particles, while counts from frozen samples will underestimate the numbers of soft-bodied neuston taxa.

6. The measurement process in our data involved visual counts of objects on photographs by two independent observers.

B Basic model

We will first consider the basic case with a single set of counts for each observation, and then refine the model to account for the measurement process. Let $\mathbf{y}_i = (y_{i1}, y_{i2}, \ldots, y_{i,m-1}, y_{i,m})$ be the counts of $m-1$ neuston taxa (the first $m-1$ values) and plastic particles ($y_{i,m}$) in the ith observation, $i = 1, \ldots, n$). Let a_i be the area trawled in the i observation, x_i be the centered and scaled log of modelled concentration of the tracer at the location of the ith observation, and p_i be an indicator variable taking the value 1 if the observation is inside the patch and 0 otherwise. We will model the relationship between $\mathbf{y}$ and x using a multivariate Poisson-lognormal regression [1]:

$$\begin{aligned} y_{ij} &\sim \text{Poisson}(a_i \lambda_{ij}), \\ \lambda_{ij} &= e^{\eta_{ij}}, \\ \boldsymbol{\eta}_i &= \boldsymbol{\beta}_0 + \boldsymbol{\beta}_1 x_i + \boldsymbol{\beta}_2 p_i + \boldsymbol{\beta}_3 x_i p_i + \boldsymbol{\varepsilon}_i, \\ \boldsymbol{\varepsilon}_i &\sim N(\mathbf{0}, \boldsymbol{\Sigma}). \end{aligned} \tag{A}$$

Here, λ_{ij} is the rate (numbers L^{-2}) for the jth variable in the ith observation, $\boldsymbol{\eta}_i$ is the $(m+1)$-dimensional linear predictor for the ith observation, $\boldsymbol{\beta}_0$ is the intercept vector, $\boldsymbol{\beta}_1$ is the coefficient vector for the effect of log tracer concentration, $\boldsymbol{\beta}_2$ is the coefficient vector for the effect of patch membership, $\boldsymbol{\beta}_3$ is the coefficient vector for the interaction between tracer concentration and patch membership, and $\boldsymbol{\varepsilon}_i$ is a multivariate normal observation-level random effect, with mean vector $\mathbf{0}$ and covariance matrix $\boldsymbol{\Sigma}$. This random effect represents unpredictable effects such as small-scale spatial variability. We assume that the $\boldsymbol{\varepsilon}_i$ are identically distributed, independent of each other and of explanatory variables, and that the counts y_{ij} are conditionally independent, given the trawled areas a_i and rates λ_{ij}.

The use of a Poisson observation model for the counts y_{ij} (with an offset a_i to account for variation in trawled area among observations) addresses point 1. A linear predictor on the log scale is the natural choice for count data, to ensure that expected values are positive. We use log tracer concentration rather than tracer concentration as an explanatory variable, because we expect that the modelled physical processes determining tracer concentration are similar to those determining expected neuston and plastic densities, so that it makes sense to apply the same transformation to tracer concentration as to expected neuston and plastic densities. The observation-level random effects $\boldsymbol{\varepsilon}_i$ account for overdispersion, which is likely to be important (for example, because slicks may concentrate floating objects in some areas much more than others). The use of a regression model, with log tracer concentration x_i as a non-random explanatory variable, addresses point 2. The use of a multivariate model, in which each neuston taxon

is distinguished, addresses point 3. The inclusion of the interaction term $\boldsymbol{\beta}_3 x_i p_i$ allows the relationship between tracer concentration and densities of neuston and plastic to differ between areas. Below, we show how to calculate the relationships between the logs of expected neuston and plastic densities over an area with a given distribution of tracer concentration, addressing point 4. We also show that these relationships are not affected by consistent study-specific biases, addressing point 5. We will develop a more detailed model of the measurement process below to address point 6.

C Measurement process

In our data, the material from each trawl sample was photographed, and visual counts of objects on each photograph were done independently by two observers. Not every site was photographed, due to operational constraints that occurred on a haphazard basis, such as availability of crew. However, we believe that this is unlikely to have led to systematic biases. To check this, we compared plastic densities for sites that were and were not photographed, over a subset of sites for which these densities were available from another source [5]. For 44 sites visited during the study (those coded `SJR` in the original data file), plastic particles were picked out, preserved and later counted by hand [5]. Of these 44, 8 were also photographed (and thus included in our data set), while 36 were not (Figure A).

We extracted data on total plastic densities from the supporting information of Egger et al. [5]. There did not appear to be systematic differences in plastic density between sites that were or were not photographed (Figure B). The sample maximum was greater for those sites that were not photographed, but this is likely to be a consequence of the larger sample size of unphotographed sites.

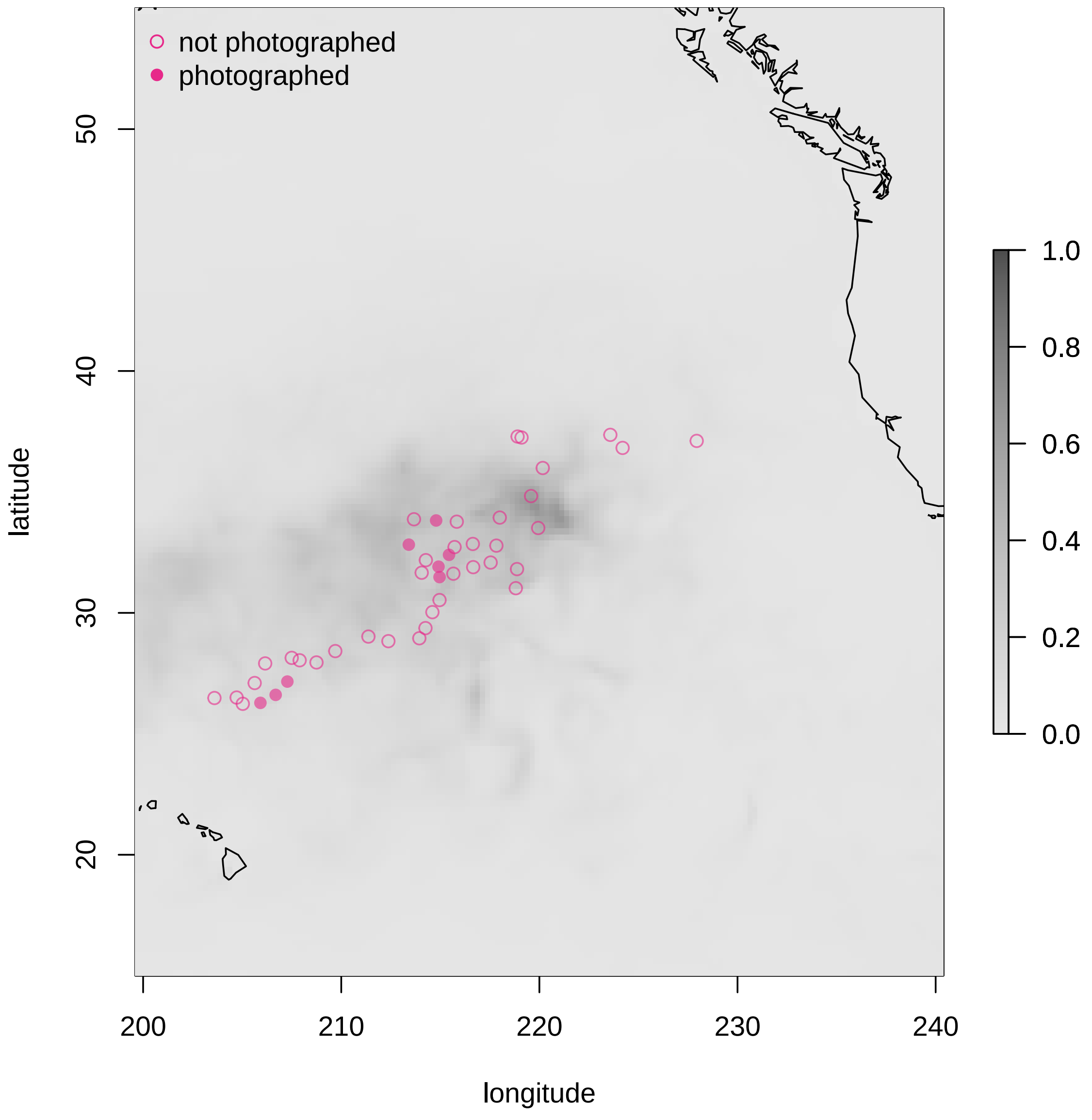

Figure A: Locations of 36 sites that were not photographed (open circles), and 8 that were (filled circles). Plastic counts for all these sites (but not for other sites in our data set), obtained by preserving and hand counting, were included in Egger et al. [5]. Shading represents dimensionless tracer concentration in July 2019. The data underlying this Figure can be found in S1 Data. Map created in R using the maps package (`https://cran.r-project.org/package=maps`) and Natural Earth data (`https://www.naturalearthdata.com/`).

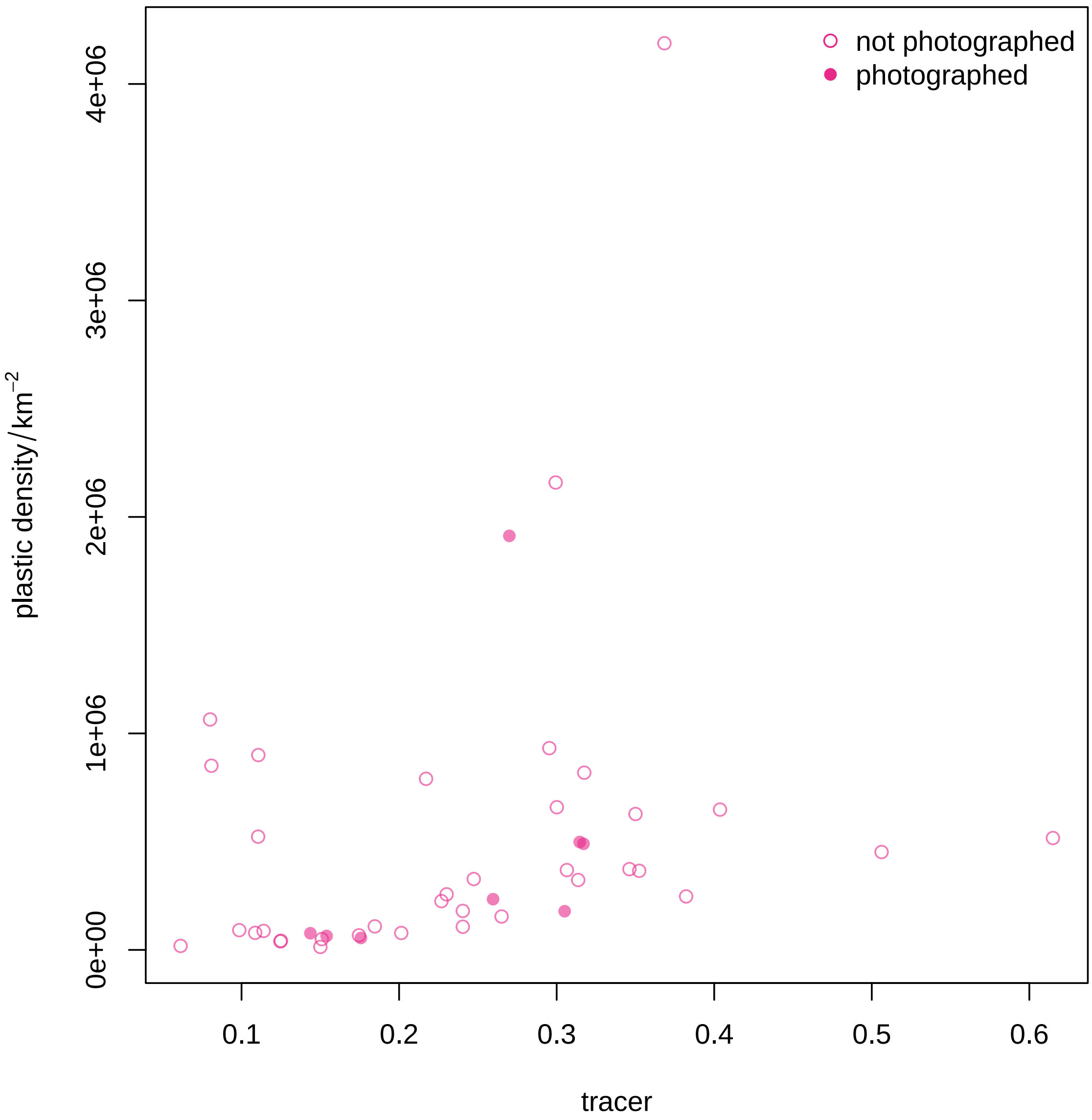

Figure B: Relationships between total plastic count per km^2 and dimensionless tracer concentration for 36 sites that were not photographed (open circles), and 8 that were (filled circles). Data from Egger et al. [5]. The data underlying this Figure can be found in S1 Data.

For those sites that were photographed, independent visual counts were made from the photographs by two observers. There did not appear to be systematic differences between the observers (Figure C), except that observer FC may have counted more *Janthina* than observer RH (Figure Cc). We assume that the dominant mode of error in counting is failing to record every object in a given category, rather than putting objects in the wrong categories. This is plausible given that the number of objects was sometimes large, but the appearances of the different categories were relatively distinct.

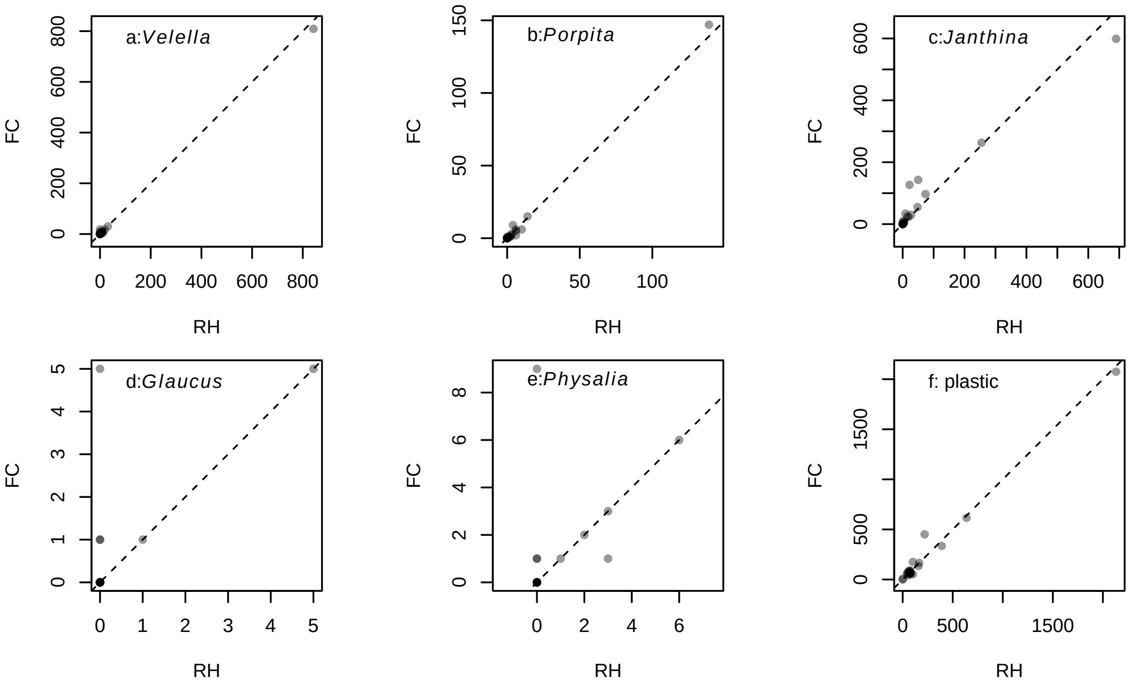

Figure C: Comparison of visual counts of a: *Velella*, b: *Porpita*, c: *Janthina*, d: *Glaucus*, e: *Physalia* and f: plastic in each trawl sample, by two observers (RH, FC). The dashed lines correspond to equal counts. Note the different axis scales on each panel. The data underlying this Figure can be found in S1 Data.

We model failure to record every object by assigning a detection probability κ_j to each category of object. We assume that this probability is the same for both observers, and for every object in the category. We first show how to determine the distribution of counts if there is only a single observer, and then develop the corresponding results for two observers. We then suggest that the overall sampling model for our data should be considered as a two-stage process, with the single-observer stage applying to the number of potentially visible items on a photograph, and the two-observer stage applying to counts from the photograph.

For a single observer, let z_{ij} be the count for category j in observation i. The corresponding Poisson-distributed true count y_{ij} from Equation A is unobserved. The assumption of a constant κ_j leads to a binomial distribution for the random variable Z_{ij} representing the observed count, conditional on the true count. This is known as a binomial-Poisson hierarchy distribution [3, p. 163], for which the marginal

probability of observing count z_{ij} is:

$$\begin{aligned}\mathbb{P}(Z_{ij} = z_{ij}) &= \sum_{k=0}^{\infty} \mathbb{P}(Z_{ij} = z_{ij} | Y_{ij} = k)\, \mathbb{P}(Y_{ij} = k) \\ &= \sum_{k=z_{ij}}^{\infty} \binom{k}{z_{ij}} \kappa_j^{z_{ij}} (1-\kappa_j)^{k-z_{ij}} \frac{(a_i\lambda_{ij})^k e^{-a_i\lambda_{ij}}}{k!} \\ &= \frac{(a_i\kappa_j\lambda_{ij})^{z_{ij}} e^{-a_i\kappa_j\lambda_{ij}}}{z_{ij}!},\end{aligned} \tag{B}$$

which is the probability of observing z_{ij} under a Poisson distribution with parameter $a_i\kappa_j\lambda_{ij}$. Because the detection probabilities κ_j only appear in a product with the rates λ_{ij}, only this product can be estimated. In other words, detection probabilities will be absorbed into the intercept $\boldsymbol{\beta}_0$ in Equation A, and cannot be estimated from a single-count study.

When there are two independent observers, let $z_{1,ij}$ and $z_{2,ij}$ be the counts for category j in observation i by observers 1 and 2 respectively. These counts are conditionally independent, given the true number of the jth category in the ith observation. Thus the marginal probability of observing counts $(z_{1,ij}, z_{2,ij})$ is

$$\begin{aligned}\mathbb{P}(Z_{1,ij} = z_{1,ij}, Z_{2,ij} = z_{2,ij}) &= \sum_{k=0}^{\infty} \mathbb{P}(Z_{1,ij} = z_{1,ij}, Z_{2,ij} = z_{2,ij} | Y_{ij} = k)\, \mathbb{P}(Y_{ij} = k) \\ &= \sum_{k=0}^{\infty} \mathbb{P}(Z_{1,ij} = z_{1,ij} | Y_{ij} = k)\, \mathbb{P}(Z_{2,ij} = z_{2,ij} | Y_{ij} = k)\, \mathbb{P}(Y_{ij} = k) \\ &= \sum_{k=z_{1,ij}}^{\infty} \binom{k}{z_{1,ij}} \kappa_j^{z_{1,ij}} (1-\kappa_j)^{k-z_{1,ij}} \binom{k}{z_{2,ij}} \kappa_j^{z_{2,ij}} (1-\kappa_j)^{k-z_{2,ij}} \frac{(a_i\lambda_{ij})^k e^{-a_i\lambda_{ij}}}{k!}.\end{aligned}$$

This is a bivariate compound Poisson distribution with the detection probability κ_j the same for the two observers, for which

$$\mathbb{P}(Z_{1,ij} = z_{1,ij}, Z_{2,ij} = z_{2,ij}) = \exp\left[-a_i\lambda_{ij}(1-(1-\kappa_j)^2)\right] \sum_{k=0}^{\min(z_{1,ij}, z_{2,ij})} \frac{(a_i\lambda_{ij}\kappa_j(1-\kappa_j))^{z_{1,ij}+z_{2,ij}-2k}(a_i\lambda_{ij}\kappa_j^2)^k}{(z_{1,ij}-k)!(z_{2,ij}-k)!k!}. \tag{C}$$

[7]. Properties of this and related distributions are given in Johnson et al. [9, chapter 36, section 8]. Note that unlike the single-count model, the detection probabilities κ_j do not simply appear in a product with the rates λ_{ij}, suggesting that it may be possible to estimate the detection probabilities from a double-count study.

The overall sampling model should be interpreted as a two-stage process. The number of objects potentially visible on a photograph should be interpreted as being drawn from a binomial-Poisson hierarchy distribution (Equation B), conditional on the rate for the site, with detection probabilities that cannot be identified, leading to a Poisson distribution of potentially visible items on the photograph conditional on the rate. Conditional on the photograph, the distribution of the number of objects counted

by the observers will be bivariate compound Poisson (Equation C) with detection probabilities applying to detection of objects on the photograph. Thus our full model, based on Equation A but with bivariate compound Poisson observations, is

$$\begin{aligned}(z_{1,ij}, z_{2,ij}) &\sim \text{bivariate compound Poisson}(\kappa_j, a_i\lambda_{ij}),\\ \lambda_{ij} &= e^{\eta_{ij}},\\ \boldsymbol{\eta}_i &= \boldsymbol{\beta}_0 + \boldsymbol{\beta}_1 x_i + \boldsymbol{\beta}_2 p_i + \boldsymbol{\beta}_3 x_i p_i + \boldsymbol{\varepsilon}_i,\\ \boldsymbol{\varepsilon}_i &\sim N(\mathbf{0}, \boldsymbol{\Sigma}).\end{aligned} \tag{D}$$

D Conditional and marginal covariance

It is natural to work on the log scale, and study covariance of $\boldsymbol{\eta}$. The conditional covariance of η_i given (p_i, x_i) is simply $\boldsymbol{\Sigma}$.

To determine the marginal covariance between the logs of expected neuston and plastic densities over some area Ω with a given distribution of log tracer concentration and patch membership, let $\boldsymbol{\mu}_{\boldsymbol{\eta}}$ be the expected value of the linear predictor over this area:

$$\boldsymbol{\mu}_{\boldsymbol{\eta}} = \mathbf{B}\boldsymbol{\mu}_X, \tag{E}$$

where $\mathbf{B} = [\boldsymbol{\beta}_0, \boldsymbol{\beta}_1, \boldsymbol{\beta}_2, \boldsymbol{\beta}_3]$ is the $m \times 4$ matrix whose columns are the coefficient vectors, and $\boldsymbol{\mu}_X = [1, \mu_X, \mu_P, \mu_{XP}]^T$, where μ_X, μ_P and μ_{XP} are the means of log tracer, patch membership and the product of log tracer and patch membership respectively, over the area of interest and ^{T} denotes transpose. The deviation of any given $\boldsymbol{\eta}_i$ from the mean over the area is $\boldsymbol{\eta}_i - \boldsymbol{\mu}_{\boldsymbol{\eta}} = \mathbf{B}(X_i - \boldsymbol{\mu}_X) + \boldsymbol{\varepsilon}_i$, where $X_i = [1, x_i, p_i, x_i p_i]^T$. The the marginal covariance matrix $\boldsymbol{\Psi}$ over this area is

$$\begin{aligned}\boldsymbol{\Psi} &= \mathbb{E}\left[(\boldsymbol{\eta}_i - \boldsymbol{\mu}_{\boldsymbol{\eta}})(\boldsymbol{\eta}_i - \boldsymbol{\mu}_{\boldsymbol{\eta}})^T\right]\\ &= \mathbb{E}\left[(\mathbf{B}(X_i - \boldsymbol{\mu}_X) + \boldsymbol{\varepsilon}_i)(\mathbf{B}(X_i - \boldsymbol{\mu}_X) + \boldsymbol{\varepsilon}_i)^T\right]\\ &= \mathbf{B}\,\mathrm{V}(X)\mathbf{B}^T + \boldsymbol{\Sigma}\\ &= \mathbf{B}_{-1}\,\mathrm{V}(X_{-1})\mathbf{B}_{-1}^T + \boldsymbol{\Sigma},\end{aligned} \tag{F}$$

where $\mathbf{B}_{-1} = [\boldsymbol{\beta}_1, \boldsymbol{\beta}_2, \boldsymbol{\beta}_3]$ is the coefficient vector with the intercept $\boldsymbol{\beta}_0$ dropped, $X_{-1} = [x_i, p_i, x_i p_i]^T$ is the vector of explanatory variables excluding the constant element 1, and $\mathrm{V}(X_{-1})$ is the covariance matrix of this vector over the area of interest. Note that because the intercept effect is constant over the area, it has no effect on the marginal covariance. Thus study-specific biases in sampling that lead to consistent over- or under-counting of particular components are irrelevant.

If we wish to aggregate neuston taxa into a vector $\boldsymbol{\eta}_A$ of total log neuston and log plastic, we can

write

$$\boldsymbol{\eta}_A = \mathbf{A}\boldsymbol{\eta},$$

where $\mathbf{A}$ is the $2 \times m$ matrix

$$\mathbf{A} = \begin{bmatrix} 1 & 1 & \dots & 1 & 0 \\ 0 & 0 & \dots & 0 & 1 \end{bmatrix}.$$

Then the covariance matrix $\boldsymbol{\Psi}_A$ of total log neuston and log plastic is

$$\begin{aligned} \boldsymbol{\Psi}_A &= \mathbf{A}\boldsymbol{\Psi}\mathbf{A}^T \\ &= \mathbf{A}\mathbf{B}_{-1}\,\mathrm{V}(X_{-1})\mathbf{B}_{-1}^T\mathbf{A}^T + \mathbf{A}\boldsymbol{\Sigma}\mathbf{A}^T, \end{aligned} \tag{G}$$

which again is unaffected by study-specific biases that lead to consistent over- or under-counting of particular components. Total log neuston is proportional to the log of the geometric mean of neuston densities, and is not equal to the log of total neuston (for which there will not be a similarly simple expression for marginal covariance with log plastic).

E Tracer regions

The marginal correlation calculations in section D depend on appropriate choices of region. We want to avoid extrapolating too far beyond the geographical area in which observations were taken, or the range of tracer concentrations over these observations. Here, we describe how we achieved this.

Let $\mathcal{R}$ be the smallest rectangle of latitude and longitude, aligned with the longitude axis, that encloses the sites at which observations were made. We assume that these sites have been divided *a priori* into those inside and those outside the patch. Let $\Omega_{\text{all}} = \{r \in \mathcal{R} : x_r \geq m_{\text{all}}\}$, where m_{all} is the minimum log tracer concentration over all the sites at which observations were made (Figure D, region bounded by dashed line). Similarly, let $\Omega_{\text{in}} = \{r \in \mathcal{R} : x_r \geq m_{\text{in}}\}$, where m_{in} is the minimum log tracer concentration over all the sites that lie within the patch (Figure D, region bounded by solid lines). Then let $\Omega_{\text{out}} = \Omega_{\text{all}} \setminus \Omega_{\text{in}}$. Note that these regions are not necessarily either simply connected or connected (and in fact Ω_{in} and Ω_{out} are neither).

The regions Ω_{all} and Ω_{in} contain tracer concentrations greater than those at any of our observations. We therefore also considered versions in which cells with tracer concentrations above the maximum at any of our observations were removed. However, the results from these were almost indistinguishable from those with the regions defined as above, so we do not report them here.

In practice, tracer concentrations were available on a grid of cells with latitude and longitude resolution $0.25°$, and therefore not of equal area. Thus, within each region, the means and variances in Equations E, F and G were weighted by cell area. Tracer concentrations for observations were taken as

those in the grid cells corresponding to the start location of each trawl.

F Priors

We used Bayesian methods to fit the model defined by Equation D. Here, we describe the prior distribution for each parameter.

For the intercept $\boldsymbol{\beta}_0$, it is thought that *Porpita* can reach densities of up to 2 individuals m^{-2}, and that it, along with *Velella*, is among the neuston taxa that can achieve the highest densities [12, p. 395]. Thus, log densities in km^{-2} as high as 14.5 are plausible. However, in unsuitable conditions very low densities of less than one individual km^{-2} are also plausible. We therefore chose independent $N(0, 7.5)$ priors on the elements of $\boldsymbol{\beta}_0$ (throughout, we parametrize univariate normal distributions by standard deviation rather than variance).

For the effect $\boldsymbol{\beta}_1$ of log tracer concentration, it seems plausible on physical grounds that the relationship between tracer concentration and densities of plastic and neuston could be close to linear. Thus 1 is a plausible value for the elements of $\boldsymbol{\beta}_1$. Values close to 0 are also plausible, if the tracer model does not correctly capture the processes determining plastic and neuston densities. Values higher than 2 would be surprising, because there is little physical reason to expect quadratic or higher-powered polynomial relationships. We therefore chose independent $N(0, 1)$ priors on the elements of $\boldsymbol{\beta}_1$.

For the effect $\boldsymbol{\beta}_2$ of patch, we think that up to 1000-fold differences in density (approximately 6.9 on the natural log scale) between locations inside and outside the patch might be plausible, but no difference is also plausible once tracer concentration has been accounted for. We therefore chose independent $N(0, 4)$ priors on the elements of $\boldsymbol{\beta}_2$.

For the coefficient $\boldsymbol{\beta}_3$ of the interaction between tracer and patch, both 0 and 1 should be plausible, but values higher than 2 would be surprising, as for the tracer effect. We therefore chose independent $N(0, 1)$ priors on the elements of $\boldsymbol{\beta}_3$.

We have little information on the detection probabilities κ_i, but they can only be between 0 and 1. We therefore chose independent flat Beta$(1, 1)$ priors on each κ_i.

For the covariance matrix $\boldsymbol{\Sigma}$ of observation-level random effects, we followed a common approach to priors for multivariate hierarchical models [13, section 1.13]. We decomposed the prior into a diagonal matrix of coefficient scales and a correlation matrix. For the scales, we chose independent weakly-informative independent half-Cauchy$(0, 2.5)$ priors. For the correlation matrix, we chose an LKJ prior with shape parameter 2, which weakly concentrates around the identity matrix [10].

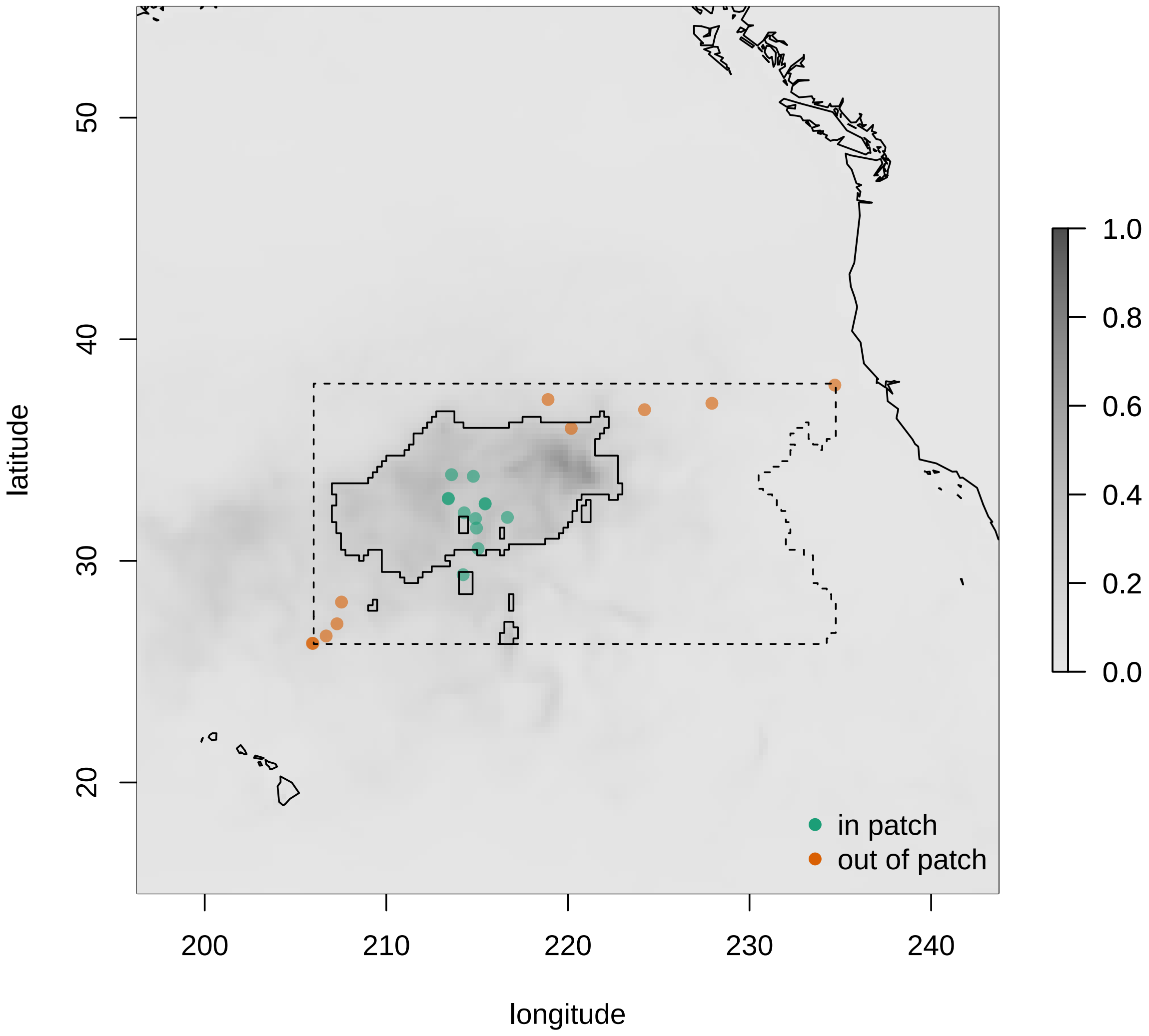

Figure D: Dimensionless tracer concentration (shading) and locations of observations in the North Pacific. Orange points lie outside the patch, and green points inside (as defined *a priori*). The dashed line encloses the region Ω_{all}, containing all points with tracer concentration as least as large as the minimum over all sites at which observations were made, and within the smallest rectangle $\mathcal{R}$ of latitude and longitude, parallel with the longitude axis, that just encloses the sites at which observations were made. Solid lines enclose the region Ω_{in}, containing all points in $\mathcal{R}$ with tracer concentrations at least as large as the minimum over all sites at which observations were made within the patch. Note that Ω_{in} is neither simply connected nor connected. The data underlying this Figure can be found in S1 Data. Map created in R using the maps package (`https://cran.r-project.org/package=maps`) and Natural Earth data (`https://www.naturalearthdata.com/`).

G Estimation

We fitted the model defined by Equation D with priors as in Section F using the `NUTS` algorithm [8], implemented in the R package `rstan` version 2.21.5 [14], with data preparation and post-processing done in R version 4.2.1 [11]. We ran 4 chains for 2000 warmup and 2000 sampling iterations each. This took approximately 5 h on a 64-bit Ubuntu 20.04.4 system with 4 Intel Core i7-4600M 2.9 GHz cores and 15.3 GiB RAM. We set the maximum tree depth to 20 and the `adapt_delta` parameter to 0.95, as these choices helped to avoid divergent transitions in preliminary versions of the model. Effective sample size was at least 3399, and the $\hat{R}$ statistic was no larger than 1.0014, for all parameters. Inspection of trace plots did not suggest any obvious problems with convergence.

H Visualization

To visualize the relationship between log density and log tracer concentration, we plotted the log of observed density (number of objects km^{-2}) for each category of object against the log of tracer concentration for each observation. We plotted densities rather than counts to correct for differences in trawled area among observations. Observed densities were calculated from the mean of the two independent counts for each observation. Where both counts were zero, we plotted the point on the x-axis. We added lines (with equal-tailed 95% credible bands) representing the posterior mean predicted density, corrected for detectability, conditional on log tracer concentration and patch membership: $\log \boldsymbol{\kappa} + \boldsymbol{\beta}_0 + \boldsymbol{\beta}_1 x + \boldsymbol{\beta}_2 p + \boldsymbol{\beta}_3 xp$. We corrected for detectability by including $\log \boldsymbol{\kappa}$, because observed densities will be affected by detectability.

To understand the effects of increased log tracer concentration on log density inside and outside the patch in more detail, we plotted the posterior distributions of $\boldsymbol{\beta}_1 + \boldsymbol{\beta}_3$ and $\boldsymbol{\beta}_1$ respectively, and calculated the posterior probabilities that these effects were positive. Values of particular interest are 0 (no relationship between log density and log tracer) and 1 (density proportional to tracer, expected on physical grounds if the tracer model captures the processes affecting density). These values were indicated on the plots.

To visualize the difference in predicted log density between inside-patch and outside-patch regions, we calculated expected log densities $\boldsymbol{\mu}_{\text{in}}$ and $\boldsymbol{\mu}_{\text{out}}$ over the inside- and outside-patch regions:

$$\begin{aligned}\boldsymbol{\mu}_{\text{in}} &= \mathbb{E}_{\Omega_{\text{in}}}[\boldsymbol{\beta}_0 + \boldsymbol{\beta}_2 + (\boldsymbol{\beta}_1 + \boldsymbol{\beta}_3)x], \\ \boldsymbol{\mu}_{\text{out}} &= \mathbb{E}_{\Omega_{\text{out}}}[\boldsymbol{\beta}_0 + \boldsymbol{\beta}_1 x],\end{aligned}$$

where the expectations were weighted by cell area. Then the difference in predicted log density is $\boldsymbol{\Delta} = \boldsymbol{\mu}_{\text{in}} - \boldsymbol{\mu}_{\text{out}}$. For each category, we plotted the posterior distribution of this difference (as a kernel

density estimate), and calculated the posterior probability that the difference was positive.

To visualize the marginal relationships between log densities of each taxon and plastic, we calculated the marginal covariances between log density of each taxon and log plastic density, and between total log neuston and log plastic, using Equations F and G respectively, and standardized to correlations $\boldsymbol{\rho}$. We did these calculations for the three regions Ω_{all}, Ω_{in} and Ω_{out}. We plotted the posterior distributions of these correlations (as kernel density estimates), and calculated the posterior probability that each correlation was positive.

For differences in predicted log density between inside-patch and outside-patch regions, and marginal correlations, we determined how much information there was in the data by plotting the prior distributions of these statistics, and comparing them visually with the posterior distributions. We estimated prior distributions by taking a sample (of the same size as the posterior sample) from the priors for each parameter in Stan using the `Fixed_param` algorithm, and then applying the same calculations to this prior sample as to the posterior sample.

I Checks on estimation method, model plausibility and performance

We checked whether the detectability parameters $\boldsymbol{\kappa}$ can be estimated from these data (and thus whether there is absolute density information) by examining scatter plots of the bivariate posterior distributions of the elements of $\boldsymbol{\beta}_0$ against the corresponding elements of $\log \boldsymbol{\kappa}$. Equation C suggests that in principle it may be possible to estimate detectability. However, it seems likely that at least to some extent, a high intercept could compensate for low detectability, and vice versa. Thus, strong negative relationships between corresponding elements of $\boldsymbol{\beta}_0$ and $\log \boldsymbol{\kappa}$ would suggest difficulties in estimating detectability. The Hessian of the log of the probability mass function can also given information on this [4]. If increases in the intercept can completely compensate for decreases in detectability, the log probability mass function will have a ridge of constant values along a negative relationship between log detectability and the intercept. If this happens, the Hessian will not be of full rank. We therefore evaluated the Hessian of the log of the probability mass function for counts (Equation C) with respect to κ_j and $a_i\lambda_{ij}$, at posterior mean estimates. If detectabilities cannot be estimated, we will not have information on absolute densities (but note that the statistics of interest do not depend on absolute densities).

We checked the ability of the estimation method to recover known parameters by generating 10 simulated data sets from Equation D, with the same values of explanatory variables as the real data, and each parameter set to its posterior mean. We then estimated parameters from the simulated data sets as above, except that we used `cmdstan 2.30.1` [2] rather than `rstan`, for ease of automation via a bash script. We plotted posterior densities for each parameter from each simulated data set, with true

parameters and prior densities indicated on the plots. We also calculated the proportion of simulated data sets for which the 95% highest posterior density interval for each parameter contained the true value. This approach will help to rule out major errors in coding, and will give a rough idea of whether the estimation method is working, but cannot tell us whether the estimated posterior densities are exactly correct. Computation took approximately 60 h on a 64-bit Ubuntu 20.04 system with 4 Intel Xeon 3.2 GHz cores and 16 GiB RAM. Ideally, we would have used simulation-based calibration [15] to determine whether the entire posterior densities are correct, but this would have been too time-consuming given the length of time needed to estimate parameters from a single data set.

We carried out a graphical posterior predictive check on the plausibility of the model. For each of 200 iterations, we generated a simulated data set with the same values of explanatory variables as the real data, and a set of parameter values drawn from the posterior distribution. We plotted the relationship between log density and log tracer concentration in the same way as for the observed data. Systematic differences between the observed and simulated relationships will indicate ways in which the model fails.

As an additional posterior predictive check, we examined the correlation between the counts from each observer for each taxon. This correlation is a key feature of the bivariate count distribution that is not captured by the relationship between log density and log tracer concentration. We therefore plotted the posterior predictive distribution of this correlation for each taxon from each of 200 iterations, and overlaid the observed correlation.

We used leave-one-out cross-validation to estimate the out-of-sample predictive performance of the model. Let $f(\mathbf{z}_{1,l}, \mathbf{z}_{2,l})$ be the predictive density for the lth observation (consisting of a pair of count vectors $\mathbf{z}_{1,l}$ and $\mathbf{z}_{2,l}$), and let $\boldsymbol{\theta}_{-l}$ be the posterior density of the full set of parameters estimated from all observations other than l. For each of 1000 draws from this leave-one-out density, we estimated the log predictive density of the new observation l, integrated over the distribution of the observation-level random effect $\boldsymbol{\varepsilon}_l$:

$$\int \log f(\mathbf{z}_{1,l}, \mathbf{z}_{2,l} | \boldsymbol{\eta}_l, a_l, x_l, p_l, \boldsymbol{\kappa}) f(\boldsymbol{\eta}_l | \boldsymbol{\varepsilon}_l, \boldsymbol{\theta}_{-l}) f(\boldsymbol{\varepsilon}_l | \boldsymbol{\theta}_{-l}) \, \mathrm{d}\boldsymbol{\varepsilon}.$$

We estimated this integral by classical Monte Carlo, with a sample size of 1×10^5. We plotted the distribution of these estimates of log predictive density for each observation. Observations which are unusual given the rest of the data are likely to have low log predictive density and may indicate ways in which the model is inadequate. This computation took approximately 66 h on a 64-bit Ubuntu 20.04 system with 4 Intel Xeon 3.2 GHz cores and 16 GiB RAM to re-fit the model to each leave-one-out data set, followed by approximately 12 h on a 64-bit Ubuntu 20.04.4 system with 4 Intel Core i7-4600M 2.9 GHz cores and 15.3 GiB RAM to integrate over the distribution of $\boldsymbol{\varepsilon}$. Popular methods such as Pareto-smoothed importance sampling [16] would be much faster, but are not available for our model because of the observation-level random effects.

J Analysis of Egger et al. [6] data

Egger et al. [6] report data from a similar survey in the North Pacific. They collected data from 54 trawls, of which 9 were taken in 2015 and 45 in 2019. Here, we follow Egger et al. [6] in ignoring the differences among years. They classified their sites *a priori* into three areas A, B and C based on their own modelled plastic concentrations, with A having the lowest modelled plastic concentrations and C the highest. The contents of trawls were frozen and later counted by hand in the laboratory. We analyzed these data using a model based on Equation A:

$$\begin{aligned} y_{ij} &\sim \text{Poisson}(a_i \lambda_{ij}), \\ \lambda_{ij} &= e^{\eta_{ij}}, \\ \boldsymbol{\eta}_i &= \boldsymbol{\beta}_0 + \boldsymbol{\beta}_1 x_i + \boldsymbol{\beta}_B p_{B,i} + \boldsymbol{\beta}_C p_{C,i} + \boldsymbol{\beta}_{1,B} x_i p_{B,i} + \boldsymbol{\beta}_{1,C} x_i p_{C,i} + \boldsymbol{\varepsilon}_i, \\ \boldsymbol{\varepsilon}_i &\sim N(\mathbf{0}, \boldsymbol{\Sigma}). \end{aligned} \tag{H}$$

Here, p_B and p_C are indicator variables for being in areas B and C respectively, with associated coefficients $\boldsymbol{\beta}_B$ and $\boldsymbol{\beta}_C$, and coefficients of interactions with log tracer concentration $\boldsymbol{\beta}_{1,B}$ and $\boldsymbol{\beta}_{1,C}$. Detectability effects such as degradation of organisms in frozen samples could be modelled as a binomial-Poisson hierarchy, for which the detectability parameters cannot be identified (Equation B), and thus do not lead to any change in model structure compared to Equation A. Locations were clustered, with sets of 3 trawls taken close together in 2019, and sets of 1 or 2 trawls taken close together in 2015. Here, we ignore this clustering for simplicity, although it might be more appropriate to introduce an additional cluster-level random effect to account for this.

We defined tracer regions using a similar approach to section E. We took the geographical region $\mathcal{R}$ to be the smallest rectangle of latitude and longitude, aligned with the longitude axis, that enclosed all the observations from both years (because the model was fitted to all these data). We selected the minimum tracer concentrations defining each region based on data from each year in turn, but did subsequent calculations using only the 2019 regions, because most of the observations were from 2019. Let $\Omega_{\text{all}} = \{r \in \mathcal{R} : x_{r,2019} \geq m_{\text{all}}\}$, where $x_{r,2019}$ is the log tracer concentration in cell r in 2019 and m_{all} is the lowest log tracer concentration over any observation in 2019 (Figure Ea, region bounded by dotted line). Let $\Omega_C = \{r \in \mathcal{R} : x_{r,2019} \geq m_C\}$, where m_C is the minimum log tracer concentration over any observation in area C in 2019 (Figure Ea, region bounded by solid lines). Let $\Omega_{BC} = \{r \in \mathcal{R} : x_{r,2019} \geq m_B\}$, where m_B is the minimum log tracer concentration over any observation in areas B or C in 2019 (Figure Ea, region bounded by dashed lines).Then let $\Omega_A = \Omega_{\text{all}} \setminus \Omega_{BC}$ and $\Omega_B = \Omega_{BC} \setminus \Omega_C$. We computed marginal correlations over the regions Ω_{all}, Ω_C, Ω_B and Ω_A for 2019. Similar regions were defined for 2015 (Figure Eb), but were not used in subsequent calculations (and in fact all observations in 2015 were from area C, so these regions coincide).

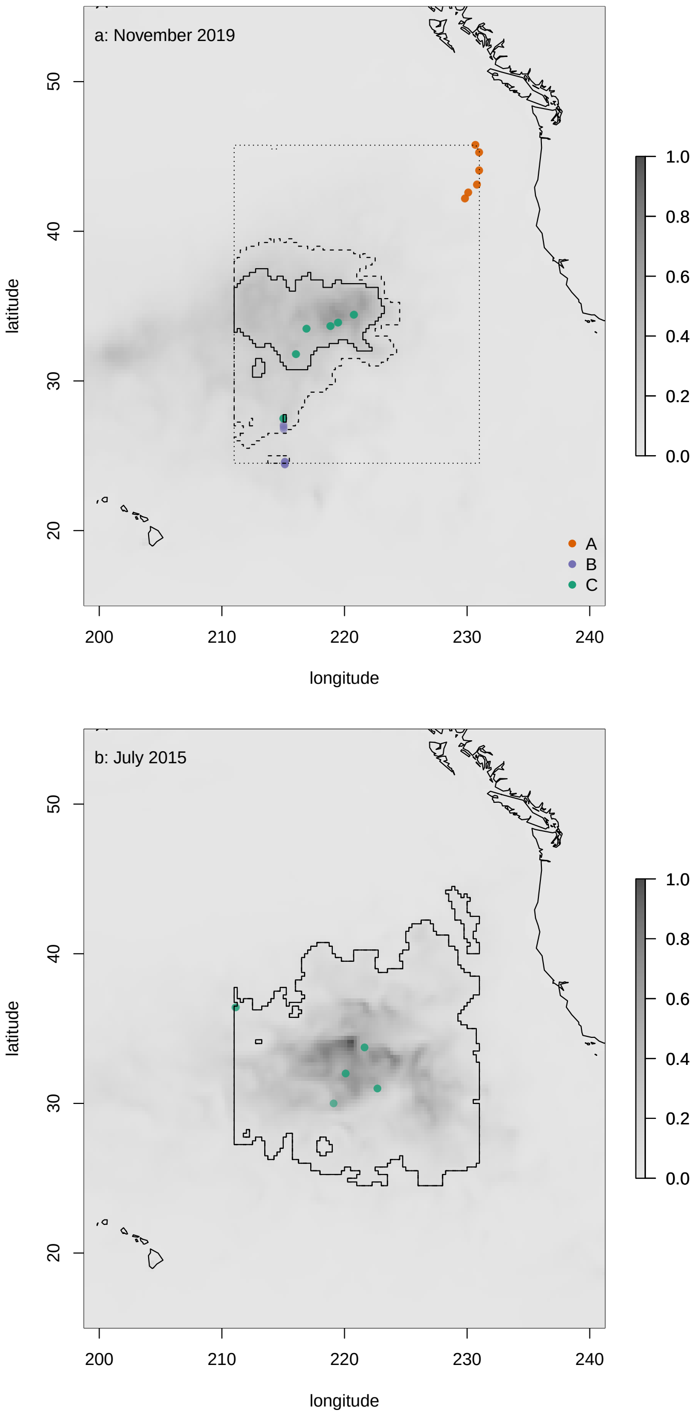

Figure E: Dimensionless tracer concentration (shading) and locations of observations in the North Pacific for the Egger et al. [6] data from (a) November 2019 and (b) July 2015. Orange points are area A, purple area B and green area C (as defined *a priori*). Dotted lines enclose the region Ω_{all} for each year, containing all points with tracer concentration as least as large as the minimum over all sites at which observations were made in any year, and within the smallest rectangle $\mathcal{R}$ of latitude and longitude, parallel with the longitude axis, that just encloses the sites at which observations were made. Dashed lines enclose the region Ω_{BC}, containing all points in $\mathcal{R}$ with tracer concentrations at least as large as the minimum over all sites at which observations were made within areas B or C in any year. Solid lines enclose the region Ω_C, containing all points in $\mathcal{R}$ with tracer concentrations at least as large as the minimum over all sites at which observations were made within area C in any year. Note that in 2015, the only observations were in area C, so these three regions coincide. The data underlying this Figure can be found in S1 Data. Maps created in R using the maps package (`https://cran.r-project.org/package=maps`) and Natural Earth data (`https://www.naturalearthdata.com/`).

We used similar prior choices to those given in section F. For all the parameters also appearing in section F, we used the prior choices given there. For the patch effects $\boldsymbol{\beta}_B$ and $\boldsymbol{\beta}_C$ we used independent $N(0, 4)$ priors, as for the patch effect β_2 in section F. For the interaction effects $\boldsymbol{\beta}_{1,B}$ and $\boldsymbol{\beta}_{1,C}$, we used independent $N(0, 1)$ priors, as for the interaction effect β_3 in section F.

Estimation was as in section G, except that we ran for 4000 warmup and 4000 sampling iterations to get sufficient effective sample size for parameters associated with the elements of $\boldsymbol{\Sigma}$. This took approximately 1.5 h on an Ubuntu 20.04.4 system with 4 Intel Core i7-4600M 2.9 GHz cores and 15.3 GiB RAM. Effective sample size was 887 for one of the parameters associated with $\boldsymbol{\Sigma}$, but greater than 1000 for all others. The $\hat{R}$ statistic was no larger than 1.0042 for all parameters. Inspection of trace plots did not suggest any obvious problems with convergence.

We visualized results using a similar approach to that taken for the Vortex Swim data. We produced plots of the relationship between log density and log tracer concentration as in Section H, but with three regions A, B and C instead of inside- and outside-patch regions. We plotted posterior distributions of tracer effects for these three regions: $\boldsymbol{\beta}_1$ in region A, $\boldsymbol{\beta}_1 + \boldsymbol{\beta}_{1B}$ in region B, and $\boldsymbol{\beta}_1 + \boldsymbol{\beta}_{1C}$ in region C. We plotted posterior distributions of differences in expected log density between regions C and B (Δ_{CB}), and between regions B and A (Δ_{BA}).

K Results

The relationship between log density of each category of object and log tracer concentration was generally positive (Figure F, slopes), and for *Velella*, *Porpita* and *Janthina*, there was also a clear positive effect of being in the patch (Figure Fa, b and c, orange vs. green). For *Glaucus* and *Physalia*, there were many zero counts (Figure Fd and e, vertical lines on x-axis) and the posterior mean relationship fell clearly below the points with non-zero counts. This does not indicate that the model fits these observations poorly, rather that estimates of true density are reduced by observations with zero counts. Posterior distributions for all parameters are summarized in Table A.

The posterior distributions of elements of the log tracer effect outside the patch ($\boldsymbol{\beta}_1$) were mainly positive for *Porpita*, *Janthina* and plastic (Figure Gb, c, f, orange) and more likely to be positive than negative for *Velella* and *Physalia* (Figure Ga, e, orange). On physical grounds, we would have expected values between 0 (no effect) and 1 (densities proportional to tracer). Somewhat surprisingly, the posterior mode for plastic exceeded 1 (Figure Gf), and values greater than 1 were not unlikely for all categories. For *Glaucus*, negative and positive effects were about equally likely (Figure Gd, orange). Effects inside the patch ($\boldsymbol{\beta}_1 + \boldsymbol{\beta}_3$) were somewhat less likely to be positive for all categories (Figure G, green), although the posterior mode for *Janthina* exceeded 1 (Figure Gc, green). However, as noted below, there may be little information about the interaction effect $\boldsymbol{\beta}_3$ in data sets with this structure.

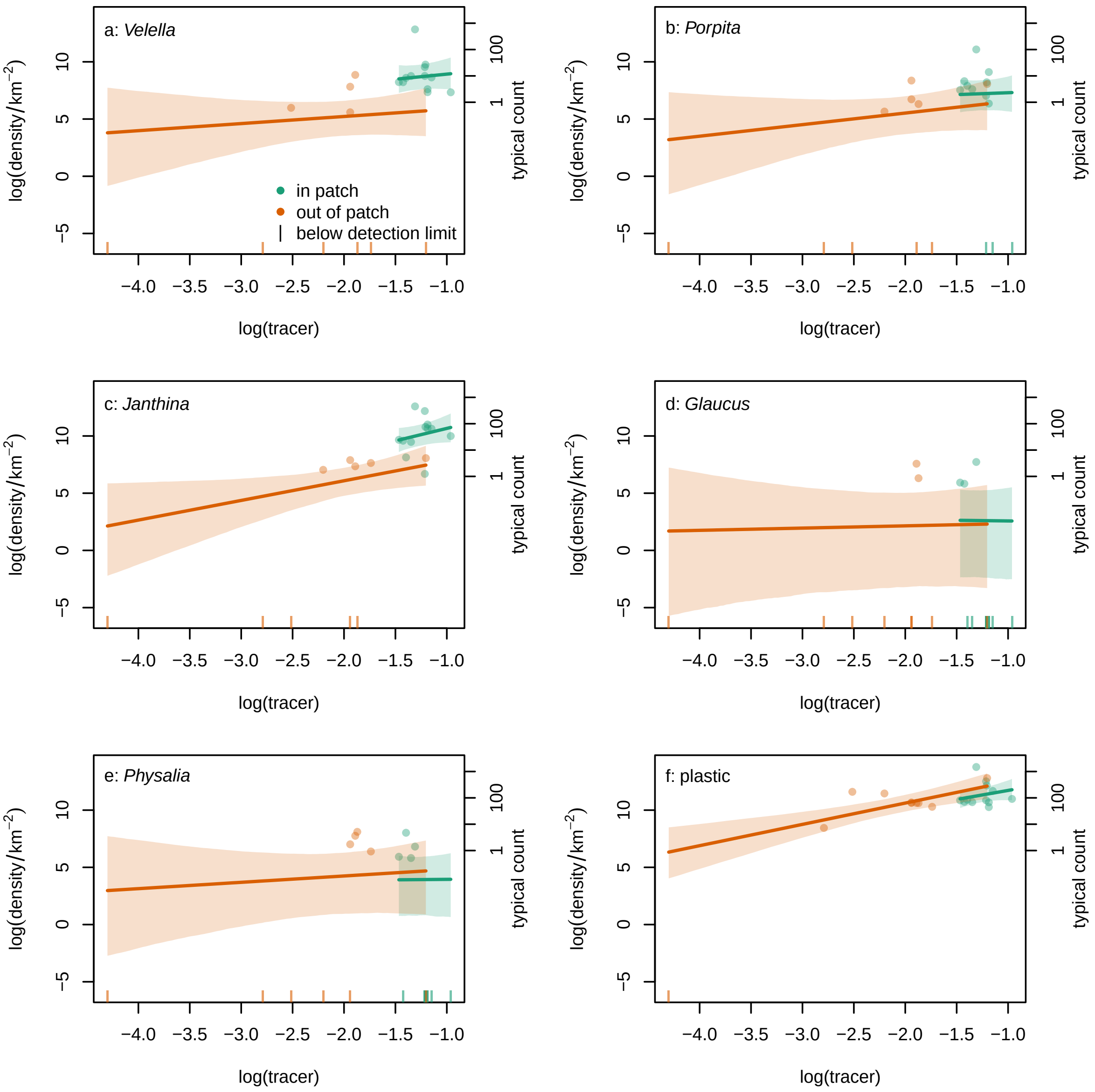

Figure F: Relationship between natural log of density (in numbers km^{-2}) and natural log of tracer concentration (relative to its maximum over July 2015, July 2019 and November 2019) for (a) *Velella*, (b) *Porpita*, (c) *Janthina*, (d) *Glaucus*, (e) *Physalia* and (f) plastic outside (orange) and in (green) the patch. Points are sample means from two independent counts, with zeros plotted as vertical lines on the x-axis (note that models were fitted to the two counts, not the mean densities). Lines are posterior means, with 95% equal-tailed credible bands, and include the detectability parameters κ_i. The right-hand y-axis has tick marks at the log densities corresponding to counts of 1, 10, 100 and 1000 objects in the mean trawled area. The data underlying this Figure can be found in S1 Data.

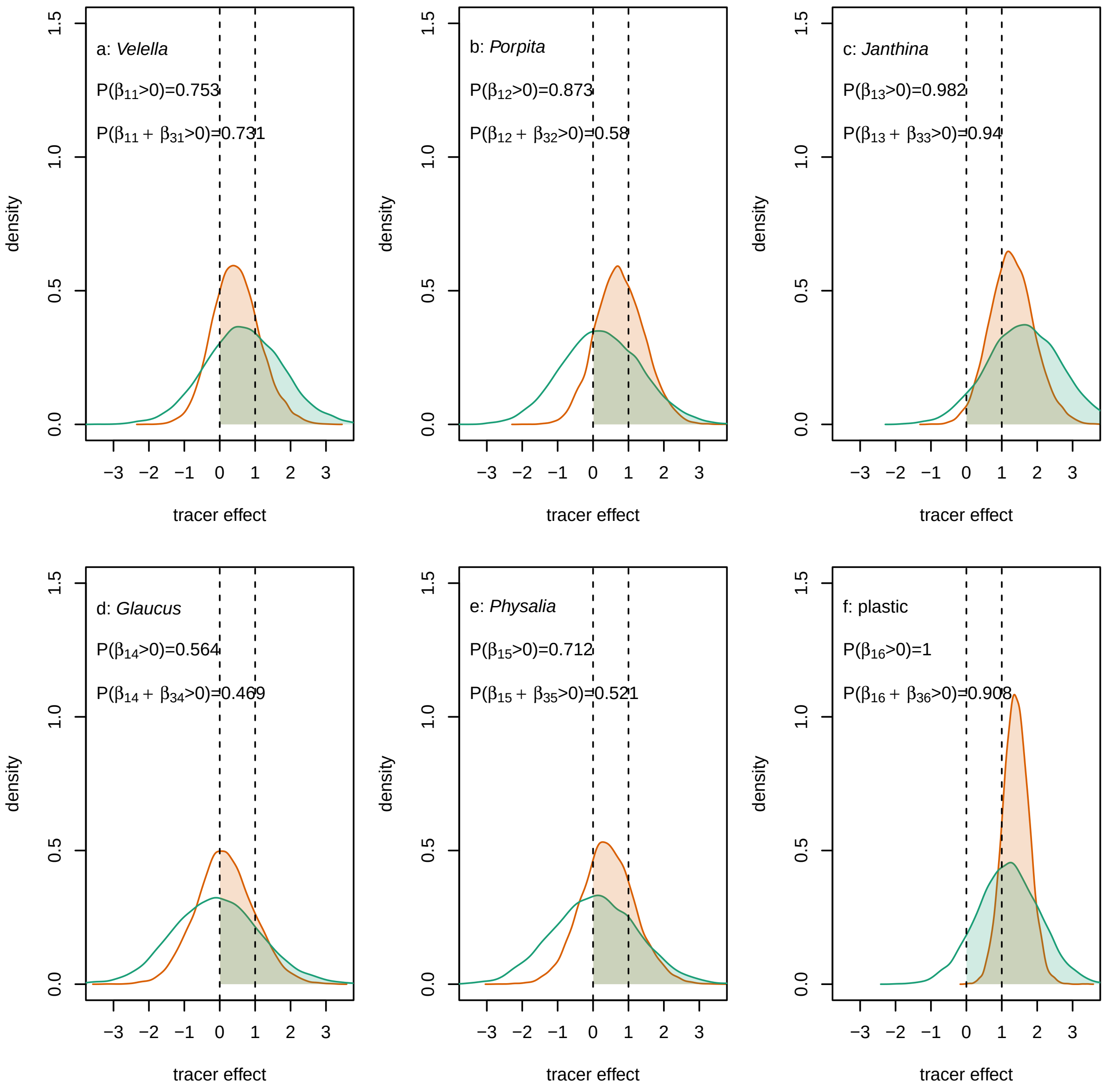

Figure G: Effect of (centered and scaled) natural log tracer concentration on expected natural log of density (in numbers km^{-2}) for (a) *Velella*, (b) *Porpita*, (c) *Janthina*, (d) *Glaucus*, (e) *Physalia* and (f) plastic outside (orange, $\beta_{1,i}$) and in (green, $\beta_{1,i} + \beta_{3,i}$) the patch. Kernel density estimates of posterior distributions, with posterior probability that the effect is positive given on each panel. Vertical dashed lines at 0 and 1, physically important values for the effect. The data underlying this Figure can be found in S1 Data.

Averaged over tracer concentrations, expected natural log of density was almost certainly higher in the inside-patch region Ω_{in} than in the outside-patch region Ω_{out} for *Velella*, *Porpita*, *Janthina* and plastic (Figure Ha, b, c, f). For the rarely-captured taxa *Glaucus* and *Physalia*, the difference between inside- and outside-patch regions was centred on zero (Figure Hd and e). However, for all taxa, the posterior distribution of differences was substantially more concentrated than the prior distribution, so there was information in the data about these differences (Figure H, solid vs. dotted lines).

The posterior distributions of marginal correlations over the entire region Ω_{all} between log plastic density and the log densities of *Velella*, *Porpita* and *Janthina* were almost entirely positive (Figure Ia to c). For the rare taxa *Glaucus* and *Physalia*, negative and positive marginal correlations with log plastic were about equally likely, and the posterior distribution was only slightly more concentrated than the prior, suggesting that there was little information in these data about correlations for these taxa (Figure Id and e, solid vs. dotted lines). The posterior distribution of the marginal correlation between log plastic density and total log neuston was almost entirely positive (Figure If). The qualitative pattern was the same for the inside-patch (Ω_{in}) and outside-patch (Ω_{out}) regions considered separately (Figures J and K).

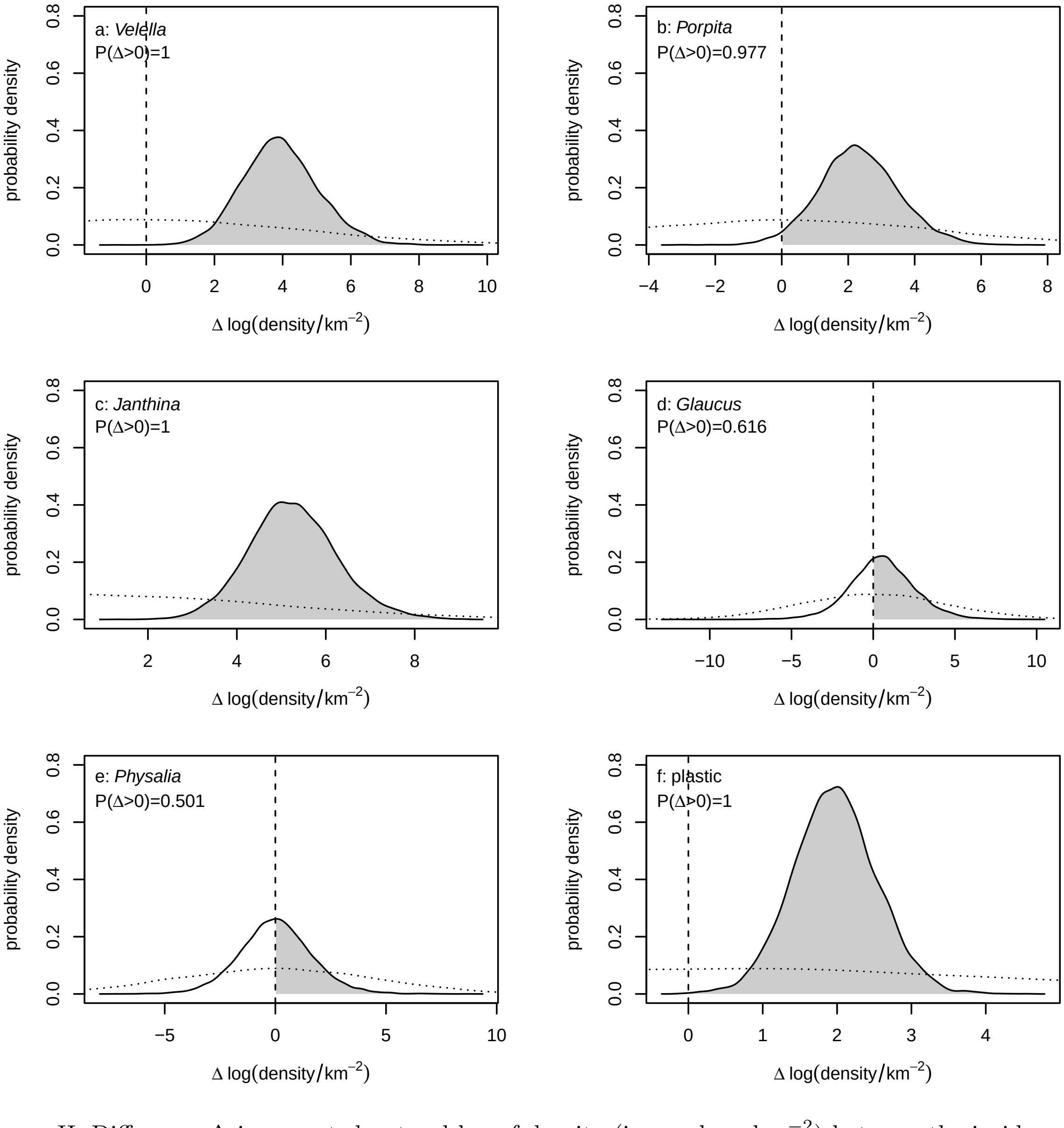

Figure H: Difference Δ in expected natural log of density (in numbers km^{-2}) between the inside-patch (Ω_{in}) and outside-patch (Ω_{out}) regions for (a) *Velella*, (b) *Porpita*, (c) *Janthina*, (d) *Glaucus*, (e) *Physalia* and (f) plastic. Kernel density estimates of posterior distributions, with posterior probability that the difference is positive given on each panel. Dotted lines are kernel density estimates of the prior distribution for each difference. The data underlying this Figure can be found in S1 Data.

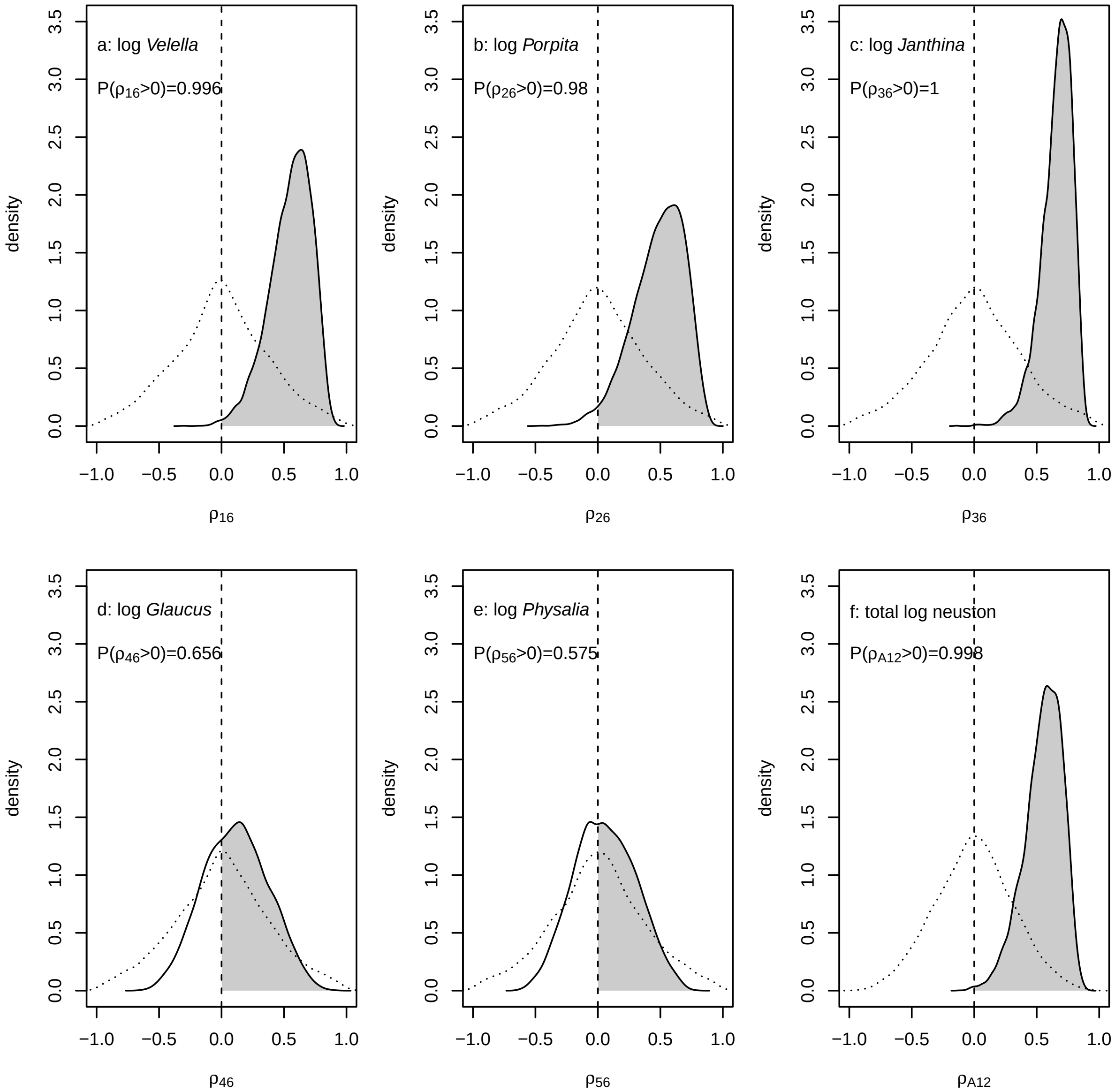

Figure I: Posterior densities of marginal correlations ρ over the entire region Ω_{all} between log plastic density and log densities of a: *Velella*, b: *Porpita*, c: *Janthina*, d: *Glaucus*, e: *Physalia* and f: total log neuston. Kernel density estimates, with vertical dashed lines at zero. Posterior probability that each marginal correlation is positive is indicated. Dotted lines are kernel density estimates of the prior distribution for each correlation. The data underlying this Figure can be found in S1 Data.

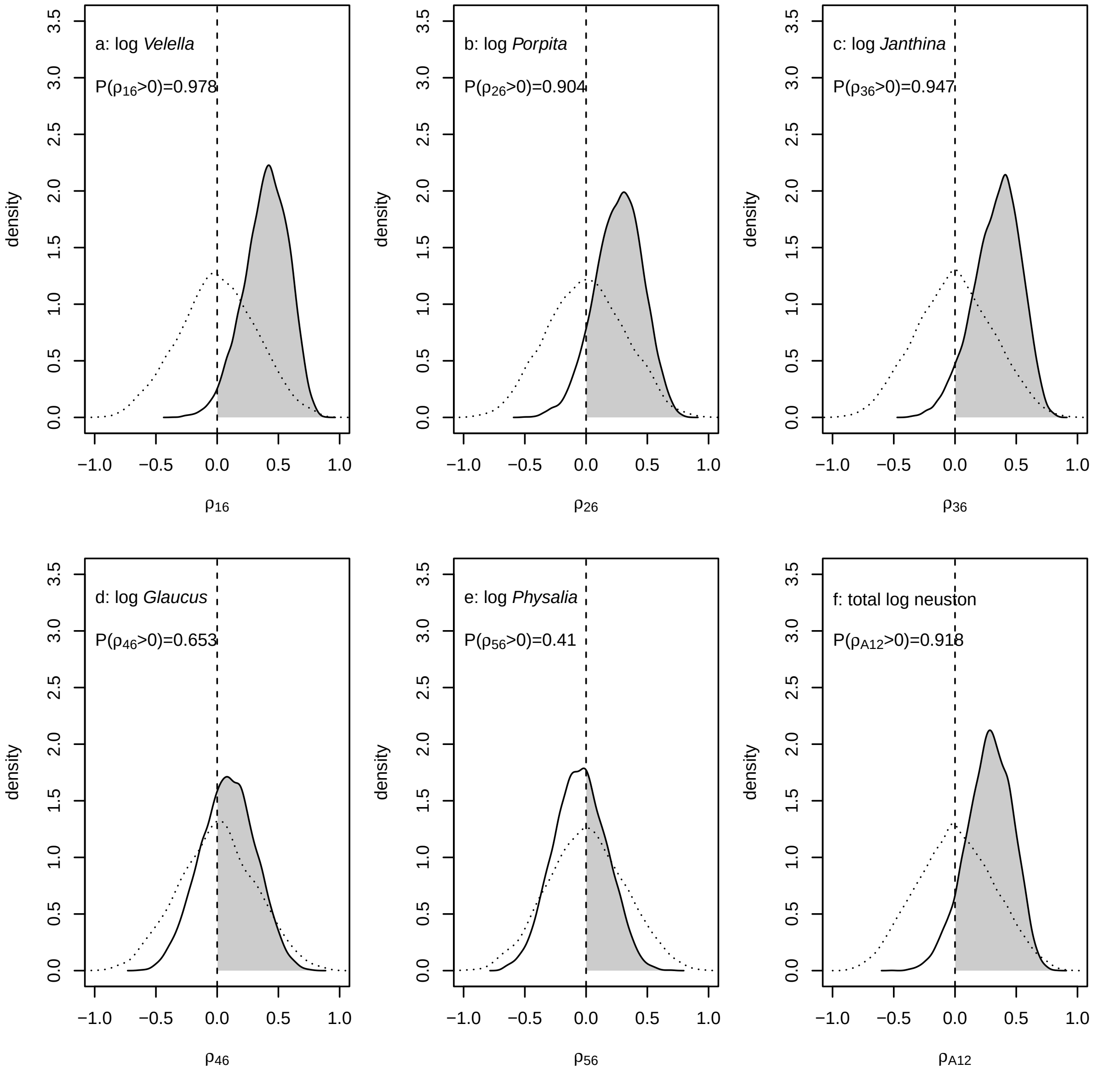

Figure J: Posterior densities of marginal correlations ρ over the inside-patch region Ω_{in} between log plastic density and log densities of a: *Velella*, b: *Porpita*, c: *Janthina*, d: *Glaucus*, e: *Physalia* and f: total log neuston. Kernel density estimates, with vertical dashed lines at zero. Posterior probability that each marginal correlation is positive is indicated. Dotted lines are kernel density estimates of the prior distribution for each correlation. The data underlying this Figure can be found in S1 Data.

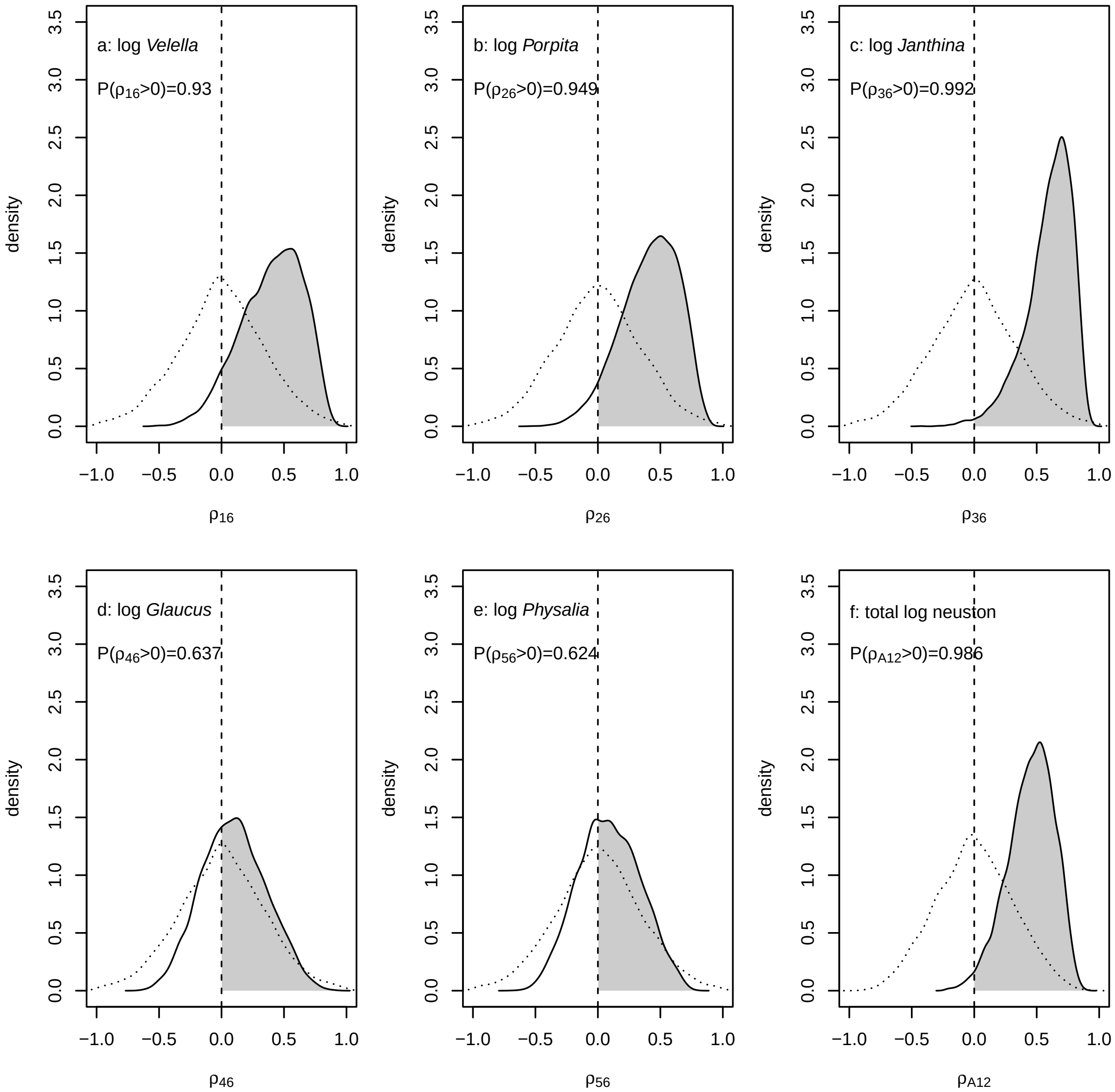

Figure K: Posterior densities of marginal correlations ρ over the outside-patch region Ω_{out} between log plastic density and log densities of a: *Velella*, b: *Porpita*, c: *Janthina*, d: *Glaucus*, e: *Physalia* and f: total log neuston. Kernel density estimates, with vertical dashed lines at zero. Posterior probability that each marginal correlation is positive is indicated. Dotted lines are kernel density estimates of the prior distribution for each correlation. The data underlying this Figure can be found in S1 Data.

There appeared to be little information in these data on absolute densities. There were negative posterior relationships between the intercepts $\beta_{0,j}$ and log detectability κ_j for each category j, particularly for the most abundant categories *Velella*, *Janthina* and plastic (Figure L). Thus it may be hard to distinguish between high absolute density with low detectability, and low absolute density with high detectability. However, this did not appear to be a structural identifiability problem. For all sites and categories, the Hessian was of full rank, suggesting that the parameters may be identifiable [4]. Note that the main results of interest, including relationships with log tracer density, differences in log density between inside and outside the patch, and marginal correlations between log densities of neuston and plastic, do not require knowledge of absolute densities.

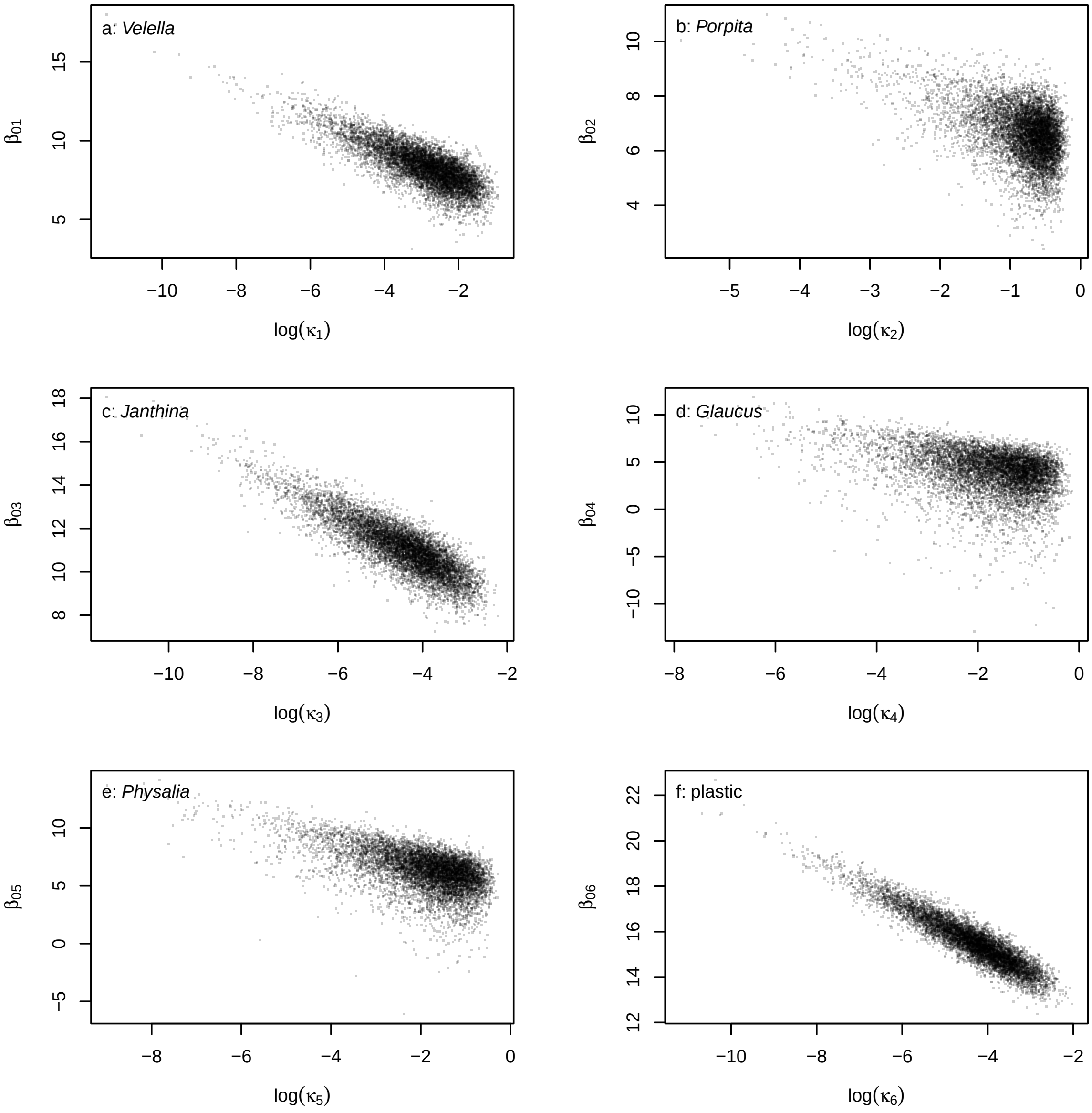

Figure L: Posterior relationships between intercept $\beta_{0,i}$ and log detectability κ_i for a: *Velella*, b: *Porpita*, c: *Janthina*, d: *Glaucus*, e: *Physalia* and f: plastic. The data underlying this Figure can be found in S1 Data.

Fitting to simulated data sets did not suggest any major errors in coding (Figures M, N). In most cases, posterior densities (grey lines) were concentrated around the true values (pink lines), were more concentrated than the priors (dashed lines), and the 95% highest posterior density regions contained the true values between 8 and 10 times out of 10. However, for the intercept $\boldsymbol{\beta}_0$, there was evidence of bias, with 95% highest posterior density regions containing the true values as little as 5 times out of 10 (Figure Mc and f). In addition, posterior densities of detectabilities $\boldsymbol{\kappa}$ were not concentrated around the true values for many simulated data sets (Figure My to ad). As noted above, this is likely to be a consequence of the strong negative posterior relationships between elements of $\boldsymbol{\beta}_0$ and $\log \boldsymbol{\kappa}$, and will not affect the main results of interest. Also, prior and posterior densities were almost identical for the interaction parameter $\boldsymbol{\beta}_3$ (Figure Ms to x), suggesting that there is likely to be very little information on differences in the slope of the relationship between log densities and log tracer inside and outside the patch.

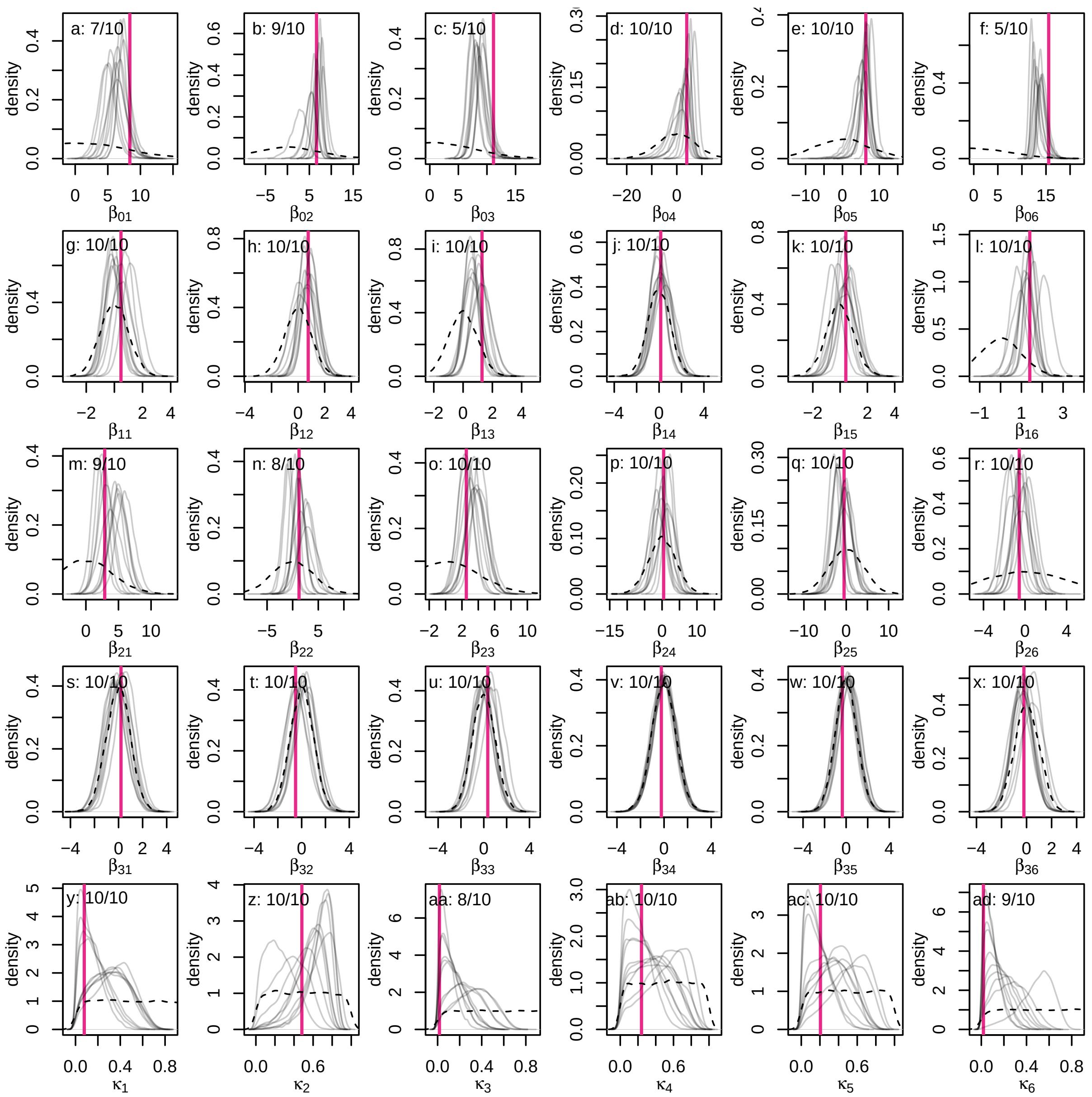

Figure M: Posterior densities for elements of the parameters $\boldsymbol{\beta}_0$, $\boldsymbol{\beta}_1$, $\boldsymbol{\beta}_2$, $\boldsymbol{\beta}_3$ and $\boldsymbol{\kappa}$ (grey lines, kernel density estimates) from 10 simulated data sets for which the true values were the posterior means estimated from the real data set (vertical pink lines). Dashed lines are kernel density estimates of the priors from a sample of the same size as from the posteriors. The proportion of simulated data sets for which the 95% highest posterior density region contained the true parameter value is indicated on each panel. The data underlying this Figure can be found in S1 Data.

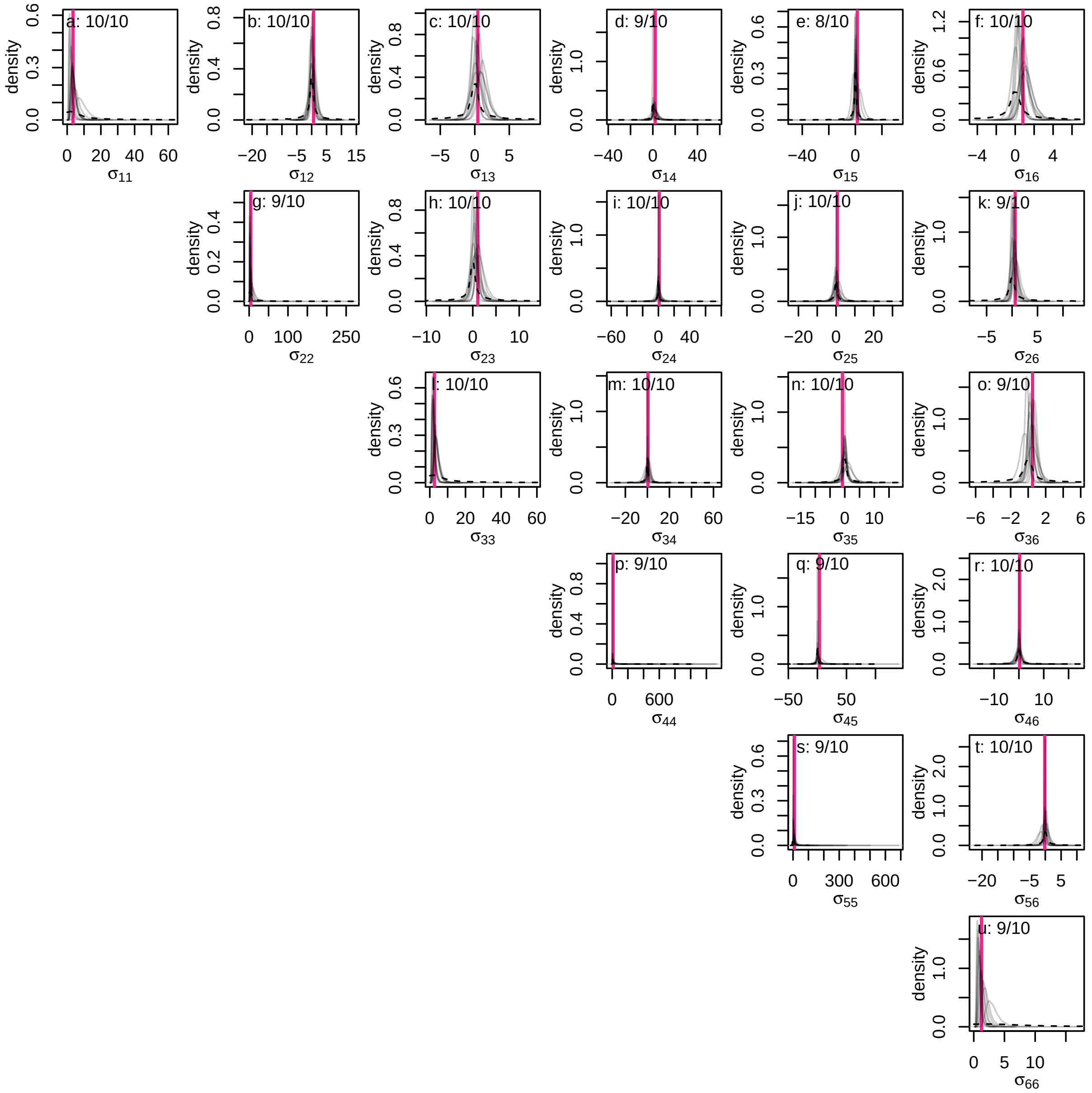

Figure N: Posterior densities for elements of the upper triangle of the covariance matrix $\mathbf{\Sigma}$ of observation-level random effects (grey lines, kernel density estimates) from 10 simulated data sets for which the true values were the posterior means estimated from the real data set (vertical pink lines). Dashed lines are kernel density estimates of the priors from a sample of the same size as from the posteriors. The proportion of simulated data sets for which the 95% highest posterior density region contained the true parameter value is indicated on each panel. The data underlying this Figure can be found in S1 Data.

Graphical posterior predictive checks did not suggest any major problems with the model. For each category of object, simulated relationships between log density and log tracer (Figure O, open circles, with zero mean counts represented by vertical lines just above the x axis) did not appear to differ systematically from the observed relationships (Figure O, filled circles, with zero mean counts represented by vertical lines on the x-axis). Note that where the observed mean count was zero (represented by a vertical line just above the x-axis), small simulated non-zero counts are plausible but correspond to much higher densities, and will be represented by points far above the x-axis. Similarly, where the observed mean count was non-zero but small, zero simulated mean counts are plausible, and will be represented by vertical lines just above the x-axis. Thus, the empty horizontal band in the middle of each panel on Figure O is entirely expected. For each category of object, the posterior predictive distribution of correlations between the two counts over observations was strongly skewed towards values close to 1 (Figure P, histograms), and for all categories other than *Glaucus* and *Physalia*, the observed correlation (Figure P, vertical dashed lines) was very close to 1. For the rare taxa *Glaucus* and *Physalia* (Figure Pd and e), the observed correlation was positive but much weaker than for other taxa, and the posterior predictive distribution of correlations was less strongly skewed towards 1 than for other taxa, and tended to be higher than the observed correlation. This suggests the possibility of additional sources of observation error not captured by our model. Nevertheless, our model appears to capture the main features of the observations.

Leave-one-out cross-validation estimates of log predictive density suggested that the observation `SJR_019` was very unlikely under a model fitted to the other observations (Figure Q). This was an in-patch observation with high counts of *Velella*, *Porpita*, *Janthina* and plastic. To check that this observation was not having a substantial effect, we recalculated the main results with this observation omitted. We confirmed that the posterior estimates of the relationship between log density and log tracer concentration (Figure R), the differences in expected natural log density between inside-patch and outside-patch regions (Figure S), and the marginal correlations between log plastic density and log density of each taxon (Figure T), were not substantially different without `SJR_019`.

We also noted that one observation coded *a priori* as outside the patch on geographical grounds in fact had a higher tracer concentration than the minimum for observations coded *a priori* as inside the patch (Figure D, orange point in top right of region bounded by solid line). We re-ran the model with this observation recoded as inside the patch. The main results were not substantially different (Figures U, V, W).

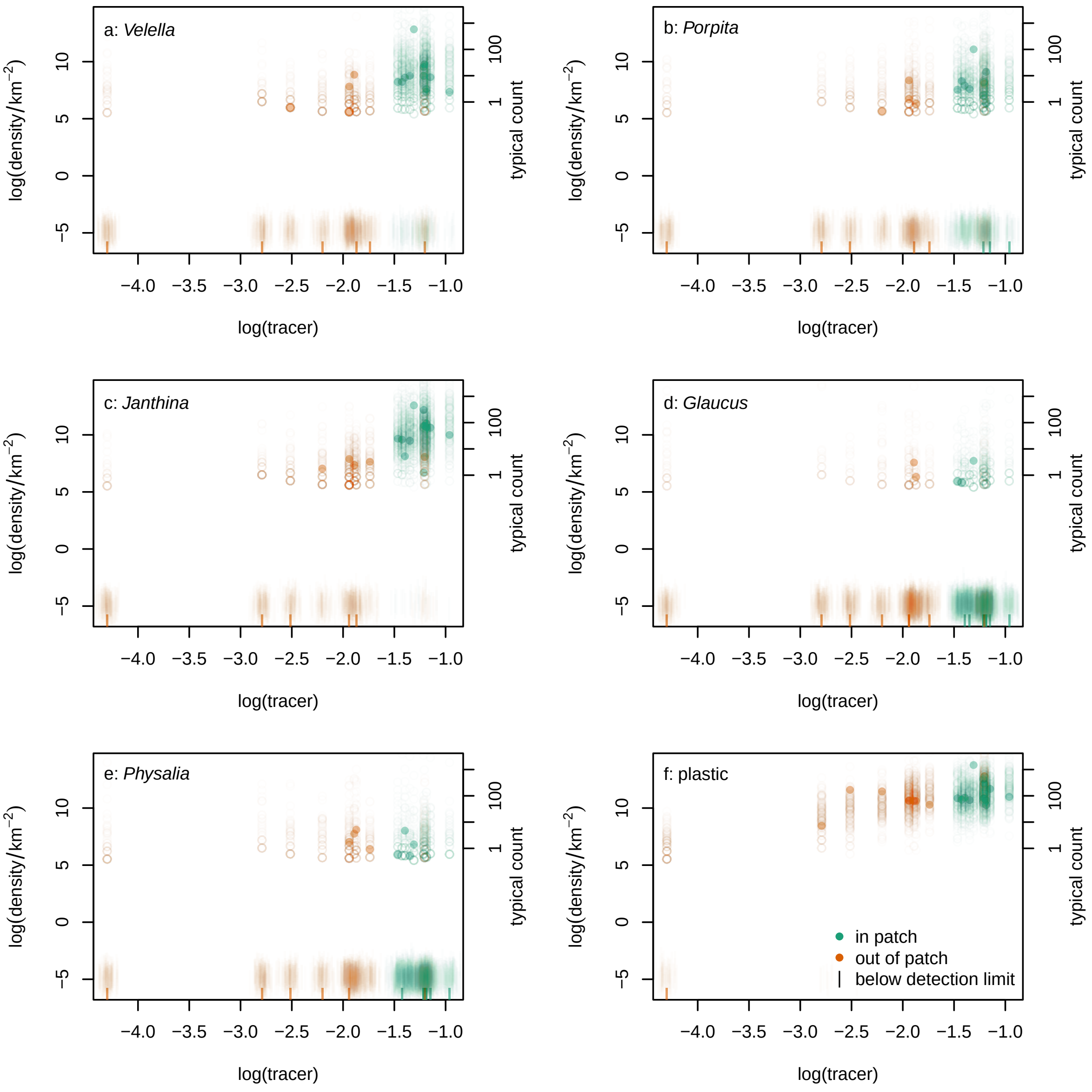

Figure O: Posterior predictive relationship between natural log of density (in numbers km^{-2}) and natural log of dimensionless tracer concentration for (a) *Velella*, (b) *Porpita*, (c) *Janthina*, (d) *Glaucus*, (e) *Physalia* and (f) plastic outside (orange) and in (green) the patch. Filled points are observed sample means from two independent counts, with zeros plotted as vertical lines on the x-axis Open points are sample means from two independent simulated counts, for each of 200 simulated data sets, with zeros plotted as vertical lines just above the x-axis (with jittered x-coordinates). The right-hand y-axis has tick marks at the log densities corresponding to counts of 1, 10, 100 and 1000 objects in the mean trawled area. The data underlying this Figure can be found in S1 Data.

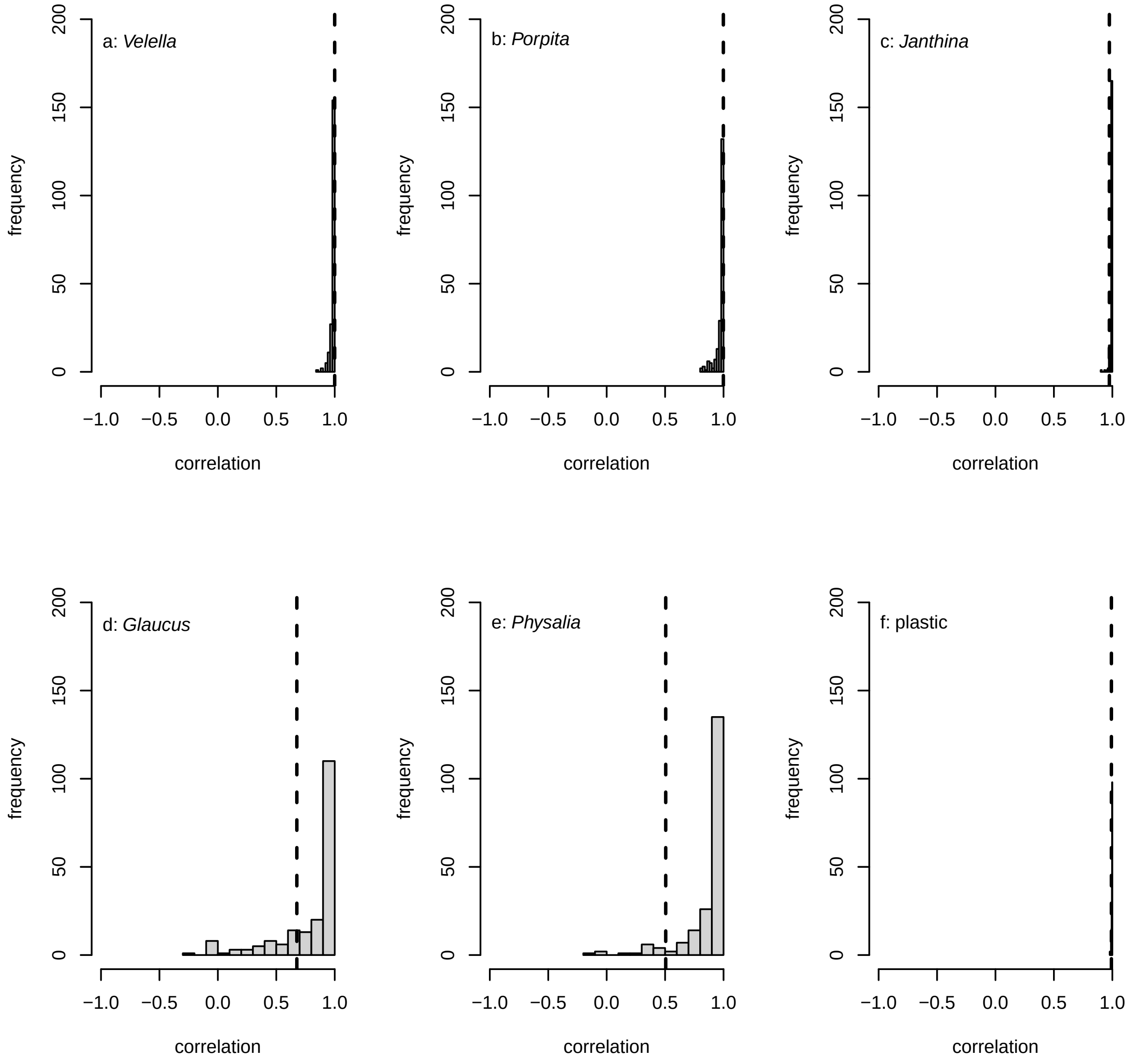

Figure P: Posterior predictive distributions of the correlation between the two counts for (a) *Velella*, (b) *Porpita*, (c) *Janthina*, (d) *Glaucus*, (e) *Physalia* and (f) plastic, from 200 simulated data sets. Vertical dashed lines: observed correlations. The data underlying this Figure can be found in S1 Data.

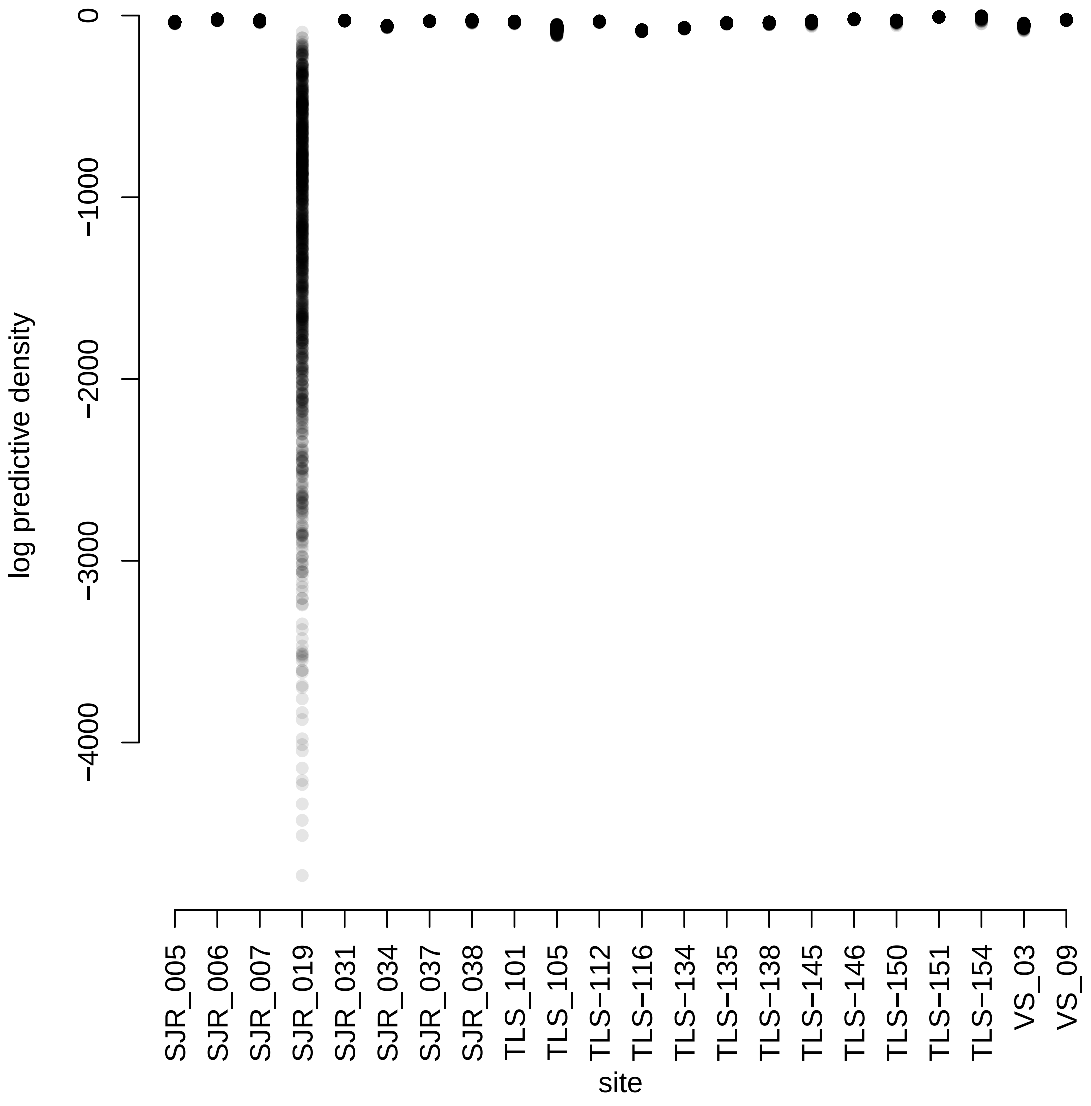

Figure Q: Leave-one-out cross-validation estimates of log predictive density for each site. Points are log predictive densities for 1000 sets of parameters drawn from the posterior density estimated without the focal site. For each point, the log predictive density is integrated over the distribution of the observation-level random effect ε, by classical Monte Carlo integration with a sample of size 1×10^5. The data underlying this Figure can be found in S1 Data.

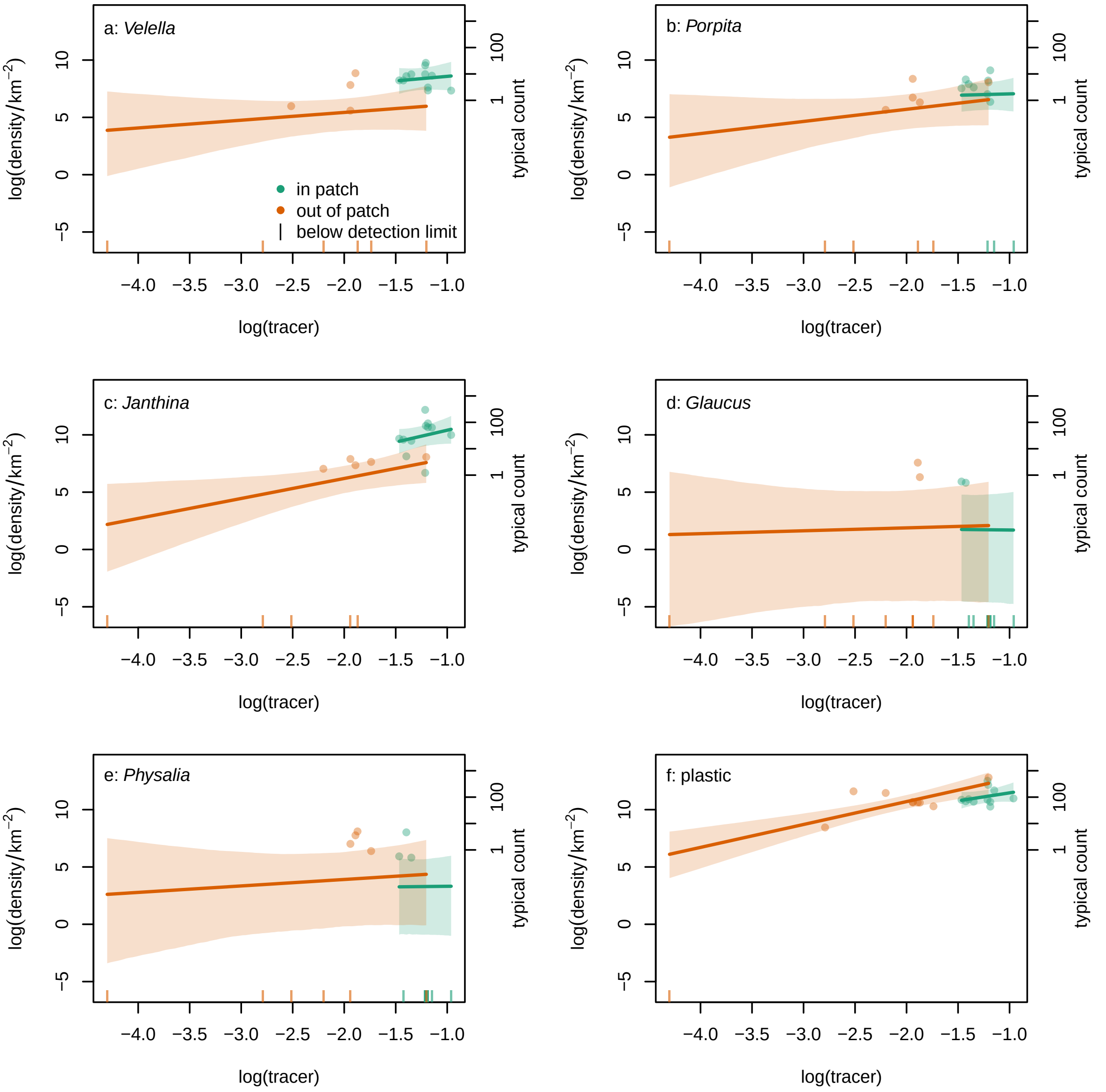

Figure R: Relationship between natural log of density (in numbers km^{-2}) and natural log of dimensionless tracer concentration, with the unusual observation `SJR_019` omitted, for (a) *Velella*, (b) *Porpita*, (c) *Janthina*, (d) *Glaucus*, (e) *Physalia* and (f) plastic outside (orange) and in (green) the patch. Points are sample means from two independent counts, with zeros plotted as vertical lines on the x-axis (note that models were fitted to the two counts, not the mean densities). Lines are posterior means, with 95% equal-tailed credible bands, and include the detectability parameters κ_i. The right-hand y-axis has tick marks at the log densities corresponding to counts of 1, 10, 100 and 1000 objects in the mean trawled area. The data underlying this Figure can be found in S1 Data.

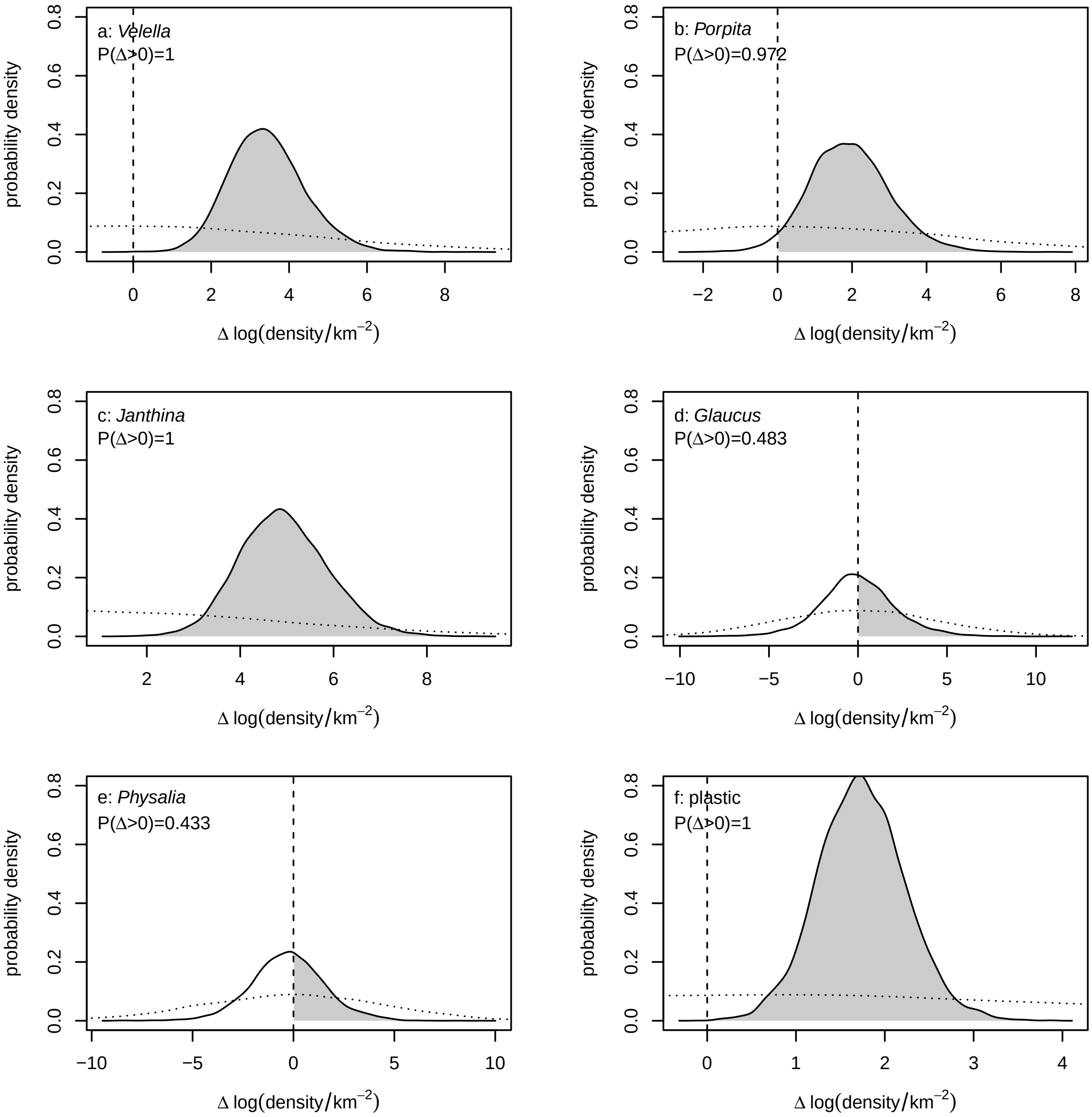

Figure S: Difference Δ in expected natural log of density (in numbers km^{-2}) between the inside-patch (Ω_{in}) and outside-patch (Ω_{out}) regions, with the unusual observation SJR_019 omitted, for (a) *Velella*, (b) *Porpita*, (c) *Janthina*, (d) *Glaucus*, (e) *Physalia* and (f) plastic. Kernel density estimates of posterior distributions, with posterior probability that the difference is positive given on each panel. Dotted lines are kernel density estimates of the prior distribution for each difference. The data underlying this Figure can be found in S1 Data.

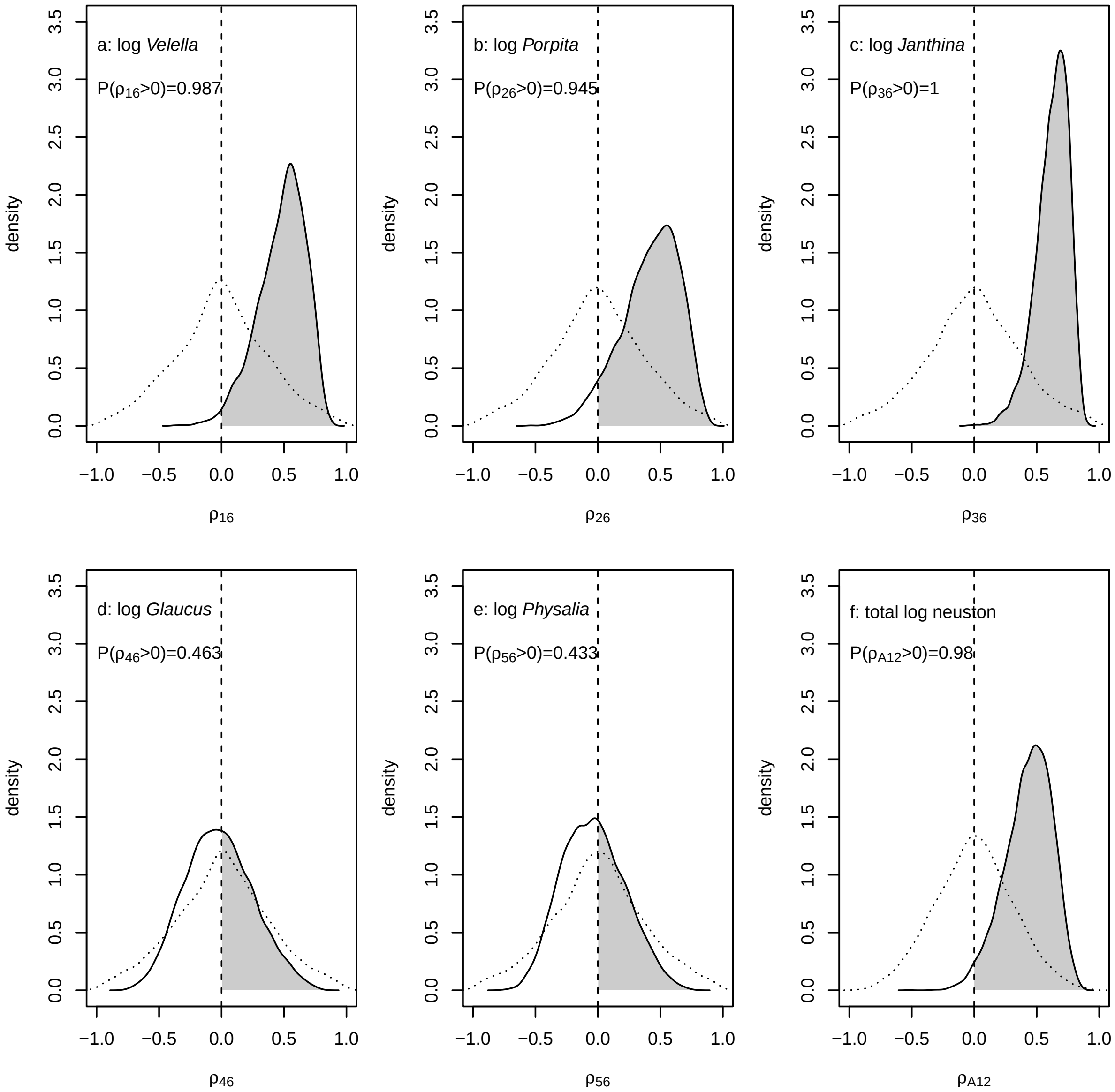

Figure T: Posterior densities of marginal correlations ρ over the entire region Ω_{all} between log plastic density and log densities, with the unusual observation SJR_019 omitted, of a: *Velella*, b: *Porpita*, c: *Janthina*, d: *Glaucus*, e: *Physalia* and f: total log neuston. Kernel density estimates, with vertical dashed lines at zero. Posterior probability that each marginal correlation is positive is indicated. Dotted lines are kernel density estimates of the prior distribution for each correlation. The data underlying this Figure can be found in S1 Data.

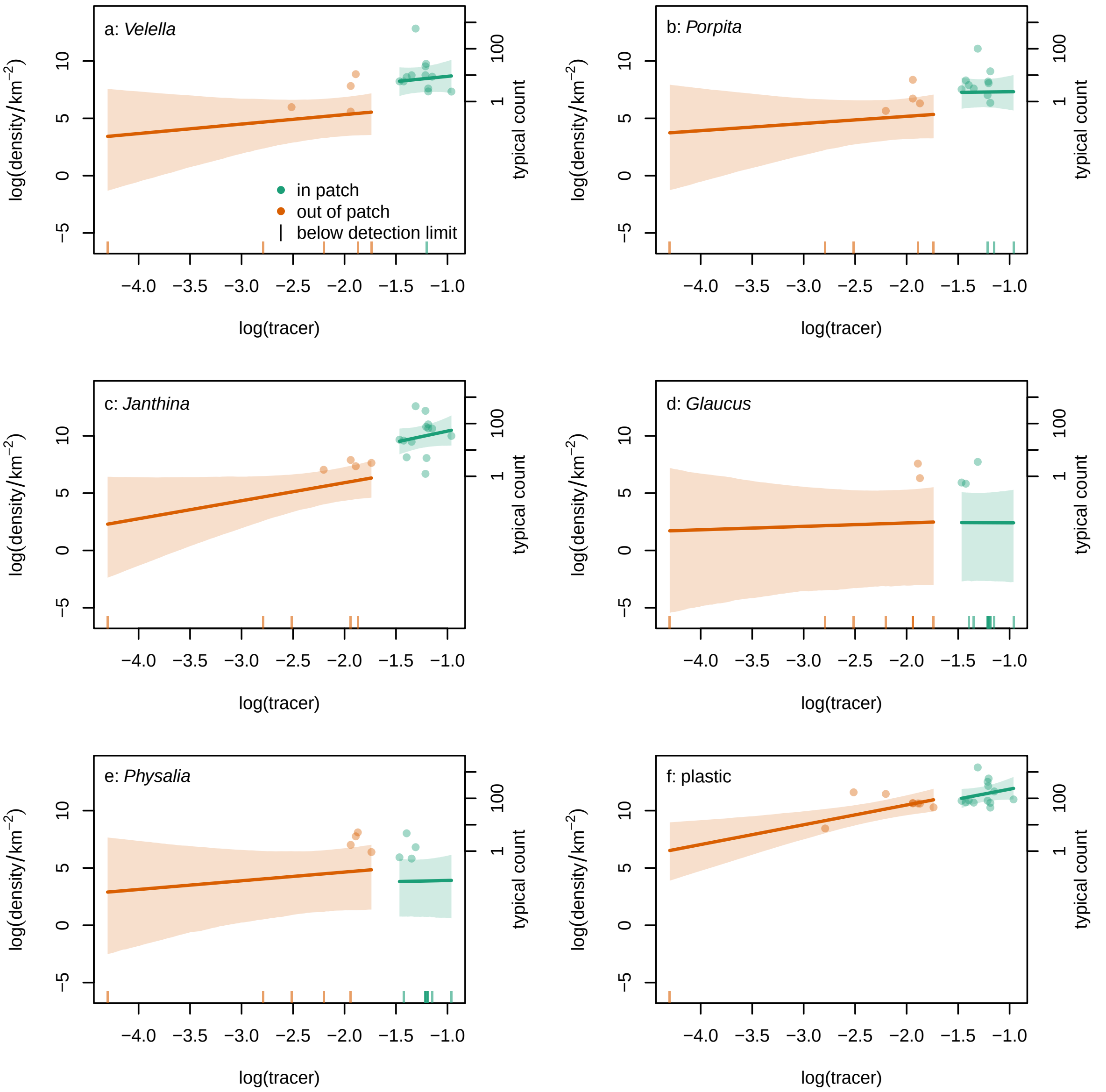

Figure U: Relationship between natural log of density (in numbers km^{-2}) and natural log of dimensionless tracer concentration, with the outside observation having higher tracer concentration than the minimum for inside observations recoded as inside, for (a) *Velella*, (b) *Porpita*, (c) *Janthina*, (d) *Glaucus*, (e) *Physalia* and (f) plastic outside (orange) and in (green) the patch. Points are sample means from two independent counts, with zeros plotted as vertical lines on the x-axis (note that models were fitted to the two counts, not the mean densities). Lines are posterior means, with 95% equal-tailed credible bands, and include the detectability parameters κ_i. The right-hand y-axis has tick marks at the log densities corresponding to counts of 1, 10, 100 and 1000 objects in the mean trawled area. The data underlying this Figure can be found in S1 Data.

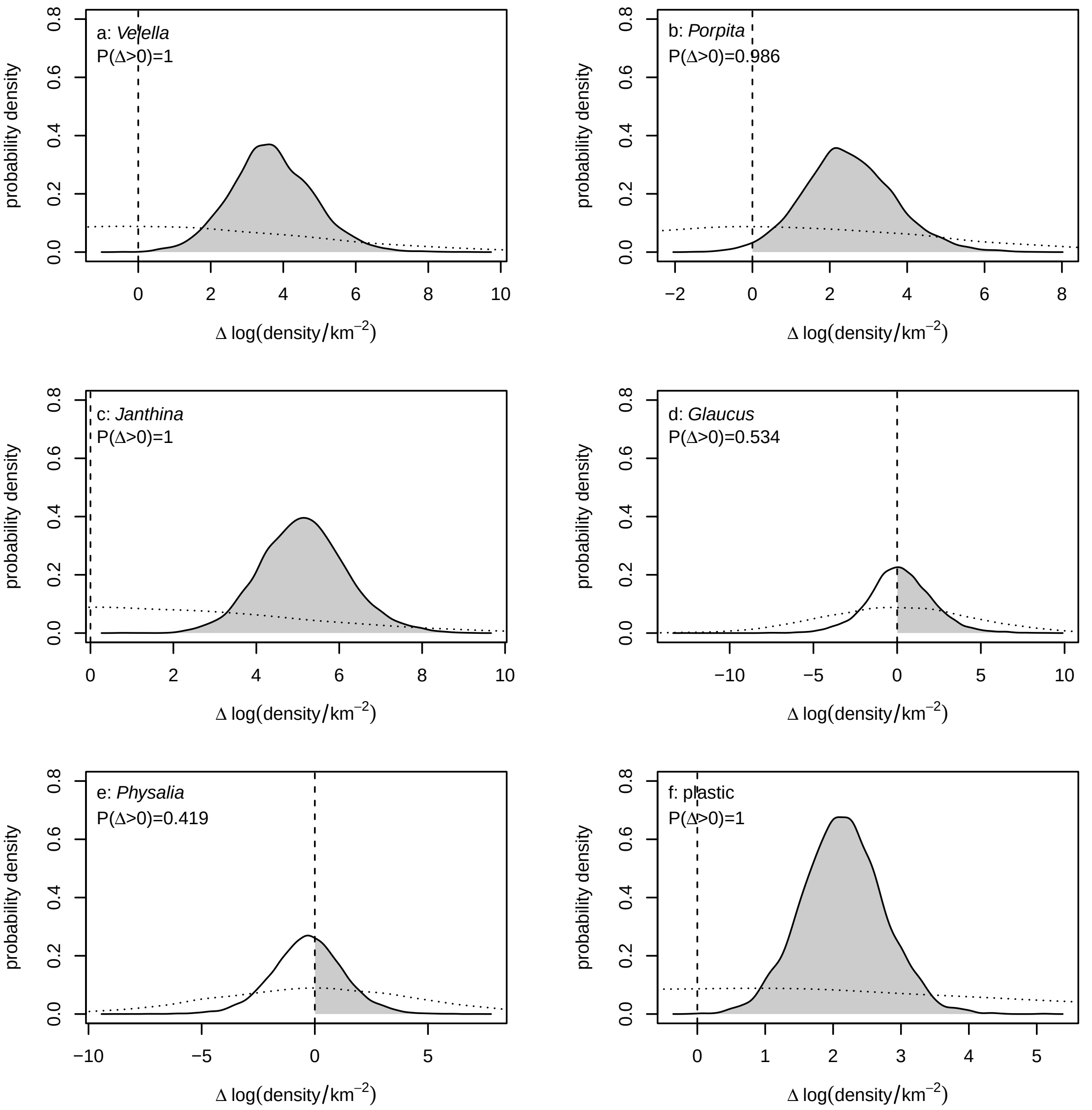

Figure V: Difference Δ in expected natural log of density (in numbers km^{-2}) between the inside-patch (Ω_{in}) and outside-patch (Ω_{out}) regions, with the outside observation having higher tracer concentration than the minimum for inside observations recoded as inside, for (a) *Velella*, (b) *Porpita*, (c) *Janthina*, (d) *Glaucus*, (e) *Physalia* and (f) plastic. Kernel density estimates of posterior distributions, with posterior probability that the difference is positive given on each panel. Dotted lines are kernel density estimates of the prior distribution for each difference. The data underlying this Figure can be found in S1 Data.

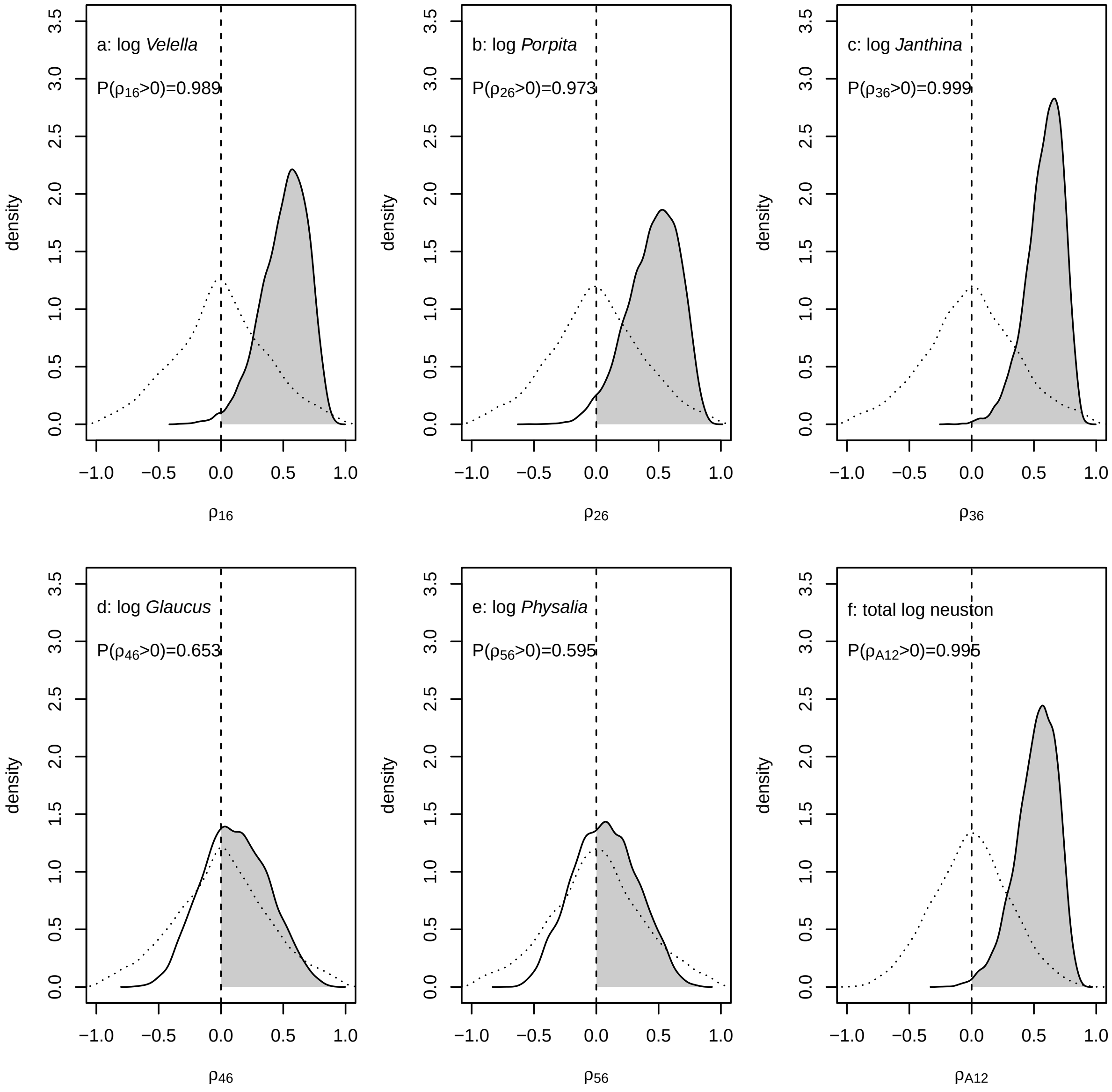

Figure W: Posterior densities of marginal correlations ρ over the entire region Ω_{all} between log plastic density and log densities, with the outside observation having higher tracer concentration than the minimum for inside observations recoded as inside, of a: *Velella*, b: *Porpita*, c: *Janthina*, d: *Glaucus*, e: *Physalia* and f: total log neuston. Kernel density estimates, with vertical dashed lines at zero. Posterior probability that each marginal correlation is positive is indicated. Dotted lines are kernel density estimates of the prior distribution for each correlation. The data underlying this Figure can be found in S1 Data.

Table A: Parameter estimates for the model defined by Equation D: intercept $\boldsymbol{\beta}_0$, tracer effect $\boldsymbol{\beta}_1$, patch effect $\boldsymbol{\beta}_2$, interaction effect $\boldsymbol{\beta}_3$, detectability $\boldsymbol{\kappa}$, rows of lower triangle of covariance matrix $\boldsymbol{\Sigma}$ of observation-level random effects. Columns are taxa and plastic. Each cell contains the posterior mean, with marginal 95 % credible highest density regions in parentheses. For plastic detectability κ_6, the highest density region consists of multiple disjoint intervals. Negative lower bounds for highest density regions for elements of $\boldsymbol{\kappa}$ are smoothing artefacts.

	Velella	*Porpita*	*Janthina*	*Glaucus*	*Physalia*	plastic
$\boldsymbol{\beta}_0$	8.38 (5.69, 10.97)	6.64 (4.61, 8.66)	11.14 (8.68, 13.59)	3.97 (-0.58, 8.41)	6.35 (2.89, 9.82)	15.56 (13.42, 17.69)
$\boldsymbol{\beta}_1$	0.47 (-0.88, 1.79)	0.76 (-0.58, 2.10)	1.29 (0.05, 2.53)	0.15 (-1.46, 1.77)	0.42 (-1.11, 1.97)	1.40 (0.67, 2.13)
$\boldsymbol{\beta}_2$	2.89 (0.52, 5.23)	1.24 (-1.20, 3.68)	2.54 (0.53, 4.58)	0.45 (-3.26, 4.18)	-0.52 (-3.80, 2.73)	-0.56 (-2.06, 0.94)
$\boldsymbol{\beta}_3$	0.20 (-1.66, 2.07)	-0.51 (-2.37, 1.35)	0.35 (-1.49, 2.17)	-0.23 (-2.13, 1.69)	-0.36 (-2.28, 1.56)	-0.21 (-1.92, 1.49)
$\boldsymbol{\kappa}$	0.08 (-0.00, 0.21)	0.48 (0.16, 0.79)	0.02 (-0.00, 0.05)	0.24 (-0.03, 0.55)	0.21 (-0.01, 0.48)	0.02 (-0.00, 0.05) (0.05, 0.05)
$\boldsymbol{\sigma}_1$	3.47 (0.95, 6.34)					
$\boldsymbol{\sigma}_2$	0.64 (-1.13, 2.47)	4.30 (0.79, 8.90)				
$\boldsymbol{\sigma}_3$	0.44 (-0.98, 1.83)	1.07 (-0.58, 2.82)	2.59 (0.57, 4.81)			
$\boldsymbol{\sigma}_4$	2.09 (-0.87, 5.65)	1.11 (-2.66, 5.27)	0.54 (-2.38, 3.48)	12.05 (-1.48, 36.21)		
$\boldsymbol{\sigma}_5$	1.49 (-0.88, 4.25)	0.71 (-2.26, 4.10)	-0.79 (-3.06, 1.51)	3.51 (-2.01, 10.00)	8.60 (-0.58, 22.03)	
$\boldsymbol{\sigma}_6$	0.81 (-0.11, 1.79)	0.61 (-0.42, 1.72)	0.51 (-0.31, 1.36)	0.28 (-1.67, 2.27)	-0.18 (-1.75, 1.41)	1.26 (0.48, 2.15)

L Results for Egger et al. [6] data

There were clear differences in density between areas in the Egger et al. [6] data (Figure X). In particular, area A appeared to have more *Velella* but less *Porpita*, *Janthina* and plastic than area C. However, the median count was zero for every taxon in these data, and it is likely that this contributes to the lack of information on many quantities of interest, outlined below. Note that the regression lines in Figure X generally lie below the points corresponding to non-zero counts because the lines are pulled down by zero counts, not because the model is failing.

Tracer effects (Figure Y) appeared weaker than for the Vortex Swim data, and were centred close to zero for *Velella* (Figure Ya) and *Physalia* (Figure Ye), and for plastic except in area B (where they were centred between 0 and 1, but uncertain: Figure Yf). For other taxa, tracer effects were centred between 0 and 1, and were uncertain, but more likely to be positive than negative.

Averaged over tracer concentrations, area C had more *Velella*, *Porpita* and plastic than area B, and less *Janthina*, *Glaucus* and *Physalia* (Figure Z). Area B had more *Janthina*, *Glaucus*, *Physalia* and plastic than area A, and less *Velella* (Figure AA).

Marginal correlations between log neuston densities and log plastic density across Ω_{all} were clearly negative for *Velella* (Figure ABa) and positive for *Janthina* (Figure ABc). For other taxa and total log neuston, there was little information in the data on these marginal correlations (Figure ABb, d, e and f: posterior densities are not clearly different from priors). The within-region marginal correlations Ω_C, Ω_B and Ω_A were similar for each region (Figures AC, AD and AE). These were weakly positive for *Velella* (panel a in each figure) and weakly negative for *Janthina* (panel c in each figure). Note that these signs were opposite to those across the entire region Ω_{all}. For other taxa, there was little information in the data, and posterior densities were not clearly different to priors.

Posterior distributions of all parameters summarized in Table B.

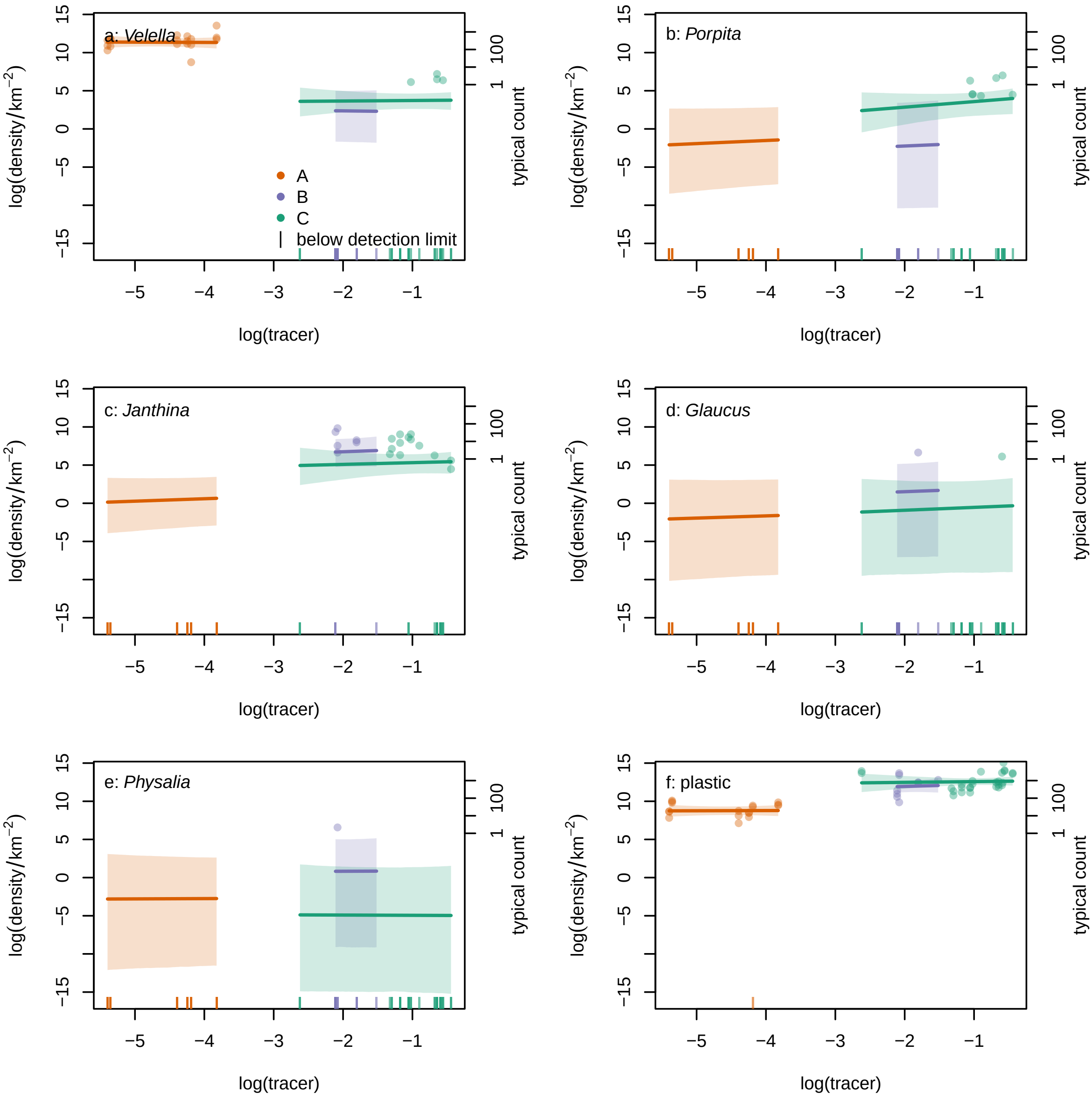

Figure X: Relationship between natural log of density (in numbers km^{-2}) and natural log of dimensionless tracer concentration in the Egger et al. [6] data for (a) *Velella*, (b) *Porpita*, (c) *Janthina*, (d) *Glaucus*, (e) *Physalia* and (f) plastic in areas A (orange), B (purple) and C (green). Points are sample estimates of density, with zeros plotted as vertical lines on the x-axis (note that models were fitted to the counts, not the densities). Lines are posterior means, with 95% equal-tailed credible bands. The right-hand y-axis has tick marks at the log densities corresponding to counts of 1, 10, 100 and 1000 objects in the mean trawled area. The data underlying this Figure can be found in S1 Data.

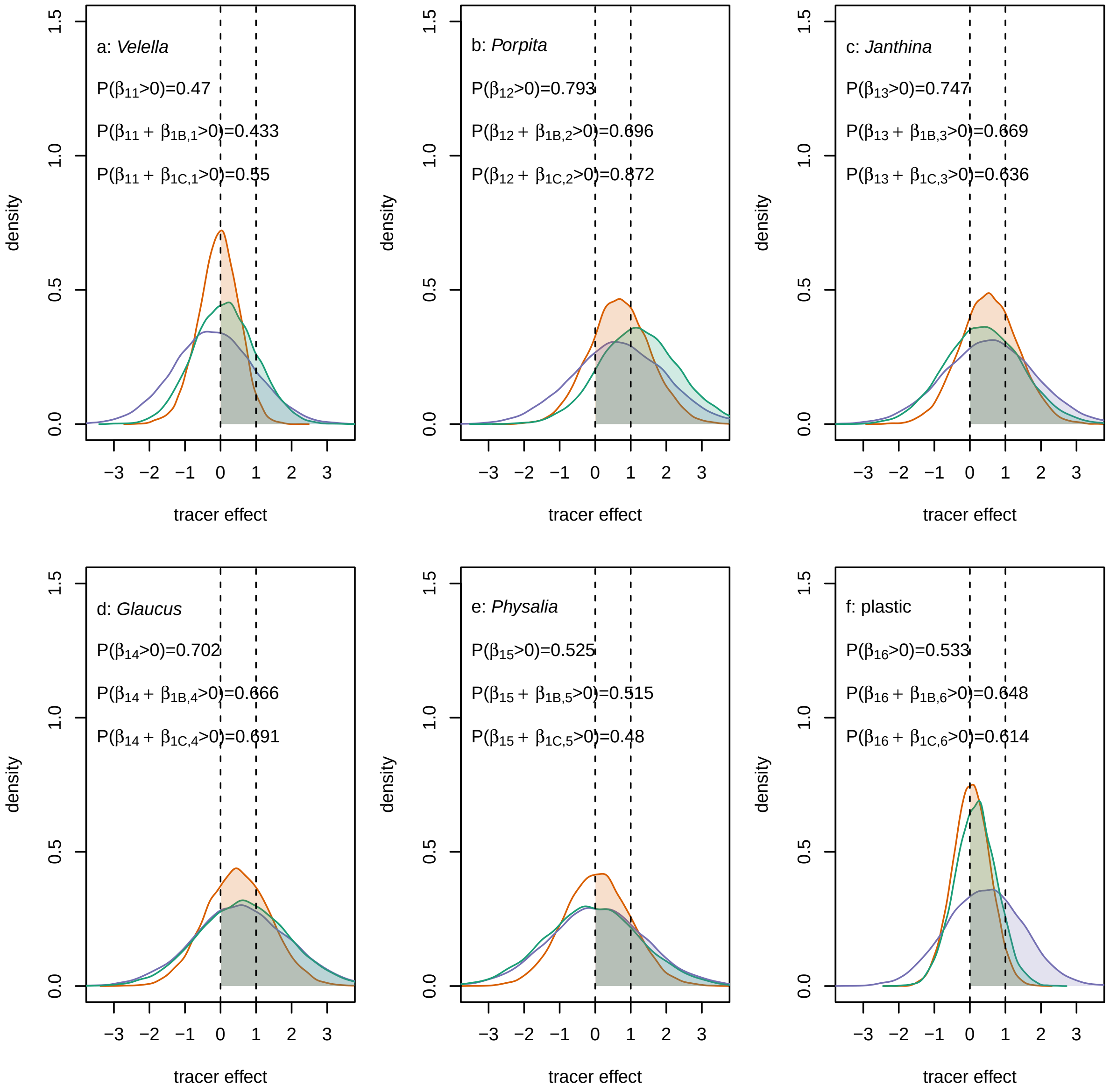

Figure Y: Effect of (centered and scaled) natural log tracer concentration on expected natural log of density (in numbers km^{-2}) for the Egger et al. [6] data for (a) *Velella*, (b) *Porpita*, (c) *Janthina*, (d) *Glaucus*, (e) *Physalia* and (f) plastic in areas A (orange, $\beta_{1,i}$), B (purple, $\beta_{1,i} + \beta_{1B,i}$) and C (green, $\beta_{1,i} + \beta_{1C,i}$). Kernel density estimates of posterior distributions, with posterior probability that the effect is positive given on each panel. Vertical dashed lines at 0 and 1, physically important values for the effect. The data underlying this Figure can be found in S1 Data.

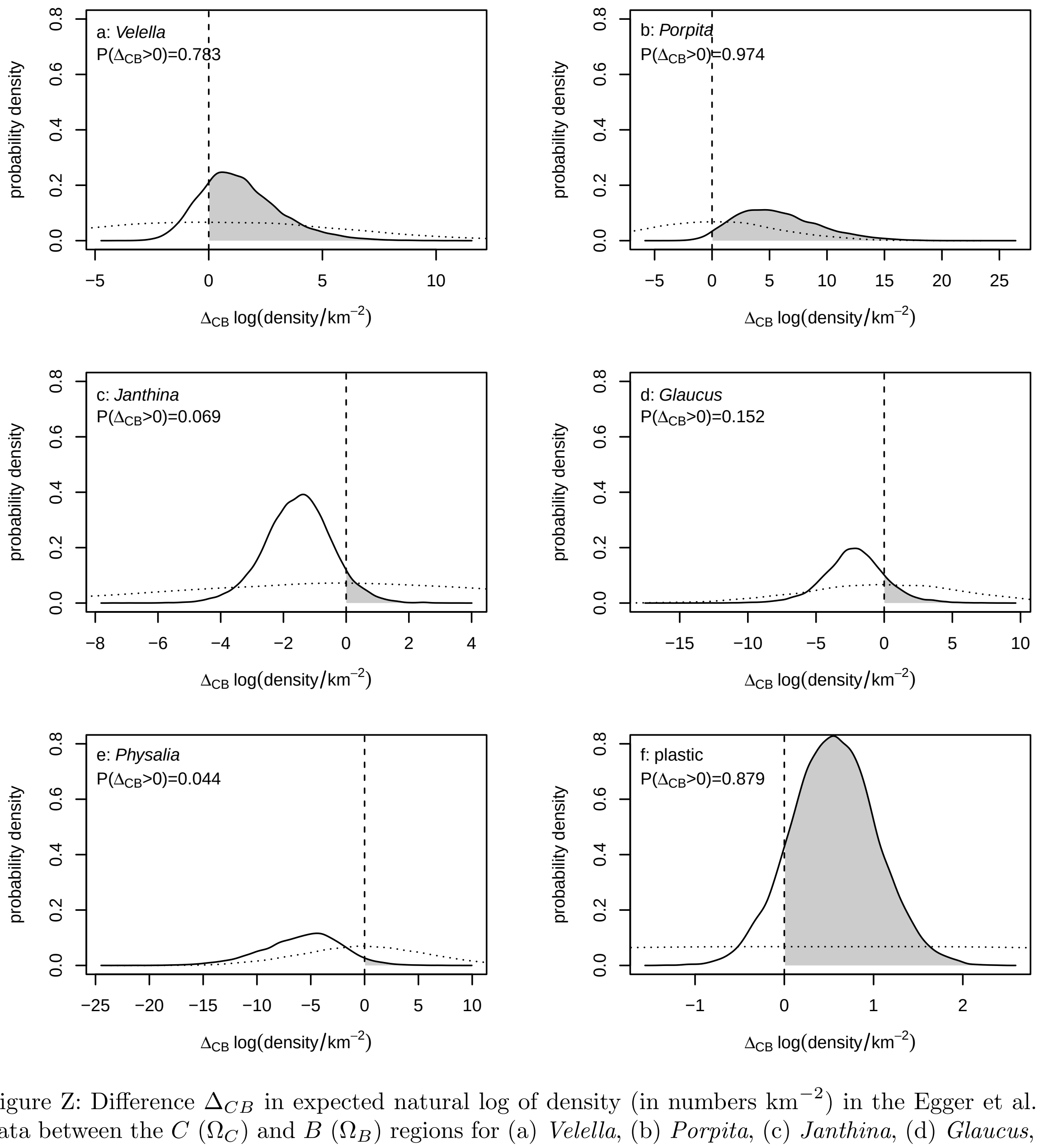

Figure Z: Difference Δ_{CB} in expected natural log of density (in numbers km^{-2}) in the Egger et al. [6] data between the C (Ω_C) and B (Ω_B) regions for (a) *Velella*, (b) *Porpita*, (c) *Janthina*, (d) *Glaucus*, (e) *Physalia* and (f) plastic. Kernel density estimates of posterior distributions, with posterior probability that the difference is positive given on each panel. Dotted lines are kernel density estimates of the prior distribution for each difference. The data underlying this Figure can be found in S1 Data.

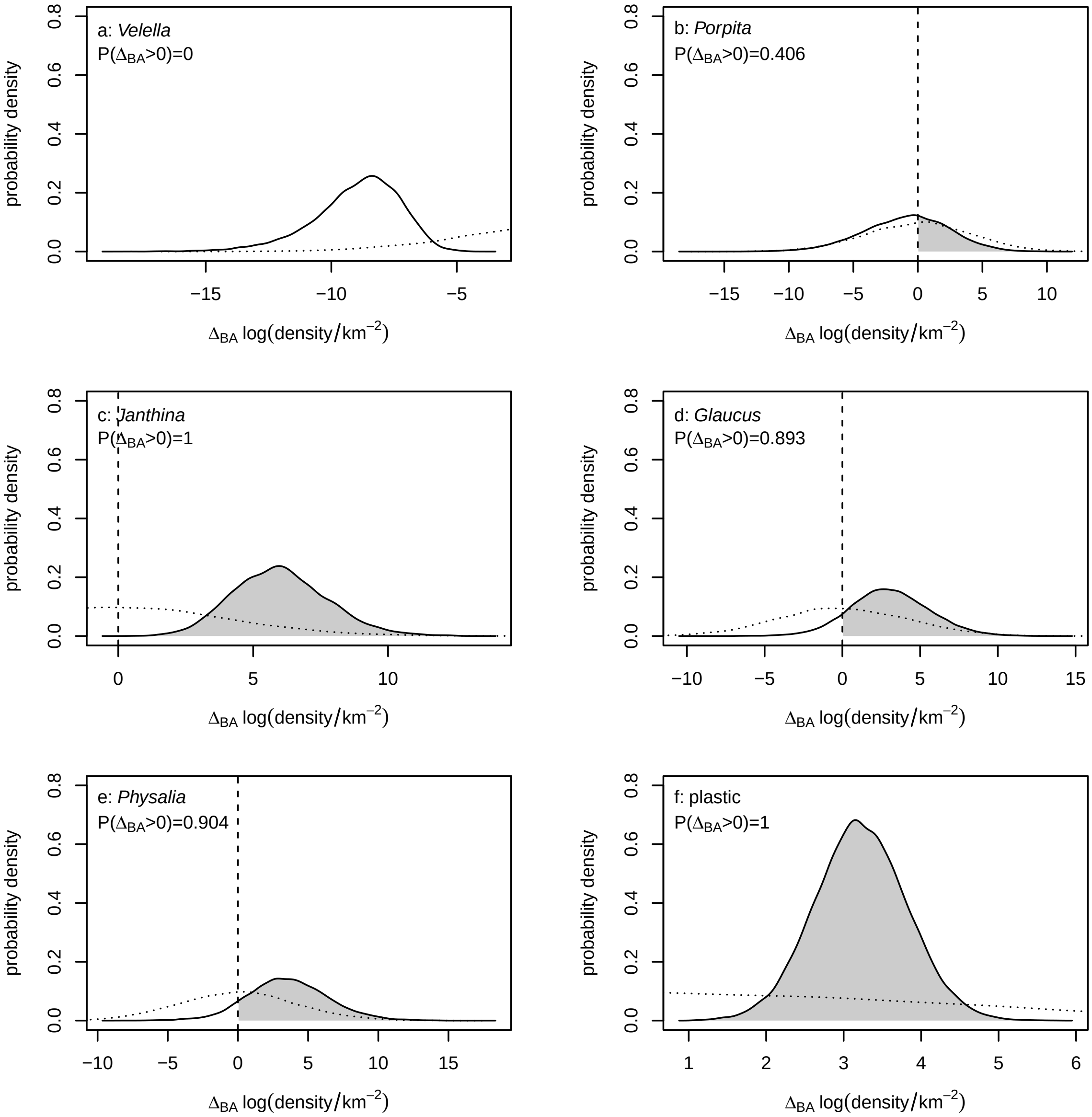

Figure AA: Difference Δ_{BA} in expected natural log of density (in numbers km^{-2}) in the Egger et al. [6] data between the B (Ω_B) and A (Ω_A) regions for (a) *Velella*, (b) *Porpita*, (c) *Janthina*, (d) *Glaucus*, (e) *Physalia* and (f) plastic. Kernel density estimates of posterior distributions, with posterior probability that the difference is positive given on each panel. Dotted lines are kernel density estimates of the prior distribution for each difference. The data underlying this Figure can be found in S1 Data.

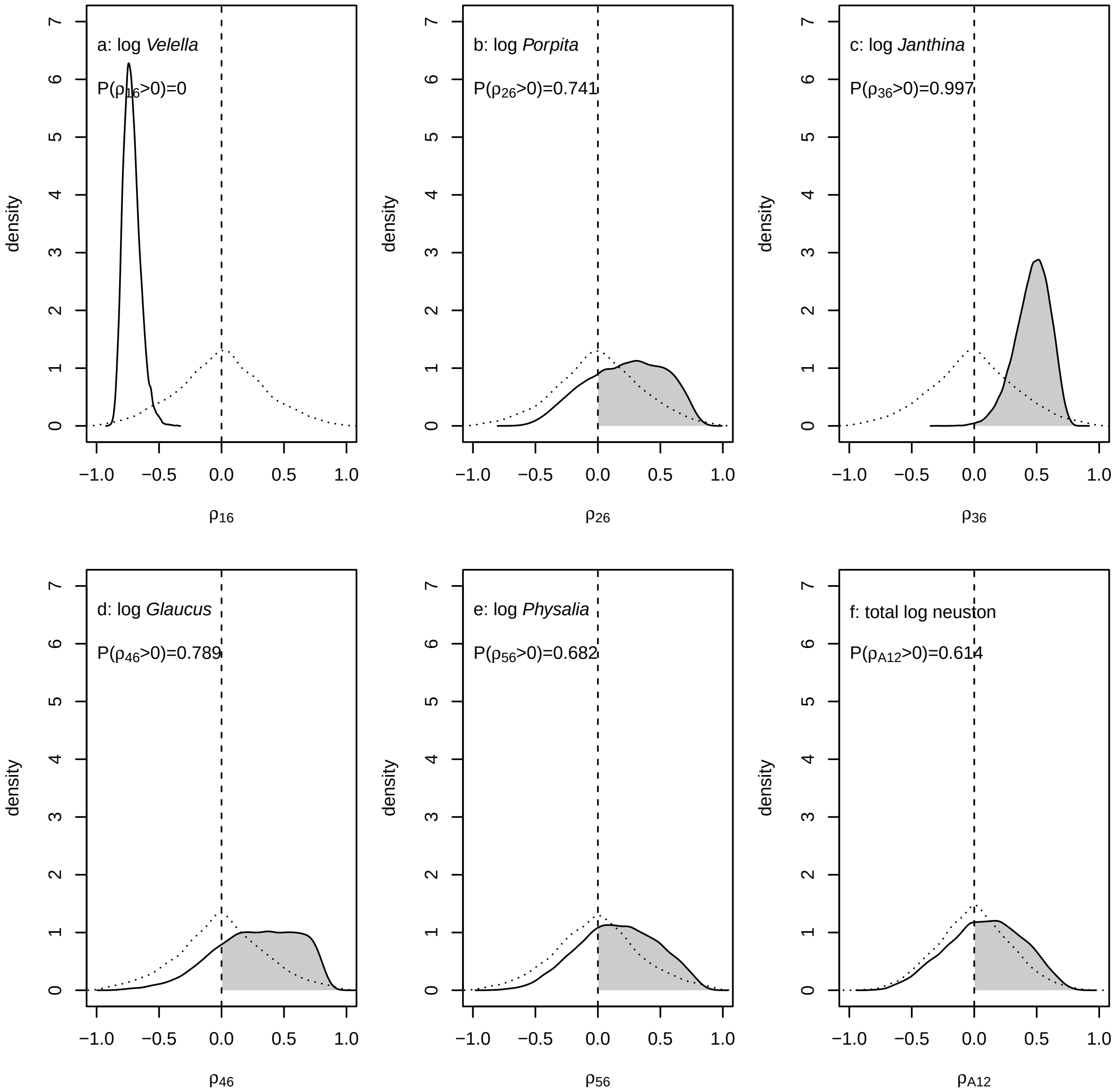

Figure AB: Posterior densities of marginal correlations ρ in the Egger et al. [6] data over the entire region Ω_{all} between log plastic density and log densities of a: *Velella*, b: *Porpita*, c: *Janthina*, d: *Glaucus*, e: *Physalia* and f: total log neuston. Kernel density estimates, with vertical dashed lines at zero. Posterior probability that each marginal correlation is positive is indicated. Dotted lines are kernel density estimates of the prior distribution for each correlation. Based on 2019 tracer data. The data underlying this Figure can be found in S1 Data.

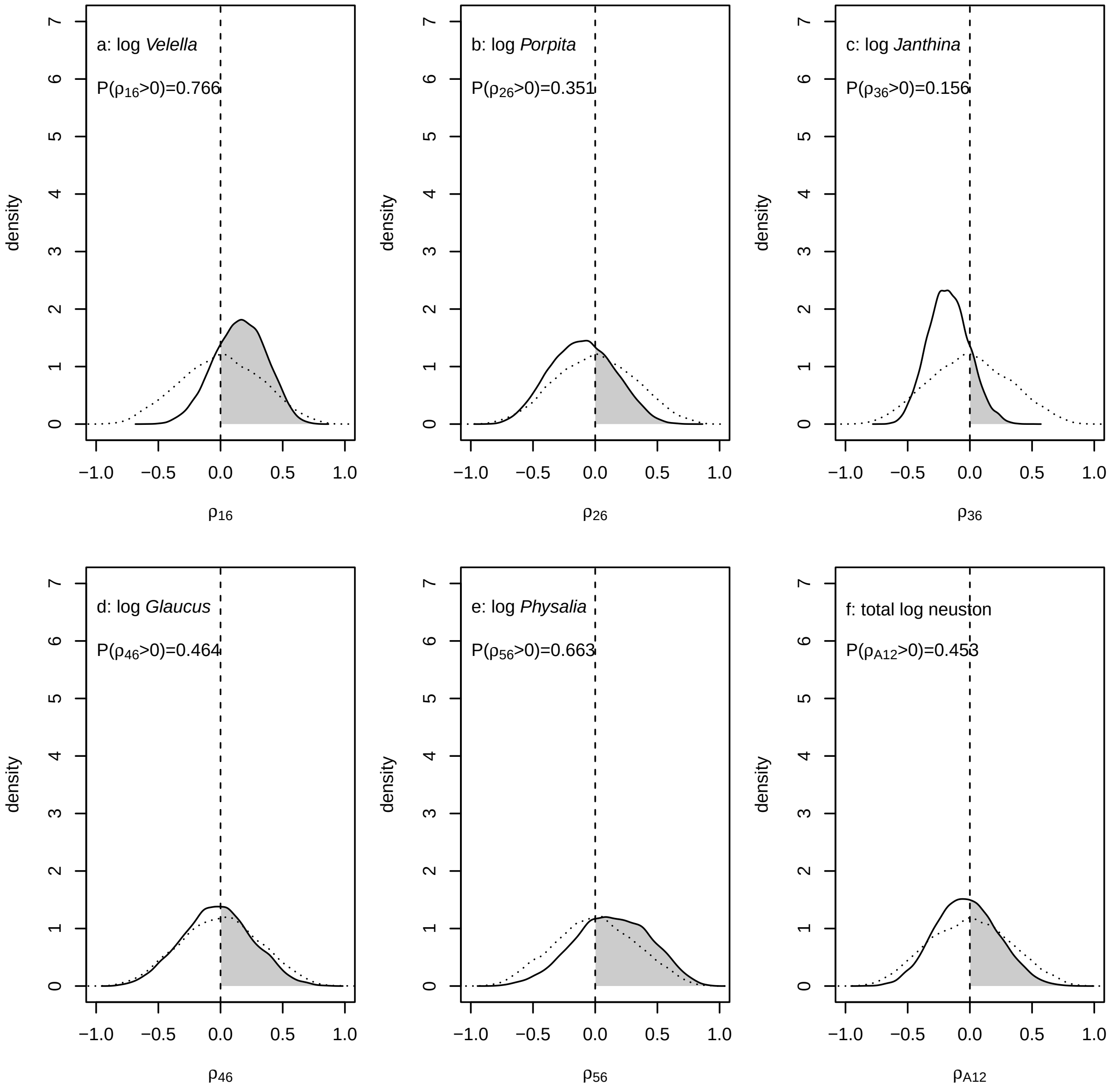

Figure AC: Posterior densities of marginal correlations ρ in the Egger et al. [6] data over the region Ω_C between log plastic density and log densities of a: *Velella*, b: *Porpita*, c: *Janthina*, d: *Glaucus*, e: *Physalia* and f: total log neuston. Kernel density estimates, with vertical dashed lines at zero. Posterior probability that each marginal correlation is positive is indicated. Dotted lines are kernel density estimates of the prior distribution for each correlation. Based on 2019 tracer data. The data underlying this Figure can be found in S1 Data.

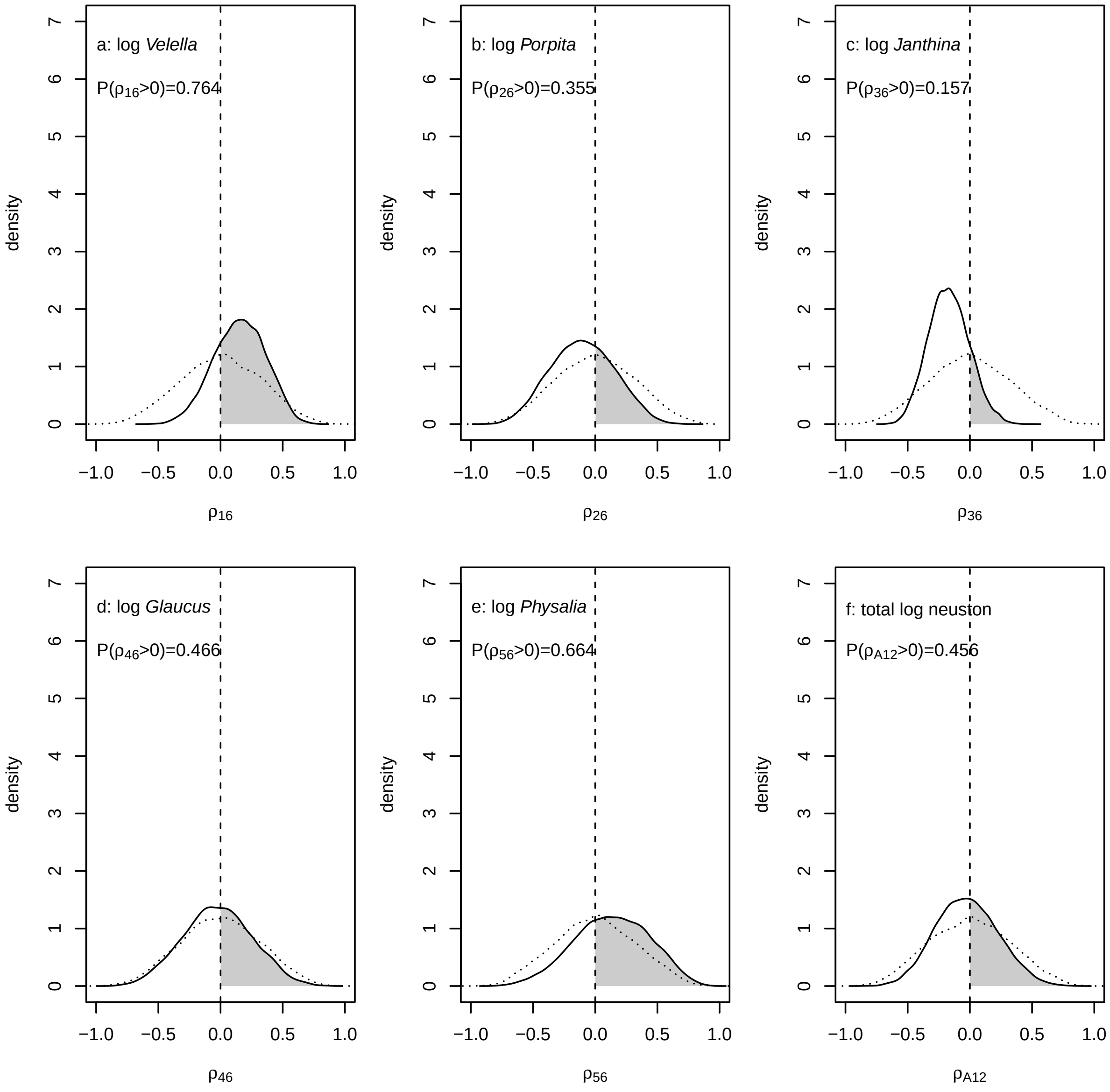

Figure AD: Posterior densities of marginal correlations ρ in the Egger et al. [6] data over the region Ω_B between log plastic density and log densities of a: *Velella*, b: *Porpita*, c: *Janthina*, d: *Glaucus*, e: *Physalia* and f: total log neuston. Kernel density estimates, with vertical dashed lines at zero. Posterior probability that each marginal correlation is positive is indicated. Dotted lines are kernel density estimates of the prior distribution for each correlation. Based on 2019 tracer data. The data underlying this Figure can be found in S1 Data.

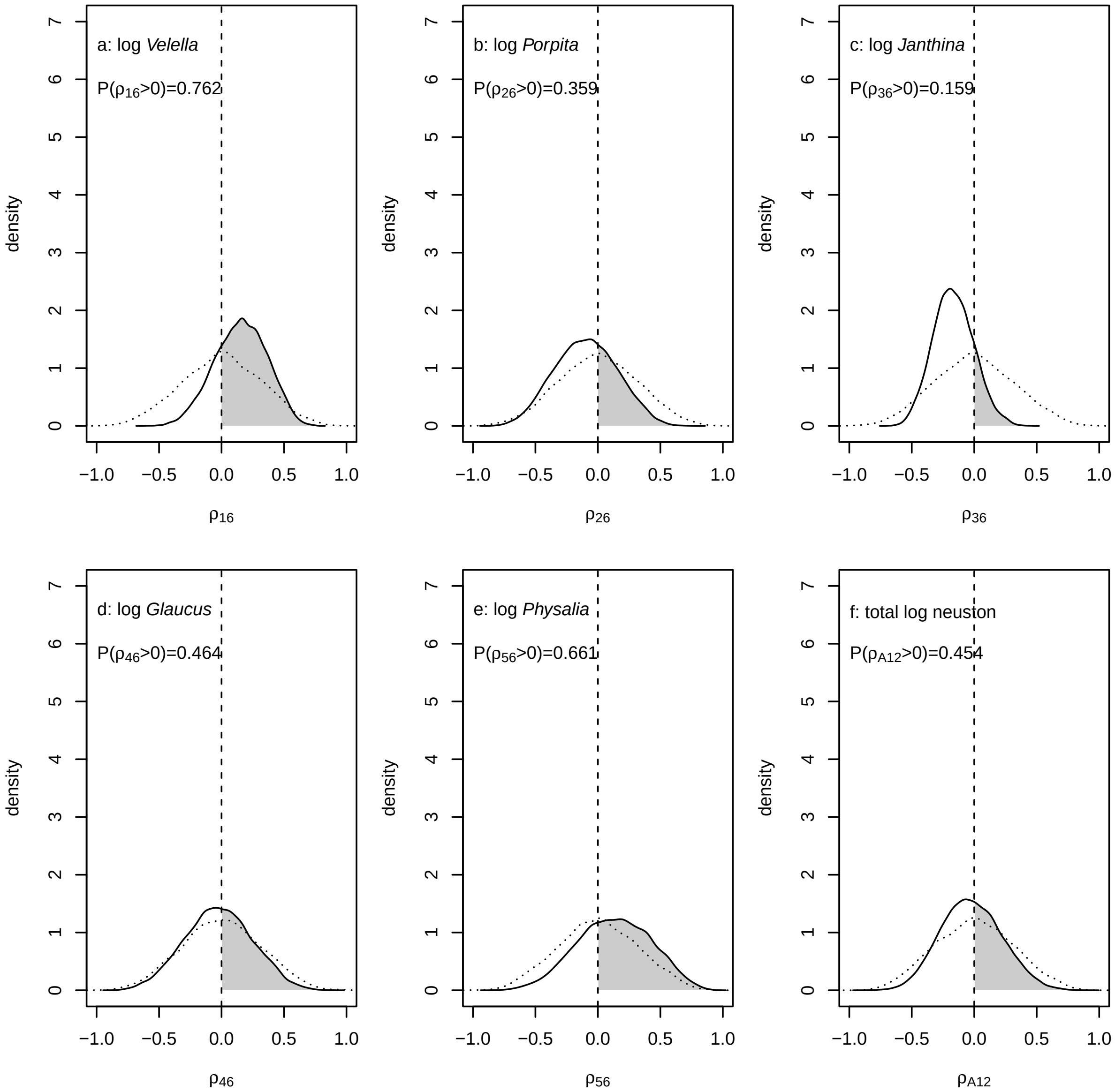

Figure AE: Posterior densities of marginal correlations ρ in the Egger et al. [6] data over the region Ω_A between log plastic density and log densities of a: *Velella*, b: *Porpita*, c: *Janthina*, d: *Glaucus*, e: *Physalia* and f: total log neuston. Kernel density estimates, with vertical dashed lines at zero. Posterior probability that each marginal correlation is positive is indicated. Dotted lines are kernel density estimates of the prior distribution for each correlation. Based on 2019 tracer data. The data underlying this Figure can be found in S1 Data.

Table B: Parameter estimates for the model defined by Equation H fitted to data from Egger et al. [6]: intercept $\boldsymbol{\beta}_0$, tracer effect $\boldsymbol{\beta}_1$, patch effects $\boldsymbol{\beta}_B$ and $\boldsymbol{\beta}_C$, interaction effect $\boldsymbol{\beta}_{1B}$ and $\boldsymbol{\beta}_{1C}$, rows of lower triangle of covariance matrix $\boldsymbol{\Sigma}$ of observation-level random effects. Columns are taxa and plastic. Each cell contains the posterior mean, with marginal 95 % credible highest density regions in parentheses. For two elements of row 5 of $\boldsymbol{\Sigma}$, the highest density region consists of multiple disjoint intervals.

	Velella	*Porpita*	*Janthina*	*Glaucus*	*Physalia*	plastic
$\boldsymbol{\beta}_0$	11.27 (9.72, 12.83)	-0.84 (-5.80, 4.05)	1.12 (-2.21, 4.51)	-1.18 (-7.31, 4.89)	-2.69 (-9.74, 4.06)	8.82 (7.38, 10.28)
$\boldsymbol{\beta}_1$	-0.06 (-1.19, 1.07)	0.70 (-1.00, 2.38)	0.55 (-1.06, 2.16)	0.49 (-1.31, 2.29)	0.06 (-1.77, 1.90)	0.04 (-1.00, 1.08)
$\boldsymbol{\beta}_B$	-8.87 (-12.42, -5.26)	-1.53 (-7.87, 4.77)	5.52 (1.82, 9.25)	2.58 (-2.52, 7.67)	3.51 (-2.14, 9.13)	3.04 (1.20, 4.88)
$\boldsymbol{\beta}_C$	-7.64 (-10.16, -5.12)	3.45 (-1.71, 8.46)	3.89 (-0.13, 7.90)	0.13 (-5.01, 5.31)	-2.21 (-8.63, 4.33)	3.62 (1.67, 5.56)
$\boldsymbol{\beta}_{1B}$	-0.13 (-2.09, 1.83)	-0.02 (-1.99, 1.95)	0.00 (-1.94, 1.94)	0.09 (-1.86, 2.04)	-0.00 (-1.96, 1.95)	0.37 (-1.50, 2.25)
$\boldsymbol{\beta}_{1C}$	0.18 (-1.41, 1.76)	0.55 (-1.22, 2.32)	-0.16 (-1.92, 1.60)	0.15 (-1.74, 2.03)	-0.12 (-2.05, 1.80)	0.13 (-1.19, 1.44)
$\boldsymbol{\sigma}_1$	1.38 (0.40, 2.51)					
$\boldsymbol{\sigma}_2$	-0.13 (-1.28, 0.99)	2.69 (-0.08, 7.03)				
$\boldsymbol{\sigma}_3$	-0.06 (-1.51, 1.40)	0.44 (-1.67, 2.64)	6.05 (1.91, 11.08)			
$\boldsymbol{\sigma}_4$	-0.06 (-2.59, 2.25)	-0.18 (-3.56, 2.61)	-0.14 (-4.48, 3.85)	8.17 (-0.48, 36.35)		
$\boldsymbol{\sigma}_5$	0.07 (-2.34, 2.38)	0.02 (-3.12, 3.38)	1.01 (-2.86, 6.22)	-0.16 (-6.78, -6.77) (-5.34, 4.86)	8.28 (-0.60, 33.51) (38.90, 39.13)	
$\boldsymbol{\sigma}_6$	0.20 (-0.38, 0.79)	-0.17 (-1.22, 0.86)	-0.49 (-1.53, 0.53)	-0.07 (-1.91, 1.83)	0.45 (-1.37, 2.85)	1.37 (0.78, 1.95)

References

[1] J. Aitchison and C. H. Ho. The multivariate Poisson-log normal distribution. *Biometrika*, 76: 643–653, 1989.

[2] B. Carpenter, A. Gelman, M. D. Hoffman, D. Lee, B. Goodrich, M. Betancourt, M. Brubaker, J. Guo, P. Li, and A. Riddell. Stan: a probabilistic programming language. *Journal of Statistical Software*, 76:1–32, 2017. doi: 10.18637/jss.v076.i01.

[3] G. Casella and R. L. Berger. *Statistical inference*. Duxbury, Pacific Grove, CA, second edition, 2002.

[4] E. A. Catchpole and B. J. T. Morgan. Detecting parameter redundancy. *Biometrika*, 84:187–196, 1997.

[5] Matthias Egger, Rein Nijhof, Lauren Quiros, Giulia Leone, Sarah-Jeanne Royer, Andrew C McWhirter, Gennady A Kantakov, Vladimir I Radchenko, Evgeny A Pakhomov, Brian P V Hunt, and Laurent Lebreton. A spatially variable scarcity of floating microplastics in the eastern North Pacific Ocean. *Environmental Research Letters*, 15:114056, 2020.

[6] Matthias Egger, Lauren Quiros, Giulia Leone, Francesco Ferrari, Christiana M. Boerger, and Michelle Tishler. Relative abundance of floating plastic debris and neuston in the Eastern North Pacific Ocean. *Frontiers in Marine Science*, 8:626026, 2021.

[7] M. A. Hamdan and C. P. Tsokos. A model for physical and biological problems: the bivariate-compounded Poisson distribution. *Review of the International Statistical Institute*, 39:60–63, 1971.

[8] Matthew D. Hoffman and Andrew Gelman. The No-U-Turn Sampler: Adaptively setting path lengths in Hamiltonian Monte Carlo. *Journal of Machine Learning Research*, 15:1351–1381, 2014.

[9] N. L. Johnson, S. Kotz, and N. Balakrishnan. *Discrete Multivariate Distributions*. John Wiley & Sons, Inc., New York, 1997.

[10] D. Lewandowski, D. Kurowicka, and H. Joe. Generating random correlation matrices based on vines and extended onion method. *Journal of Multivariate Analysis*, 100:1989–2001, 2009.

[11] R Core Team. *R: A Language and Environment for Statistical Computing*. R Foundation for Statistical Computing, Vienna, Austria, 2022. URL `https://www.R-project.org/`.

[12] A. I. Savilov. Pleuston of the Pacific Ocean. In L. A. Zenkevich, editor, *Biology of the Pacific Ocean*, pages 318–426. U.S. Naval Oceanographic Office, Washington, D.C., 1970.

[13] Stan Development Team. Stan modeling language users guide and reference manual, version 2.30, 2022. URL `https://mc-stan.org/docs/stan-users-guide`.

[14] Stan Development Team. RStan: the R interface to Stan, 2022. URL `https://mc-stan.org/`. R package version 2.21.5.

[15] Sean Talts, Michael Betancourt, Daniel Simpson, Aki Vehtari, and Andrew Gelman. Validating Bayesian inference algorithms with simulation-based calibration. *arXiv e-prints*, art. arXiv:1804.06788, 2018. arXiv:1804.06788.

[16] A. Vehtari, A. Gelman, and J. Gabry. Practical Bayesian model evaluation using leave-one-out cross-validation and WAIC. *Statistics and Computing*, 27:1413–1432, 2017.
